# Commensal *Pseudomonas* strains facilitate protective response against pathogens in the host plant

**DOI:** 10.1038/s41559-022-01673-7

**Published:** 2022-02-24

**Authors:** Or Shalev, Talia L. Karasov, Derek S. Lundberg, Haim Ashkenazy, Pratchaya Pramoj Na Ayutthaya, Detlef Weigel

**Affiliations:** 1 Department of Molecular Biology, Max Planck Institute for Biology Tübingen, Tübingen, Germany; 2 Present Address: Systems Biology of Microbial Communities, University of Tübingen, Tübingen, Germany; 3 Present Address: School of Biological Sciences, University of Utah, Salt Lake City, UT USA; 4 Present Address: Department of Civil, Environmental and Geomatic Engineering, Swiss Federal Institute of Technology (ETH) Zurich, Zurich, Switzerland

**Keywords:** Microbial ecology, Microbial communities, Natural variation in plants, Plant symbiosis

## Abstract

The community structure in the plant-associated microbiome depends collectively on host–microbe, microbe–microbe and host–microbe–microbe interactions. The ensemble of interactions between the host and microbial consortia may lead to outcomes that are not easily predicted from pairwise interactions. Plant–microbe–microbe interactions are important to plant health but could depend on both host and microbe strain variation. Here we study interactions between groups of naturally co-existing commensal and pathogenic *Pseudomonas* strains in the *Arabidopsis thaliana* phyllosphere. We find that commensal *Pseudomonas* prompt a host response that leads to selective inhibition of a specific pathogenic lineage, resulting in plant protection. The extent of protection depends on plant genotype, supporting that these effects are host-mediated. Strain-specific effects are also demonstrated by one individual *Pseudomonas* isolate eluding the plant protection provided by commensals. Our work highlights how within-species genetic differences in both hosts and microbes can affect host–microbe–microbe dynamics.

## Abstract (web summary)

The authors conduct competition experiments with multiple strains of *Pseudomonas* (some pathogenic and some commensal) in the phylosphere microbiome of *Arabidopsis* plants, showing that both the host and the commensal strains interact to inhibit the pathogenic strains.

## Main

Plants, like other complex organisms, host a diverse set of microbes. The assembly of these microbial communities is shaped both by host–microbe and microbe–microbe interactions. These interactions may be of any symbiotic type, mutualistic, commensal or parasitic and are dictated by the balance of inhibition and facilitation of growth by both the host and other microbes. As has been exemplified in many studies, interactions between organisms are dynamic, depending on evolutionary history^1,2^ and the current biotic^3,4^ and abiotic^5,6^ environment.

Many facets of plant–microbe interactions have been studied in considerable detail, not least because of their implications for agriculture and ecology. Colonization of the plant depends on the ability of microbes to proliferate on and in the host but also on the ability of the host to promote or restrict microbial growth. In the case of pathogens, there is often a co-evolutionary arms race, in which plants evolve recognition and immune tools to control the microbes, while microbes evolve evasion and an offensive arsenal to further populate the plant^7,8^. These co-evolutionary dynamics typically fuel the generation of genetic diversity within both host and microbe, and the dependence of microbial colonization and host health on intraspecific variation has been widely documented^1,9–11^. Nonetheless, the extent to which intraspecific host variation shapes the composition of its microbiota appears to be comparatively small^5,6^, with the most dramatic effects seen for specific taxa that are recognized by the immune system^12,13^. In contrast, abiotic factors^5,6^ and local reservoirs of microbes have a large influence on the composition of microbial communities^3,14,15^.

Colonizing microbes exert differential effects on host health—from harmful^16^ to beneficial^17^. These effects are often related to microbial load because overpopulation of the plant by microbes can negatively impact its health^9,18^. This raises questions about the ability of the host plant to differentially recognize and respond to a consortium of microbes with a range of functions, that is, differentiating friend from foe in a complex assembly of microbial taxa. The numerous constraints resulting from multiple host–microbe and microbe–microbe interrelations create a complex system of relationships, making extrapolation of rules from simplified systems likely difficult. For example, overpopulation of the plant by one microbe can result in negative health impacts, but these might be mitigated in the presence of other microbes^17,19^. As one example, interactions between different bacterial taxa have been shown to affect host root development^20,21^.

Whereas studies of microbe–microbe interactions in planta have paved the way for important findings regarding their impact on the host^20,21^ or overall community^14,15^, the effect of the host on such microbial interactions has been considered less often^22^. A powerful tool for establishing causality in microbe–microbe and plant–microbe interactions is provided by synthetic communities^23^. A limitation has been the ability to distinguish closely related taxa in either natural or synthetic microbial communities, which are usually profiled using 16S rDNA sequences. Until recently, strains with similar but not identical 16S rDNA segments have been collapsed as Operational Taxonomic Units (OTUs). However, even strains with identical 16S rDNA sequences can differ functionally due to variation in gene content^24–26^, not least because of ubiquitous horizontal gene transfer^27^, reflecting that 16S rDNA sequences evolve (much) more slowly than the rest of the genome. The role of fine-grained taxonomic differences has often been overlooked in the context of characterizing and analysing plant microbiota, limiting our understanding of how genetic variation among closely related strains affects plant–microbe–microbe interactions.

In a previous study, Karasov and colleagues^11^ surveyed *Pseudomonas* populations from leaves of wild *Arabidopsis thaliana* plants in southwest Germany. Among these, one lineage, which was highly pathogenic in axenic infections, often dominated endophytic microbial communities of *A. thaliana* leaves. This lineage was isolated from plants without any visible disease symptoms, suggesting that other factors, including co-colonizing microbes, were mitigating its pathogenic potential in nature. Candidates for such effects include other co-occurring *Pseudomonas* lineages that did not appear to have any substantial impacts on host health when tested individually.

Here we took advantage of this collection of wild *Pseudomonas* isolates^11^ to investigate intraspecific host–microbe–microbe dynamics by infecting *A. thaliana* plants with synthetic communities. Employing genome barcoding, we were able to track individual isolates in multi-strain consortium contexts regardless of their genome-wide similarity. Specifically, we examined interactions between different pathogenic and commensal *Pseudomonas* strains with the host leaves and among each other, and the linkage of these to host health. We found that the host facilitated protective commensal–pathogen interactions, revealing complex interactions that could not easily be detected by studying pairwise host–microbe or microbe–microbe relationships.

## Results

### Barcoding of *Pseudomonas* isolates and experimental design

**Fig. 1:**
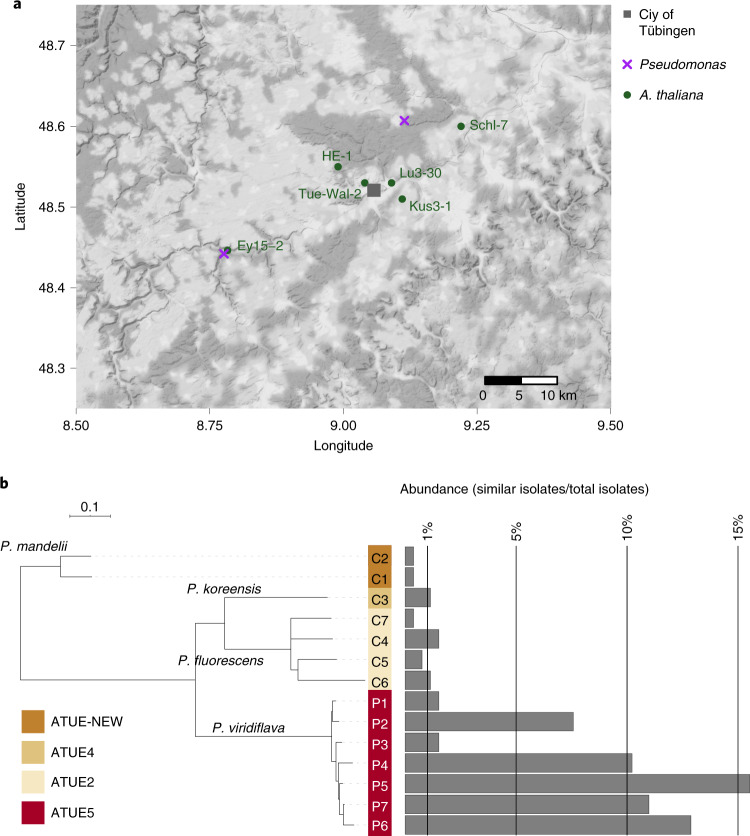
Study system. **a**, Location of original **a**. *thaliana* and *Pseudomonas* sampling sites around Tübingen. **b**, Taxonomic representation of the 14 *Pseudomonas* isolates used and the prevalence of closely related strains (divergence <0.0001 in core genome) among the 1,524 isolates of the Karasov collection^11^. Taxonomic assignment is indicated for each ATUE group, corresponding to a specific OTU in the Karasov collection^11^. ‘P’ refers to pathogen candidate and ‘C’ to commensal candidate. The scale bar denotes 0.1 nucleotide substitutions per site.

To test possible host–commensal–pathogen dynamics in a local population, we spray inoculated six *A. thaliana* accessions with synthetic bacterial communities composed of pathogenic and commensal *Pseudomonas* candidates. Because we wanted to study interactions that are likely to occur in nature, we used *A. thaliana* genotypes that originated from the same plant populations near Tübingen, Germany^28^, from which the *Pseudomonas* strains had been isolated (Fig. 1a). Classification of *Pseudomonas* lineages as pathogenic or commensal was based on observed effects in axenic infections^11^. Only one lineage, previously named OTU5, which dominated local plant populations, was associated with pathogenicity, both based on negative impact on rosette weight and visible disease symptoms^11^. We henceforth call this lineage ATUE5 (isolates sampled from ‘Around TUEbingen, group 5’) and all other *Pseudomonas* lineages from the Karasov collection non-ATUE5. We interchangeably use the terms ‘pathogens’ or ‘ATUE5’, and ‘commensals’ or ‘non-ATUE5’.

**Extended Data Fig. 1:**
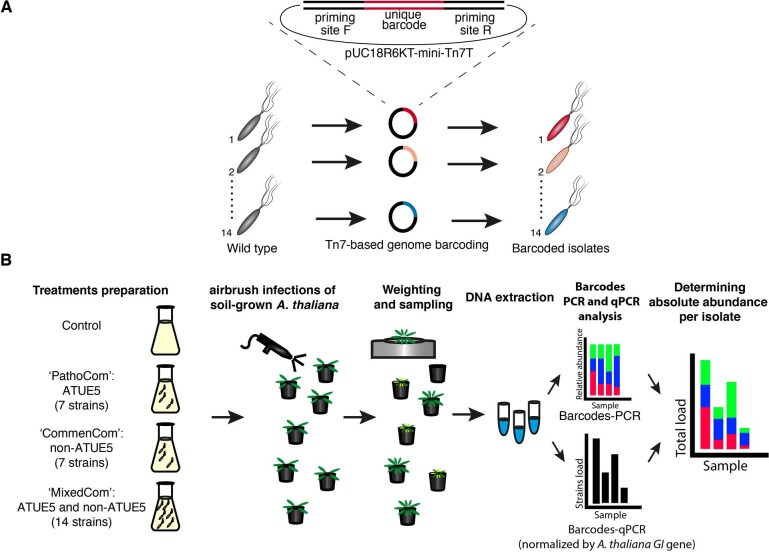
Experimental design. **a**. Diagram of bacterial barcoding. **b**. Diagram of treatments and sampling.

Seven pathogenic *Pseudomonas* and seven commensal isolates were selected, prioritizing those with the highest prevalence in the field collection^11^ (Fig. 1b), estimated from the number of similar isolates with nucleotide sequence divergence of less than 0.0001 in their core genome. The 14 *Pseudomonas* isolates were classified as belonging to four OTUs based on 16S rDNA clustering at 99% sequence identity. Because of the high relatedness of several of the isolates, we could not rely upon a single endogenous genetic marker to distinguish them in a community context, and we therefore genome barcoded all of the isolates. We employed the mini-Tn7 system^29^ to insert a single copy of a 22 bp-long unique sequence, flanked by universal priming sites, into the chromosome of each isolate (Extended Data Fig. 1a). We validated the sequence of all barcodes in the corresponding isolates using Sanger sequencing (Supplementary Table 1) and confirmed correct integration by barcode-specific polymerase chain reaction (PCR, Supplementary Fig. 1a). Barcode amplification yielded the expected products on DNA extracted from infected *A. thaliana* individuals (Supplementary Fig. 1b). While barcoding slightly impaired the in vitro growth of isolates P3 and P4, most barcoded strains grew similarly well as the non-barcoded parental strains when tested in a lysogeny Broth (LB) medium (Supplementary Fig. 2).

Next, we constructed three synthetic communities using the barcoded isolates: an exclusively pathogenic community, comprising the seven ATUE5 isolates (PathoCom); an exclusively commensal community, comprising the seven non-ATUE5 isolates (CommenCom); and a joint community comprising all 14 isolates, both pathogens and commensals (MixedCom). Isolates were mixed in equal proportions (based on OD_600_ readout), and their absolute starting concentration was identical in each synthetic community. Thus, the inoculum of the MixedCom with 14 isolates had twice the total number of bacterial cells per volume as either the PathoCom or CommenCom inoculum.

The community experiments were conducted with plants grown on soil in the presence of other microbes acquired from the environment. We chose to perform non-axenic experiments rather than with axenically grown plants because infection outcomes on soil seemed more consistent with phenotypes observed in the field. Specifically, the focal bacterial strains had been isolated from wild plants that were not obviously diseased^11^. In the lab, axenic infections with these strains often had rapid and dramatic effects, killing plants as early as three days post infection (DPI) (Supplementary Fig. 3). In contrast, inoculated soil-grown plants had only mild disease symptoms and decreased size even 12 DPI (Supplementary Fig. 3). Also, to more closely mimic natural infections, which probably occur through the air, we chose to inoculate plants by spraying with bacterial suspensions rather than direct leaf infiltration, the more common method for testing the effects of leaf pathogenic bacteria in *A. thaliana*.

Twenty-one days after sowing, we spray inoculated rosettes of plants raised in growth chambers with the three synthetic communities or with a buffer (Control). At 12 DPI, we sampled the fresh rosettes, weighed them and extracted DNA from them. We measured absolute abundance of each isolate by coupling barcode-specific PCR and sequencing-based amplicon counting with quantitative PCR (qPCR). Including an amplicon from an *A. thaliana* gene in the qPCR assay allowed us to estimate absolute isolate abundances as the ratio of bacterial to plant cells (Extended Data Fig. 1b).

**Extended Data Fig. 2:**
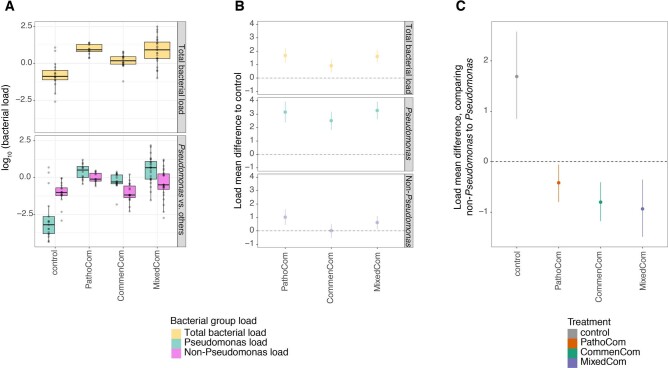
Both *Pseudomonas* and total bacterial load are driven primarily by synthetic communities. **a**. Cumulative bacterial load (ratio of bacterial and host read counts, which is dimensionless; Methods) in plant rosettes after control treatment or infection with each of the three synthetic communities. Cumulative load was analysed for (i) all bacterial reads classified at the genus level (‘total bacterial load’), (ii) all reads classified as ‘*Pseudomonas*’, and (iii) all reads not classified as ‘*Pseudomonas*’ at the genus level. Unclassified reads were discarded. **b**. Load mean differences between plants infected with each of the synthetic communities to control plants. (See panel A for absolute loads.) Mean difference was calculated per cumulative load type (Total bacterial load, *Pseudomonas* load and non-*Pseudomonas* load), using the model [log_10_ (Cumulative load) ~ treatment + error], that is, the same model was computed for all three data subsets. Dots indicate the median estimates, vertical lines 95% Bayesian credible intervals of the fitted parameter, and the dashed line the control baseline. **c**. Load mean difference between the cumulative non-*Pseudomonas* to *Pseudomonas* load in plants treated with control, or each of the three synthetic communities. Mean difference was calculated per treatment (control, PathoCom, CommenCom and MixedCom) using the model [log_10_ (Cumulative load) ~ Genus_classification + error] that is, the same model was computed for all four data subsets. The ‘Genus_classification’ predictor was set to either *Pseudomonas* or non-*Pseudomonas* Classification. Dots indicate the median estimates, vertical lines 95% Bayesian credible intervals of the fitted parameter, and the dashed line the *Pseudomonas* load baseline. DNA of Ey15-2 plants was randomly sampled across the two experiments, and among the four treatments. n=17 for control plants, n=12 for PathoCom-infected plants, n=18 for CommenCom-infected plants, and n=24 for MixedCom-infected plants. The V4 16S region, as well as the *A. thaliana* gene GIGANTEA were targeted^30^.

Because we used a non-sterile system, interactions of barcoded isolates with the plant or with each other could potentially be affected by the presence of other bacteria that colonized the plants from the environment. To gauge how important other environmental bacteria, especially other *Pseudomonas* strains, were, we quantitatively measured the total bacterial community profile based on the fourth hypervariable (V4) region of 16S rDNA, employing a recent method that measures not only community composition but also absolute bacterial load^30^. Although 16S rDNA-based profiling is not suitable to differentiate our *Pseudomonas* isolates from all other environmental *Pseudomonas* strains or from each other, comparing the uninfected with infected plants (among all three synthetic communities) showed that (1) environmental *Pseudomonas* load was small in uninfected plants and (2) total *Pseudomonas* load alone was higher than the cumulative load of all non-*Pseudomonas* bacteria in infected plants. We therefore conclude that cumulative bacterial load was mainly driven by the inoculated *Pseudomonas* strains in infected plants (Extended Data Fig. 2), suggesting that environmental microbes do not interfere in a specific manner with our system.

### Host effects on composition of synthetic communities

The six *A. thaliana* genotypes used were originally sampled from a maximum of 40 km apart^28^ in the same geographic region (Fig. 1a) and they also were all from the same host genetic group^31^. In accordance, we expected host genotype to have limited effects on the composition of our synthetic communities of co-occurring *Pseudomonas* isolates. While not large, there was nevertheless a significant effect of host genotype, explaining 5% to 12% of compositional variation in the different communities, as determined by permutational multivariate analysis of variance (PERMANOVA) with Bray–Curtis distances (Table 1). For comparison, the difference between experiments explained 4–26% of compositional variation. Analysis of similarities within each experiment indicated similar trends as PERMANOVA, with genotype having a significant effect on isolate composition in each synthetic community (Supplementary Table 2a).

**Table 1:** PERMANOVA of synthetic community composition in inoculated plants

Treatment	Df	Sum Sq	Pseudo-*F*	*R*^2^	*P*_r(>f)_	Variation source
**PathoCom**	5	4.83	4.67	0.12	**0.0005**	Geno
	1	1.68	8.11	0.04	**0.0005**	Exp
	5	1.08	1.04	0.03	0.3973	Geno:Exp
	158	32.68	NA	0.81	NA	Residuals
	169	40.27	NA	1.00	NA	Total
**CommenCom**	5	2.36	3.88	0.08	**0.0005**	Geno
	1	7.32	60.19	0.26	**0.0005**	Exp
	5	1.53	2.52	0.05	**0.0030**	Geno:Exp
	139	16.89	NA	0.60	NA	Residuals
	150	28.10	NA	1.00	NA	Total
**MixedCom**	5	2.17	1.99	0.05	**0.0020**	Geno
	1	6.29	28.89	0.13	**0.0005**	Exp
	5	2.03	1.86	0.04	**0.0020**	Geno:Exp
	170	36.99	NA	0.78	NA	Residuals
	181	47.47	NA	1.00	NA	Total

Analyses are based on Bray–Curtis distances of the 14 isolates, constrained by host genotype (Geno) and experiment (Exp) to estimate their effects on the explained variance. *n* = 170 for PathoCom, *n* = 151 for CommenCom, and *n* = 182 for MixedCom. Statistically significant relationships (*P* <0.05) are in bold. Df, degrees of freedom; Sum Sq, sum of squares; F, F statistic; NA, not available.

**Extended Data Fig. 3:**
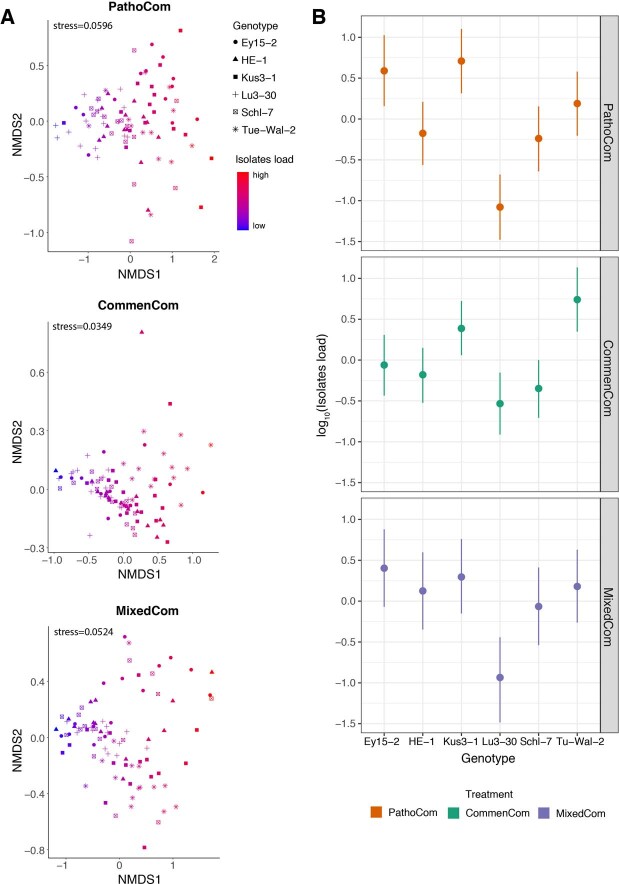
Comparison of composition and load of the 14 barcoded isolates on different *A. thaliana* genotypes. **a**. Nonmetric multidimensional scaling (NMDS) based on Bray-Curtis distances between six *A. thaliana* genotypes, in one representative experiment (October 2018). Each synthetic community was analysed separately. The abundance of all 14 barcoded isolates was considered, also among PathoCom and CommenCom to account for cross contaminations and technical distortions. Shapes denote the different genotypes, and bacterial load is indicated from blue to red. **b**. Isolate load of the six *A. thaliana* genotypes, among the three synthetic communities. Isolate load was defined as the cumulative abundance of all barcoded isolates that composed a synthetic community. Dots indicate the median estimates, and vertical lines 95% Bayesian credible intervals of the fitted parameter, following the model [log_10_(isolate load) ~ genotype + experiment + error].

We then examined bacterial composition clustering according to host genotype by applying multi-level comparison using pairwise ‘adonis’ based on Bray–Curtis distances. Some pairs of genotypes differed in their effects on all three communities (Supplementary Table 2b), an observation that was supported by non-metric multi-dimensional scaling (NMDS) ordination of bacterial composition in each treatment (Extended Data Fig. 3a). The cumulative load of all isolates was associated with the loading on the NMDS1 axis (Pearson’s *r* >0.99 and *P* value <2.2 × 10^−16^ for all three communities), suggesting that a part of the compositional differences between host genotypes was due to absolute rather than relative abundance. In agreement, we observed differences in total bacterial load among the host genotypes, and the nature of the differences was treatment dependent (Extended Data Fig. 3b).

### Host-dependent pathogenicity, growth promotion or protection

**Fig. 2:**
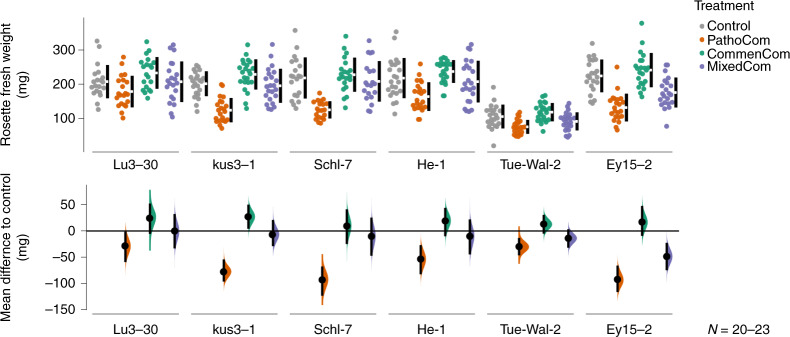
Commensal *Pseudomonas* isolates protect the plant in a host genotype-dependent manner. The six *A. thaliana* genotypes were treated with Control, PathoCom, CommenCom and MixedCom. Fresh rosette weight was measured 12 DPI. The top panel shows raw data; the breaks in the black vertical lines denote the mean value of each group, and the vertical lines indicate standard deviation. The bottom panel shows mean difference to control (in mg), inferred from bootstrap sampling^55,56^, indicating the distribution of effect sizes that are compatible with the data. The 95% confidence intervals are indicated by black vertical bars, and *n* = 20–23. Shown here are the results of one experiment. A second experiment gave similar results, as detailed in Supplementary Fig. 4.

**Extended Data Fig. 4:**
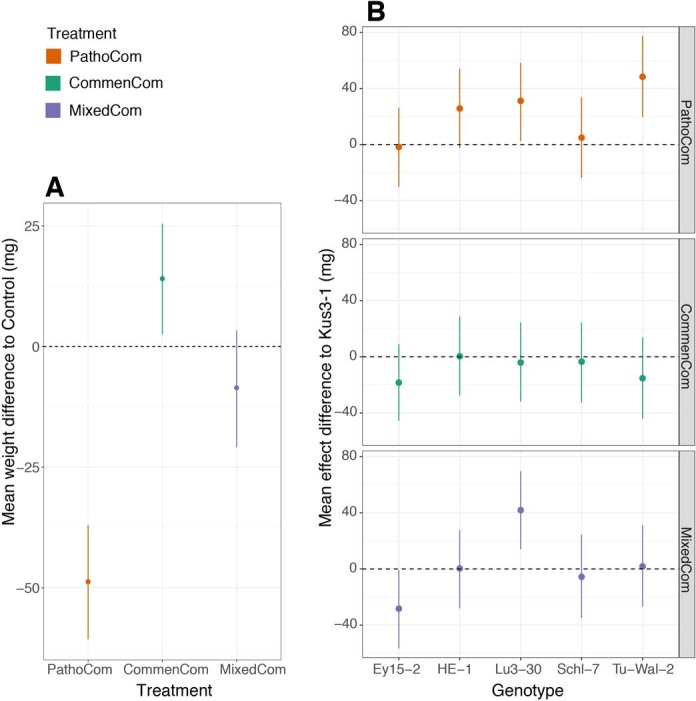
Effects of treatment and treatment-by-genotype on fresh rosette weight. Both effects were assessed using the model [weight ~ treatment * genotype + treatment + genotype + experiment + error]. **a**. Mean weight difference of plants infected with each of the three synthetic communities relative to control - that is, the treatment coefficients. **b**. Mean treatment effect differences between the six *A. thaliana* genotypes used in this study - that is, the treatment * genotype coefficients. Kus3-1 was randomly selected as a reference; dots indicate the median estimates, and vertical lines 95% Bayesian credible intervals of the fitted parameter.

Plant weight in our experiments was a function of treatment and host genotype, and interaction between the two, implying that the six *A. thaliana* accessions were differentially affected by similar treatments, as inferred from model comparison using leave-one-out cross validation and a two-way ANOVA test (Supplementary Table 3a,b). PathoCom infection reduced plant growth during the 12 days of the experiment (Fig. 2, Extended Data Fig. 4a and Supplementary Fig. 4), with weight decrease being the least in Lu3-30 and TueWal-2, indicating a certain level of resistance to PathoCom members in these accessions. The mean reduction to Control for the individual host genotypes was 29.1 mg (59.3, 1.4) for Lu3-30; 30.0 mg (46.4, 13.4) for TueWal-2; 77.2 mg (96.4, 54.2) for Kus3-1; 93.1 mg (123.5, 67.7) for Schl-7; 92.5 mg (116.4, 66.0) for Ey15-2; and 53.9 mg (82.6, 27.0) for HE-1 (95% confidence intervals in brackets, Extended Data Fig. 4b).

To validate that the effect of the PathoCom on plant weight was due to bacterial activity and not merely a host response to the inoculum (for example, pathogen-associated molecular pattern [‘PAMP’] triggered immunity), we infected plants with heat-killed PathoCom. We found a minor weight decrease in three out of the six accessions, but the overall contribution to weight reduction was small (Supplementary Fig. 5; heat-killed PathoCom accounts for 14% of the variation explained by the living PathoCom in the model shown).

In contrast to PathoCom, infections with CommenCom led to a slight increase in fresh weight, suggesting plant growth promotion activity or alternatively, protection from resident environmental pathogens (Extended Data Fig. 4a). This effect was independent of the host genotype (Extended Data Fig. 4b).

Importantly, the negative effects of the PathoCom members were greatly reduced in the MixedCom experiment. Plants infected with MixedCom grew to a similar extent as the control, with the exception of the genotype Ey15-2, which continued to suffer a substantial weight reduction when infected by the mixed community, with a mean reduction relative to Control of 48.5 mg (74.8, 22.6; Fig. 2, Extended Data Fig. 4b and Supplementary Fig. 4). Nonetheless, the growth reduction of Ey15-2 was less than that caused by PathoCom. Hence, co-colonization of pathogenic *Pseudomonas* with commensals led to enhanced growth, with the exact extent depending on host genotype.

Because the pathogenic *Pseudomonas* strains used here are much more lethal when inoculated on sterile plants^11^, we wanted to test whether environmentally derived microbes affected the observed interactions in a specific manner. We therefore performed a similar experiment in an axenic system on MS agar. The major trends that we had observed on soil were recapitulated, including the protection against ATUE5 by CommenCom members and the reduced protection of Ey15-2 by CommenCom members against pathogens (Supplementary Fig. 6). This does not exclude that members of the environmental microbiota enhance or dampen some of the observed effects, but if they do, they do so in a general manner and they are not essential for the observed effects.

In aggregate, these results support the role of ATUE5 strains as pathogenic and provide additional evidence for protection against ATUE5 by commensal *Pseudomonas* strains that coexist with ATUE5 in nature. Next, we therefore wanted to learn whether and how changes in bacterial abundance or shifts in *Pseudomonas* community composition led to differential impacts on growth of the infected plants.

### Load-dependent impact of pathogens and commensals

**Fig. 3:**
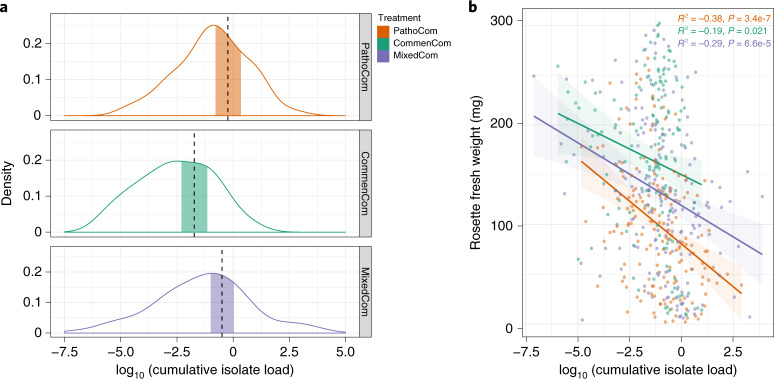
Plants are more tolerant to commensals than to pathogens. **a**, Density plot of log_10_(bacterial load) for the three synthetic communities. Vertical dashed lines indicate means, and the shaded areas represent 95% Bayesian credible intervals of the fitted parameter, following the model [log_10_(bacterial load) ~ treatment + genotype + experiment + error]. Load was computed as the ratio of bacterial chromosomes to plant chromosomes and therefore is dimensionless. **b**, Association of log_10_(bacterial load) with rosette fresh weight. Shaded areas indicate 95% confidence intervals of the association curve; bacterial load was defined as the cumulative abundance of all barcoded isolates that constituted a synthetic community. Pearson’s correlation and the respective *P* value are noted for each synthetic community. For Pathocom, *n* = 170, *n* = 151 for CommenCom, and *n* = 182 for MixedCom.

We hypothesized that the total cumulative load of all inoculated strains, regardless of their taxonomy, should be an important explanatory variable for weight differences among treatments. We based this expectation on the association previously found between prevalence in the field and pathogenicity for similar *Pseudomonas* isolates^11^. Contrary to our hypothesis, we found that while the differences in plant weight between treatments were considerable, the bacterial loads of MixedCom and PathoCom were similar with high probability, as deduced from quantification of barcodes (Fig. 3a). This result implies that plant weight is also a function of bacterial composition and not load per se. In agreement with this inference, the load–weight relationships were found to be treatment dependent, indicating that weight can be better predicted by load within a treatment than by load among treatments (Delta Elpd = −52.9, standard error = 9.4, when comparing the model [weight ~ treatment × log_10_(isolate load) + treatment + log_10_(isolate load) + genotype + experiment + error] to the same model without the interaction factor [treatment × log_10_(isolate load)] using leave-one-out cross validation; Methods).

**Extended Data Fig. 5:**
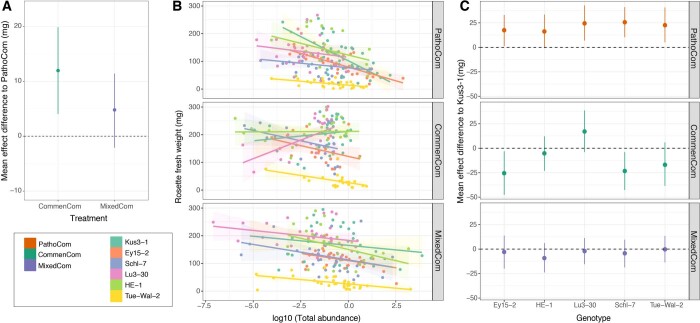
Effect of total load on weight, per treatment and genotype. **a**. Mean slope difference of the three synthetic communities. The slope difference indicates the effect of the treatment on the correlation between weight and isolate load - that is treatment * log_10_(cumulative isolate load) - following the model [weight ~ treatment * log_10_(cumulative isolate load) + treatment + log_10_(cumulative isolate load) + genotype + experiment + error]. PathoCom was selected as a reference. Dots indicate the median estimates, and vertical lines 95% Bayesian credible intervals of the fitted parameter. Related to Fig. 3B. **b**. Correlation of log_10_(cumulative isolate load) with rosette fresh weight, for each of the genotypes within each of the three synthetic communities. Shaded areas indicate 95% confidence intervals of the correlation. Colour codes in the bottom left box, on the right. **c**. Mean slope difference of the six *A. thaliana* genotypes used in this study. The slope difference indicates the effect of the genotype on the correlation between weight and isolate load - that is genotype * log_10_(cumulative isolate load) - following the model [weight ~ genotype * log_10_(cumulative isolate load) + treatment + log_10_(cumulative isolate load) + experiment + error]. Each treatment was analysed individually, thus the model was utilized for each treatment separately. Kus3-1 was randomly selected as a reference. Dots indicate the median estimates, and vertical lines 95% Bayesian credible intervals of the fitted parameter. Related to panel B. n=170 for PathoCom, n=151 for CommenCom, and n=182 for MixedCom. n=77-94 for the six *A. thaliana* genotypes.

We noticed that the regression slope of PathoCom was more negative than the regression slope of CommenCom, suggesting that ATUE5 isolates had a stronger negative impact on weight per bacterial cell than non-ATUE5 isolates (Fig. 3b and Extended Data Fig. 5a; CommenCom mean effect difference to PathoCom: 12.0 mg [4.4,19.5] at 95% credible interval of the parameter log_10_(isolate load) × treatment, based on Extended Data Fig. 5a). From the reciprocal angle, that of the host, it can be seen that plants were less tolerant to ATUE5 isolates than to non-ATUE5 isolates. MixedCom presented a regression slope between the two exclusive synthetic communities, implying that the impact on plant growth resulted from both groups, ATUE5 and non-ATUE5, with the MixedCom mean effect difference to PathoCom being 4.8 mg (based on Extended Data Fig. 5a, [−1.6, 11.8] 95% Bayesian credible interval of the parameter log_10_(isolate load) × treatment). Lastly, we observed differential regression slopes between the host genotypes, particularly among PathoCom- and CommenCom-infected hosts, revealing different levels of tolerance to the same groups of *Pseudomonas* isolates (Extended Data Fig. 5b,c).

**Extended Data Fig. 6:**
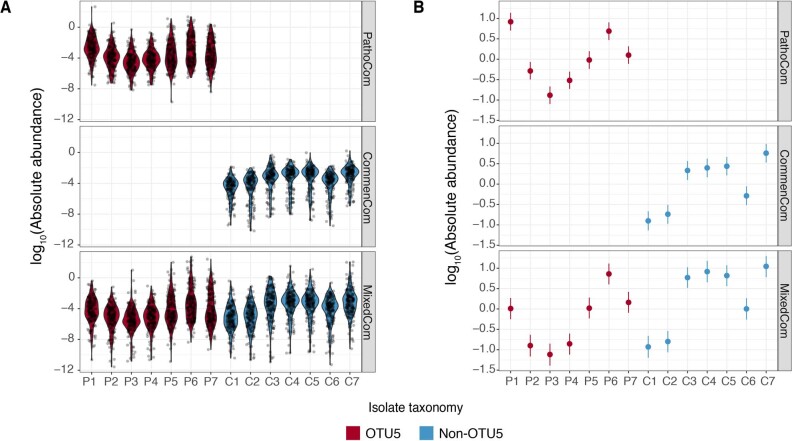
Load of each isolate in plants infected with PathoCom, CommenCom or MixedCom, 12 days post infection. **a**. Raw data. **b**. The estimated abundance of each isolate, derived from the model [log10(isolate load) ~ isolate]. Each treatment was analysed separately, thus the model was used for the subset of each treatment. Dots indicate the median estimates, and vertical lines 95% Bayesian credible intervals of the fitted parameter. n=170 for PathoCom, n=151 for CommenCom, and n=182 for MixedCom.

Although these results suggest general ATUE5 and non-ATUE5 effects, they may still be due to a few dominant strains that outcompeted the others. For example, high competition in the early phases of plant colonization may lead to later exclusion of a subset of strains. In such a scenario, these latter strains would not become established in the plant and would therefore not be particularly relevant. In contrast, we found that in all three synthetic communities, each strain had robustly colonized the plants at the end of the experiment (Extended Data Fig. 6a), confirming that the observed weight differences in host plants are compatible with effects of entire communities. As expected, some strains were more abundant than others, although there was no individual dominant strain in any of the communities (Extended Data Fig. 6b).

We have described two general differences between pathogenic and commensal *Pseudomonas*: (1) on average, pathogens have a greater impact per a given load on plant growth than commensals do and (2) pathogens can reach higher titre in *A. thaliana* leaves than commensals can. Together, this points to dual effects of pathogens on plant health. To explain how commensal non-ATUE5 isolates were able to mitigate the harmful impact of pathogenic ATUE5 in MixedCom, we next addressed the bacterial compositionality in MixedCom-infected hosts.

### Protection by commensal members and pathogen suppression

Given that (1) MixedCom-infected plants grew better than PathoCom-infected plants (Fig. 2, Extended Data Fig. 4a and Supplementary Fig. 4), (2) there was no considerable difference in total load between PathoCom- and MixedCom-infected plants (Fig. 3a) and (3) pathogens were found to cause more damage per cell (Fig. 3b and Extended Data Fig. 5a), we expected commensal members to dominate MixedCom.

**Fig. 4:**
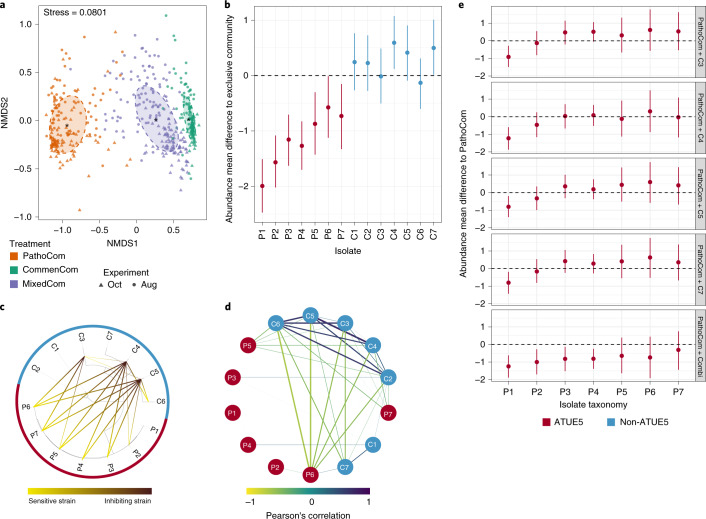
Different in vitro and in planta patterns of inhibition of pathogens by commensals. **a**, NMDS based on Bray–Curtis distances between samples infected with the three synthetic communities across two experiments in August 2018 (Aug) and October 2018 (Oct). The abundance of all 14 barcoded isolates was measured in all communities, including PathoCom and CommenCom, which contained only seven of the 14 isolates, to account for potential cross-contamination and to avoid technical bias. For PathoCom, *n* = 170, *n* = 151 for CommenCom and *n* = 182 for MixedCom. **b**, Abundance change of the 14 barcoded isolates in MixedCom when compared with their exclusive community in infected plants (that is, PathoCom for ATUE5 and CommenCom for non-ATUE5). Abundance mean difference was estimated with the model [log_10_(isolate load) ~ treatment × experiment + treatment + experiment + error] for each individual strain. Thus, the treatment coefficient was estimated per isolate. Dots indicate the median estimates, and vertical lines represent 95% Bayesian credible intervals of the fitted parameter. **c**, Taxonomic representation of the 14 barcoded isolates tested in vitro for directional interactions. Ring colours indicate the bacterial isolate classification, ATUE5 or non-ATUE5. Directional inhibitory interactions are indicated from yellow to black. The experiments were repeated three times with two technical replicates. Only inhibitions observed in at least two independent experiments and in both technical replicates were considered. **d**, Correlation network of relative abundances of all 14 barcoded isolates in MixedCom-infected plants. Strengths of negative and positive correlations are indicated from yellow to purple. Boldness of lines also indicates the strength of correlation, with all correlations >|± 0.2| shown. Node colours indicate the bacterial isolate classification, ATUE5 or non-ATUE5. **e**, In planta abundance change of the seven ATUE5 isolates in non-ATUE5 inclusive treatments in comparison with PathoCom. Abundance mean difference was estimated with the model [log_10_(isolate load) ~ treatment × experiment + treatment + experiment + error] for each individual strain. Thus, the treatment coefficient was estimated per isolate. Dots indicate the median estimates, and vertical lines represent 95% Bayesian credible intervals of the fitted parameter. ‘Combi’ indicates combination of the isolates C3,C4,C5 and C7, and *n* = 23.

**Extended Data Fig. 7:**
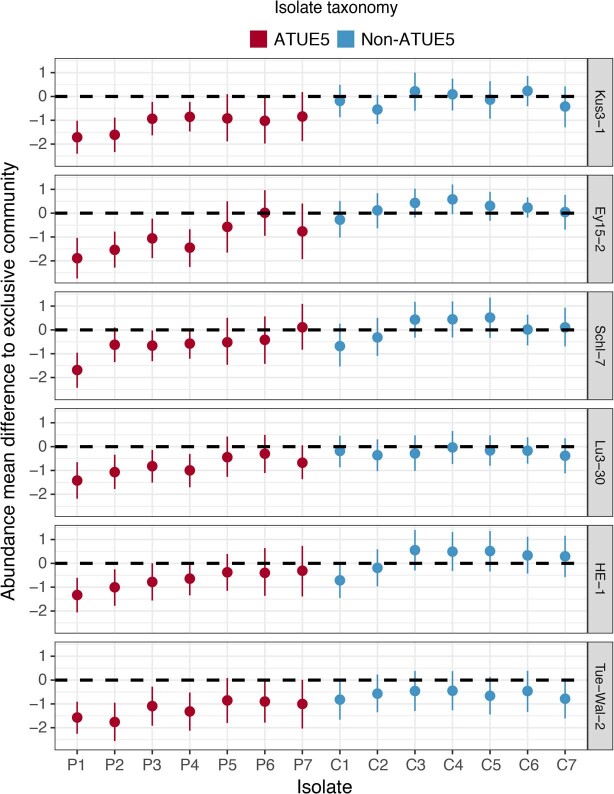
Effect of host genotype on abundance changes of the 14 barcoded isolates. MixedCom compared to the exclusive community (that is, PathoCom for ATUE5 and CommenCom for non-ATUE5). Abundance effect mean differences were estimated with the model [log_10_(isolate load) ~ genotype * treatment + genotype + treatment + experiment + error] for each individual strain. Thus, the genotype * treatment coefficient was estimated per each barcoded isolate. Dots indicate median estimates, and vertical lines 95% Bayesian credible intervals of the fitted parameter.

Consistent with our expectations, the composition of MixedCom was more similar to CommenCom than PathoCom (Fig. 4a). We then analysed the change in bacterial abundance due to the mixture of pathogens and commensals at the isolate level. We compared the absolute abundance of each isolate among the treatments: pathogenic isolates were compared between PathoCom and MixedCom, and commensals between CommenCom and MixedCom. In general, the abundance of pathogens was substantially lower in MixedCom, while the abundance of commensals was either similar or slightly higher in MixedCom (Fig. 4b). Thus, the mixture of pathogens and commensals led to pathogen suppression, while commensal load was largely unchanged in MixedCom compared with CommenCom. Therefore, non-ATUE5 isolates appear to be more competitive in the MixedCom context than ATUE5 isolates. The abundance change of each isolate in the presence of additional community members was similar among the host genotypes, implying that commensal–pathogen interactions were mostly conserved (Extended Data Fig. 7 and Supplementary Table 4). We therefore tested for direct, host-independent interactions among isolates with an in vitro growth-inhibition assay (Methods).

Each of the 14 isolates was examined for growth inhibition against all other isolates, covering all possible combinations of binary interactions. In total, three strains out of the 14 had inhibitory activity; all were non-ATUE5 (Fig. 4c). Specifically, C4 and C5 showed the same pattern: both inhibited all pathogenic isolates but P1, and both inhibited the same two commensals, C6 and weakly C3. C3 inhibited three ATUE5 isolates: P5, P6 and P7. In summary, the in vitro assay provides evidence that among the tested *Pseudomonas* isolates, direct inhibition was a trait unique to commensals, and susceptible bacteria were primarily pathogens. This supports the notion that ATUE5 and non-ATUE5 isolates employ divergent competition strategies, or that if they use the same mechanism, they differ in the effectiveness of such a mechanism.

**Extended Data Fig. 8:**
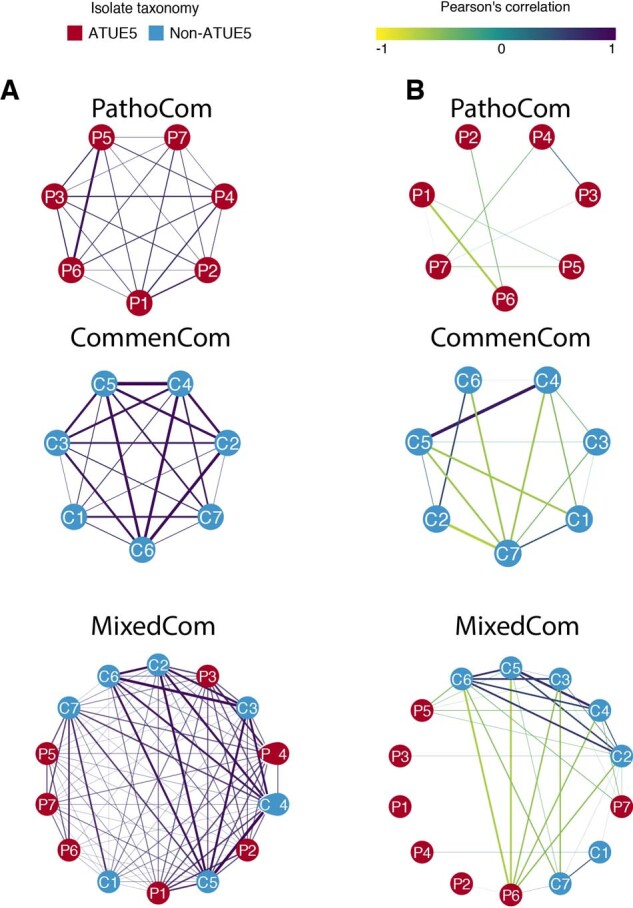
Correlation networks of barcoded bacteria. **a**. Correlation networks of absolute abundance in PathoCom, CommenCom and MixedCom. **b**. Correlation networks of relative abundance in PathoCom and CommenCom. Strengths of negative and positive correlations are indicated from yellow to purple. Boldness of lines is related to the strength of correlation, and only correlations > |±0.2| are shown. Node colours indicate isolate classification as ATUE5 or non-ATUE5.

The in vitro results recapitulated the general trend of pathogen inhibition found among treatments in planta. Nevertheless, we observed major discrepancies between the two assays. First, P1 was not inhibited by any isolate in the host-free assay (Fig. 4c), though it was the most inhibited member in planta among the communities (Fig. 4b). Second, no commensal isolate was inhibited in planta among communities (Fig. 4b), while two commensals, C3 and C6, were inhibited in vitro (Fig. 4c). Both observations are compatible with an effect of the host on microbe–microbe interactions. To explore such effects, we analysed all pairwise microbe–microbe abundance correlations within MixedCom-infected hosts. When we used absolute abundances, all pairwise correlations were positive, also in CommenCom and PathoCom (Extended Data Fig. 8a), consistent with there being a positive correlation between absolute abundance of individual isolates and total abundance of the entire community (Supplementary Fig. 7), that is, no isolate was less abundant in highly colonized plants than in sparsely colonized plants. This indicates that there does not seem to be active killing of competitors in planta in the CommenCom, which is probably not surprising. With relative abundances, however, a clear pattern emerged with a cluster of commensals that were positively correlated, possibly reflecting mutual growth promotion, and several commensal strains being negatively correlated with both P6 and C7, possibly reflecting unidirectional growth inhibition (Fig. 4d). We did not observe the same correlations within CommenCom among commensals and within PathoCom among pathogens as we did for either subgroup in MixedCom, reflecting higher-order interactions. Thus, interactions among pathogens were constrained by the presence of commensals and vice versa (Extended Data Fig. 8b).

The in planta patterns measured in complex communities did not fully recapitulate what we had observed in vitro with pairwise interactions. We therefore investigated individual commensal isolates for their ability to suppress pathogens in planta and also tested the entourage effect. We focused on the three commensals C3, C4 and C5, which had directly inhibited pathogens in vitro, and as a control C7, which had not shown any inhibition activity in vitro. We infected plants with mixtures of PathoCom and each of the four individual commensals and also with PathoCom mixed with all four commensals. Because pathogen inhibition seemed to be independent of the host genotype, we arbitrarily chose HE-1. Regardless of the commensal isolate, only P1 was suppressed with high probability in all commensal-including treatments (Fig. 4e), with P2, P3 and P4 being substantially inhibited only by the mixture of all four commensals. Together with the lack of meaningful differences between individual commensals, this indicates that pathogen inhibition is either a function of commensal dose or a result of interaction among commensals.

An important finding was that four commensal strains had much more similar inhibitory activity in planta than in vitro and that the combined action was greater than the individual effects. Together, this suggested that the host contributes to the observed interactions between commensal and pathogenic *Pseudomonas* isolates. To begin to investigate this possibility, we next studied potential host immune responses with RNA sequencing.

### Defensive response elicited by non-ATUE5

For the RNA-sequencing experiment, we treated plants of the genotype Lu3-30 with the three synthetic communities and also used a bacteria-free control treatment. We sampled the treated plants 3 DPI and 4 DPI, thus increasing the ability to pinpoint differentially expressed genes (DEGs) between treatments that are not highly time specific. Exploratory analysis indicated that the two time points behaved similarly, and they were combined for further in-depth analysis.

**Fig. 5:**
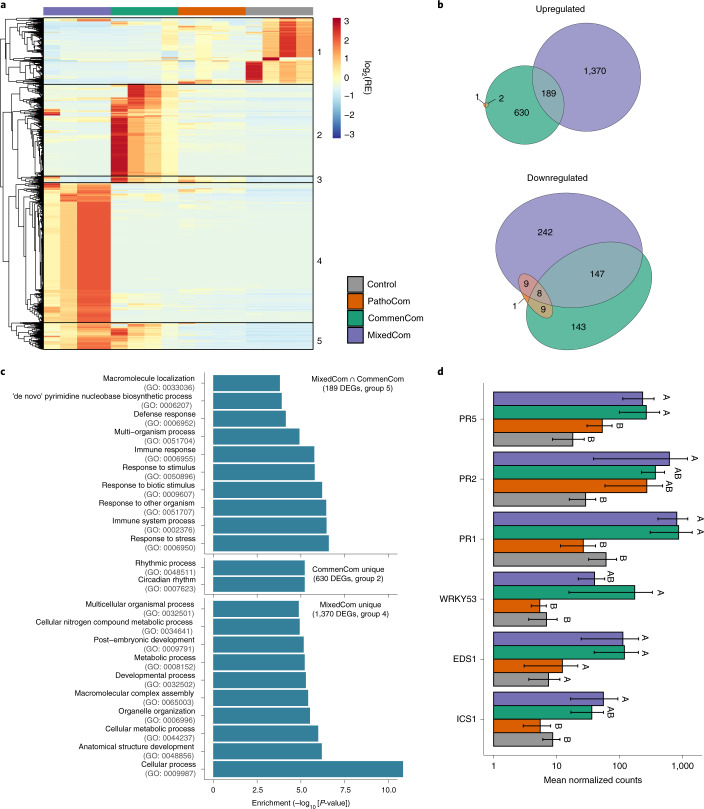
Only commensal members elicit a strong host-defensive response. **a**, Relative expression (RE) pattern of 2,727 DEGs found in at least one of the comparisons of CommenCom, PathoCom and MixedCom with Control. DEGs were hierarchically clustered. **b**, Euler diagram of DEGs in PathoCom-, CommenCom- and MixedCom-treated plants compared with Control (log2[fold change] >|± 1|; false discovery rate (FDR) <0.05; two-tailed Student’s *t*-test followed by Benjamini–Hochberg correction). **c**, Overrepresented GO terms in upregulated DEG subsets: CommenCom and MixedCom intersection (189 DEGs), CommenCom unique (630 DEGs) and MixedCom unique (1,370 DEGs). Only the top ten non-redundant GO terms are presented; for the full lists of overrepresented GO terms and expression data, see Supplementary Table 5, Supplementary Table 6 and Supplementary Data 1. **d**, Expression values of six defence marker genes. Mean ± standard error of the mean (SEM). Groups sharing the same letter are not significantly different (Tukey-adjusted, *P* >0.05); *n* = 4.

**Extended Data Fig. 9:**
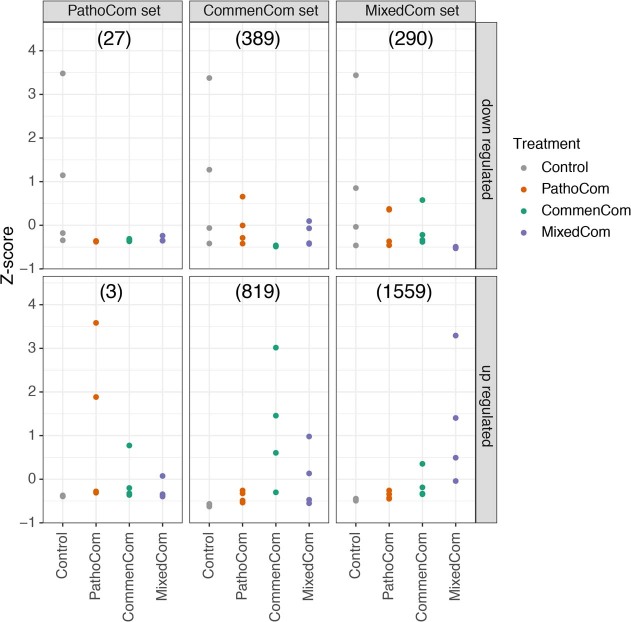
Comparison of PathoCom, CommenCom and MixedCom DEGs across treatments. The average z-score is presented for each sample. Downregulated and upregulated DEGs were analysed separately. Number of DEGs in each category in brackets. n=4.

We first looked at DEGs in a comparison between infected plants and control (Supplementary Table 5); with PathoCom, there were only 14 DEGs; with CommenCom, there were 1,112 DEGs; and with MixedCom, there were 1,949 DEGs, suggesting that the CommenCom isolates, which are also present in the MixedCom, elicited a stronger host response than the PathoCom members. Furthermore, the high number of DEGs in MixedCom, higher than both PathoCom and CommenCom together, suggested a synergistic response derived from inclusion of both PathoCom and CommenCom members. Alternatively, this could also be a consequence of the higher initial inoculum in the 14-member MixedCom than either the 7-member PathoCom or 7-member CommenCom, or a combination of the two effects (Fig. 5a,b and Extended Data Fig. 9). The genes induced by the MixedCom fell into two classes: Group 5 (Fig. 5a,b) was also induced, albeit more weakly, by the CommenCom but not by the PathoCom. This group was overrepresented for non-redundant gene ontology (GO) categories linked to defence (Fig. 5c) and most likely explains the protective effects of commensals in the MixedCom. Specifically, among the top ten enriched GO categories in the shared MixedCom and CommenCom set, eight relate to immune response or response to another organism (‘defence response’, ‘multi-organism process’, ‘immune response’, ‘response to stimulus’, ‘response to biotic stimulus’, ‘response to other organism’, ‘immune system process’, ‘response to stress’; Fig. 5c).

Group 4 was only induced in MixedCom, either indicating synergism between commensals and pathogens or reflecting a consequence of the higher initial inoculum. This group included a small number of redundant GO categories indicative of defence, such as ‘salicylic acid mediated signalling pathway’, ‘multi-organism process’, ‘response to other organism’ and ‘response to biotic stimulus’ (Supplementary Table 6). Moreover, the MixedCom response cannot simply be explained by synergistic effects or commensals suppressing pathogen effects because there was a prominent class, Group 2, which included genes that were induced in the CommenCom but to a much lesser extent in the PathoCom or MixedCom. From their annotation, it was unclear how they can be linked to infection (Fig. 5c). About 500 genes (Group 1) that were downregulated by all bacterial communities are unlikely to contain candidates for commensal protection (Fig. 5a).

Cumulatively, these results imply that the CommenCom members elicited a defensive response in the host regardless of PathoCom members, while the mixture of both led to additional responses. To better understand if selective suppression of ATUE5 in MixedCom infections may have resulted from the recognition of both non-ATUE5 and ATUE5 (reflected by a unique MixedCom set of DEGs) or solely non-ATUE5 (a set of DEGs shared by MixedCom and CommenCom), we examined the expression of key genes related to the salicylic acid pathway and downstream immune responses. Activation of the salicylic acid pathway was previously related to increased fitness of *A. thaliana* in the presence of wild bacterial pathogens, a phenomenon which was attributed to an increased systemic acquired resistance^32^.

We observed a general trend of higher expression in MixedCom- and CommenCom-infected hosts for several such genes (Fig. 5d). Examples are *PR1* and *PR5*, marker genes for systemic acquired resistance and resistance execution. Therefore, according to the marker genes we tested, non-ATUE5 elicited a defensive response in the host, regardless of ATUE5 presence.

We conclude that the expression profiles of non-ATUE5-infected Lu3-30 plants point to an increased defensive status, supporting our hypothesis regarding host-mediated ATUE5 suppression. We note that ATUE5 suppression was not associated with full plant protection and thus control-like weight levels in all plant genotypes. One accession, Ey15-2, was only partially protected in the MixedCom (Fig. 2), despite levels of pathogen inhibition being not very different from other host genotypes (Extended Data Fig. 7).

### Lack of protection explained by a single pathogenic isolate

The fact that Ey15-2 was only partially protected by MixedCom (Fig. 2) underlines the importance of the host genotype in plant–microbe–microbe interactions, apparently reflecting the dynamics between microbes and plants in wild populations. We therefore wanted to reveal the cause for this differential interaction.

**Fig. 6:**
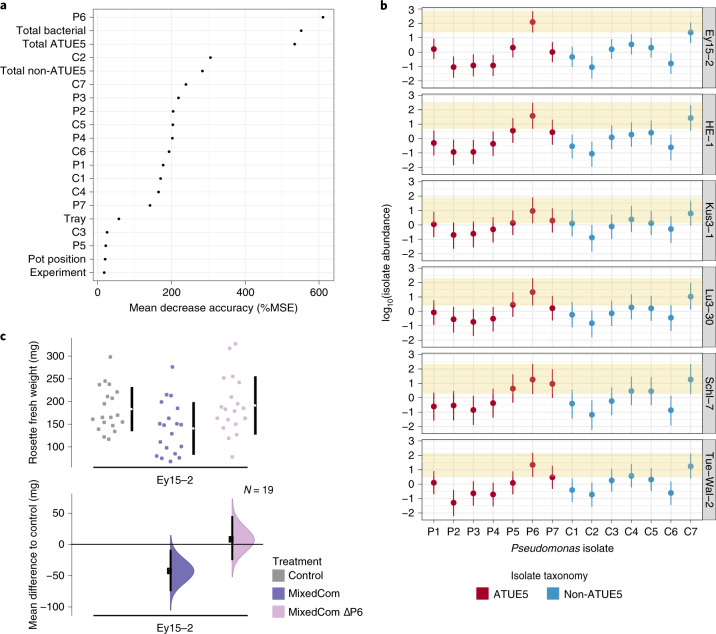
The effect of isolate P6 on weight in MixedCom-infected hosts and particularly on accession Ey15-2. **a**, Relative importance (mean decrease accuracy; ‘MSE’) of 20 examined variables in weight prediction of MixedCom-infected hosts as determined by Random Forest analysis. The best predictor was the abundance of isolate P6. ‘Total bacterial’, ‘Total ATUE5’ and ‘Total non-ATUE5’ indicate the cumulative abundances of the 14 isolates, seven ATUE5 isolates and seven non-ATUE5 isolates, respectively. **b**, Abundance of P6 compared with the other 13 barcoded isolates in MixedCom-infected hosts across the six *A. thaliana* genotypes used in this study. Dots indicate the median estimates, and vertical lines represent 95% Bayesian credible intervals of the fitted parameter, following the model [log_10_(isolate load) ~ isolate × experiment + isolate + experiment + error]. Each genotype was analysed individually, thus the model was utilized for each genotype separately. The shaded area denotes the 95% Bayesian credible intervals for the isolate P6. **c**, Fresh rosette weight of Ey15-2 plants treated with Control, MixedCom and MixedCom without P6 (MixedCom ΔP6). Fresh rosette weight was measured 12 DPI. The top panel presents the raw data, with the breaks in the vertical black lines denoting the mean value of each group, and the vertical lines indicating standard deviation. The lower panel presents the mean difference to control, plotted as bootstrap sampling^55,56^, indicating the distribution of effect size that is compatible with the data. The 95% confidence intervals are indicated by the black vertical bars, and *n* = 19.

**Extended Data Fig. 10:**
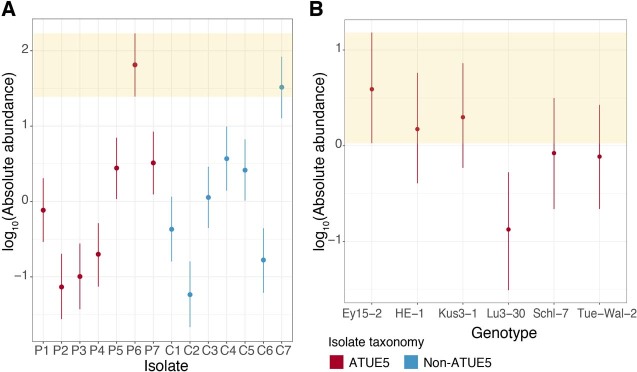
The abundance of P6 in MixedCom-infected hosts. **a**. Abundance of P6 compared with the other 13 barcoded bacteria in MixedCom-infected hosts, for all host genotypes. Dots indicate the median estimates, and vertical lines 95% Bayesian credible intervals of the fitted parameter, following the model [log_10_(isolate load) ~ isolate * experiment + isolate + experiment + error]. Shaded area denotes the 95% Bayesian credible interval of the isolate P6. **b**. The abundance of P6 in MixedCom-infected hosts, compared between the six *A. thaliana* genotypes used. Dots indicate the median estimates, and vertical lines 95% Bayesian credible intervals of the fitted parameter, following the model [log_10_(isolate load) ~ genotype * experiment + genotype + experiment + error]. Shaded area denotes the 95% Bayesian credible interval of the host genotype Ey15-2.

Our first aim was to rank compositional variables in MixedCom according to their impact on plant weight, regardless of host genotype. Next, we asked whether any of the top-ranked variables could explain the lack of protection in Ey15-2. With Random Forest analysis, we estimated the weight-predictive power of all individual isolates in MixedCom and three cumulative variables: total bacterial abundance, total ATUE5 abundance and total non-ATUE5 abundance. We found that the best weight-predictive variable was the abundance of pathogenic isolate P6, followed by total bacterial load and total ATUE5 load, which were probably confounded by the abundance of P6 (Fig. 6a). In agreement, P6 was the dominant ATUE5 in MixedCom (Fig. 6b and Extended Data Fig. 10a). We thus hypothesized that the residual pathogenicity in MixedCom-infected Ey15-2 was caused by P6. Although P6 grew best in Ey15-2, the difference to most other genotypes was unlikely to be important (Extended Data Fig. 10b). However, P6 was particularly dominant in Ey15-2 (Fig. 6b).

Given that pathogen load in Ey15-2 was driven to a substantial extent by P6, we assumed that this isolate had a stronger impact on the weight of Ey15-2 than on other accessions. We experimentally validated that removal of P6 restored protection when Ey15-2 was infected with the MixedCom (Fig. 6c). To confirm that restored protection was due to the interaction of commensals with the five other pathogenic isolates (P1–P5), rather than simply removal of P6, we also treated Ey15-2 with PathoCom only, but not P6. The removal of P6 did not diminish the negative weight impact of PathoCom (P1–P5, Supplementary Fig. 8), implying that it was indeed the interaction between commensals with five out of six pathogenic isolates that mitigated the harmful effect of pathogens in Ey15-2 plants.

## Discussion

We have aimed to understand how complex interactions between closely related *Pseudomonas* strains affect plant health, considering host–microbe, microbe–microbe and host–microbe–microbe relationships. Not surprisingly, we found that genetics mattered at all levels: membership of *Pseudomonas* strain in either a commensal or pathogenic clade; genetic variation within each *Pseudomonas* clade; and genetic diversity among *A. thaliana* host strains. Commensal *Pseudomonas* can protect *A. thaliana* from the effects of pathogenic *Pseudomonas* by reducing their proliferation within the plant. However, although this was a general phenomenon, one *A. thaliana* genotype was only partially protected, and this was due to this genotype being particularly susceptible to a specific *Pseudomonas* pathogen. Together, this demonstrates how the host environment can affect microbe–microbe interactions.

The importance of protective interactions for plant health has been demonstrated in both agricultural and wild contexts^3,19,33^. Our results reveal the extreme specificity of these interactions, with closely related pathogenic isolates interacting differently with protective strains. We found that upon co-infection with a mixture of pathogens and commensals, pathogens were preferentially suppressed. Perhaps our most important finding was that different plant responses were induced by the three different synthetic communities, comprising either only commensals, only pathogens or both. Specifically, commensals but not pathogens induced a transcriptome signature of defence, and these were further enhanced in the presence of pathogens. In addition, there were sets of genes that were no longer induced when plants were infected by the mixed community rather than only commensals and sets of genes specifically induced only by the mixed community. This suggests not only that microbe–microbe interactions alter the plant response but also that these altered plant responses are causal for the differential proliferation of commensals and pathogens in plants affected with mixed communities. Synergistic host responses have been demonstrated, for example, in *Medicago truncatula*, following infections with two classes of symbionts, rhizobia and mycorrhizal fungi^34^. These findings support the hypothesis that the complex interplay between the plant immune system and the microbiota goes beyond individual plant–pathogen interactions, eventually leading to microbial homoeostasis^35^. The exact mechanism behind the synergistic effect we describe must still be investigated, though known cases of host-dependent protective interactions provide plausible explanations. For example, early exposure to harmless rhizosphere microbes can prime the plant to suppress at a later time point a broad range of pathogens even in distal tissues, a phenomenon known as induced systemic resistance^28^.

Another strength of our study is that we used naturally co-occurring biological material, namely strains of the *A. thaliana* host and *Pseudomonas* bacteria that had been isolated from a single geographic area. Our results potentially help to explain why the *Pseudomonas* pathogens used here, which are lethal in mono-associations, seem to cause only limited disease in the field^11^, namely their effects being modified by other microbes, including other *Pseudomonas* strains.

A limitation of the current study was that we examined only a few commensal isolates and tested them mostly in complex mixtures. A next logical step will be to test the protective effects of individual commensal *Pseudomonas* strains from the local Tübingen collection to explore (1) how common protection by commensal *Pseudomonas* is, (2) how much it depends on the genotype of the pathogen and (3) what the genes are that support protection.

We used pathogenic isolates that share over 99% of their 16S rDNA signature and are highly similar in their core genome. Nonetheless, we found functional differences relating to both host–microbe and microbe–microbe interactions, exemplified by an individual pathogenic *Pseudomonas* isolate that both dominated the mixed synthetic communities and that caused a lack of protection in one host genotype. In agreement, Karasov and colleagues^11^ had already found that members of this clade of *Pseudomonas* differ substantially in their ability to cause disease in mono-associations.

Friedman and colleagues^36^ accurately predicted microbial community structures in the form of trios based on information about pairwise interactions. How easily, however, higher-order communities can be predicted from pairwise interactions remains to be seen, although recent statistical advances are promising^37,38^. The genome-barcoding method we developed allows strain-level tracking and thus can be implemented to understand multi-strain community assembly. However, in its current format, it is limited to low-throughput studies, mainly due to the cumbersome cloning and transformation serial process. An alternative is presented by high-throughput experiments that combine whole-genome sequencing with statistical reconstitution of known haplotypes^26,39^ and which could be employed to study the dynamics of more complex communities.

More and more studies are revealing effects that can be found only by the ensemble of relationships. For example, inflammatory bowel disease^40^ has been linked to changes in microbial community structure rather than to an individual microbe. Another example is provided by plant beneficial consortia, in which only microbial mixtures—but not any single strain—triggered pathogen suppression^41,42^. Further advancements in understanding the effects of plant–microbe–microbe interactions on plant health may improve agriculture practices, allowing the development of more sustainable plant protection methods^43–45^.

## Methods

### Plant material

*Arabidopsis thaliana* accessions HE-1, Lu3-30, Kus3-1, Schl-7, Ey15-2 and Tue-Wal2, all originally collected from around Tübingen, Germany^28^ (Supplementary Table 7), were used in this study. Seeds were sterilized by overnight incubation at −80 °C, followed by ethanol washes (shake seeds for 5–15 min in solution containing 75% EtOH and 0.5% Triton-X-100 and then wash seeds with 95% EtOH and let them dry in a laminar flow hood). Seeds were stratified in the dark at 4 °C for 6–8 days before planting in potting soil (CLT Topferde, einheitserde.de). Plants were grown in 60-pot trays (Herkuplast Kubern), in which compatible mesh-net pot baskets were inserted to allow for subsequent relocation of the pots. Plants were grown in short days (8 h of light) at 23 °C with light intensity of 125–175 μmol m^−2^ s^−^^1^ and relative humidity of 65%.

### Barcoding *Pseudomonas* isolates

Excluding the *E. coli* strains that were used for cloning, all 14 bacterial isolates used in this study were classified as *Pseudomonas* and collected from two locations around Tübingen^11^ (Supplementary Table 1). The procedure of genome barcoding of the 14 bacterial isolates included: preparation of random barcodes, cloning of barcodes into pUC18R6KT-mini-Tn7T-Km and co-transformation of bacteria with the recombinant pUC18R6KT-mini-Tn7T-Km derivative and pTNS2 helper, both from^29^. Preparation of barcodes and the flanking priming sites was done by double stranding two overlapping single strand oligonucleotides: one that contains restriction sites, a universal priming site, 16 random nucleotides and an overlapping region (Bar1) and another that contains the reverse complement overlapping region, the second universal priming site and restriction sites (Bar2, Supplementary Fig. 9); oligonucleotide details are provided in Supplementary Table 8. The two overlapping single strand oligonucleotides were mixed in an equimolar fashion (5 ng each, 2 μl in total) together with 0.2 μl Q5 high-fidelity DNA polymerase (New England Biolabs), 1× Q5 5× reaction buffer and 225 μM dNTP in a total reaction volume of 20 μl. The mixture was made double-stranded reaction in a thermocycler (Bio-Rad Laboratories) with the following programme: 95 °C for 40 s, 55 °C for 60 s and 72 °C for 3 min. The resulting product was cloned into pUC18R6KT-mini-Tn7T-Km using restriction enzymes XhoI and SacI and ligation with T4 DNA ligase (Thermo Fisher Scientific) and transformed into Pir1 competent *E. coli* (Thermo Fisher Scientific). Bacterial colonies were validated as successful transformants by PCR with primers p1 and p2 (Supplementary Table 8). Positive colonies were grown in LB overnight and then used for subsequent plasmid isolation (GeneJET Plasmid Miniprep Kit, Thermo Fisher Scientific). About 150 pUC18R6KT-mini-Tn7T-Km recombinant plasmids were obtained. Sanger sequencing was conducted on a subset of the plasmids using primer p1 to determine their barcode sequences. Fourteen barcode-positive plasmids were randomly selected and co-transformed with plasmid pTNS2 to genome barcode the selected 14 *Pseudomonas* isolates as described^29^. Briefly, *Pseudomonas* strains were grown overnight in LB, pelleted and washed with 300 mM sucrose solution to create electrocompetent cells and were finally electroporated with the recombinant pUC18R6KT-mini-Tn7T-Km derivatives and pTNS2 in a 1:1 ratio. Transformed *Pseudomonas* isolates were grown on selective LB-agar media with 30 mg ml^−1^ Kanamycin, and colonies were validated by PCR with primers p1 and p2 (Supplementary Table 8 and Supplementary Fig. 1a). Positive colonies were grown overnight in LB with 30 mg ml^−1^ kanamycin, and one portion was stored at −80 °C in 25% glycerol, while the other was used for DNA extraction (Puregene DNA extraction kit, Invitrogen) followed by Sanger sequencing to validate the barcode sequences (Supplementary Table 1).

### Comparison of growth of barcoded and parental isolates

To compare the growth of the 14 barcoded bacteria with their respective parents, both barcoded and parental isolates were grown overnight in LB and 10 mg ml^−1^ nitrofurantoin (an antibiotic to which our *Pseudomonas* strains are resistant), diluted 1:10 in the following morning and grown for an additional 3 h until they entered log phase. Subsequently, bacteria were pelleted at 3,500 g and resuspended in LB to a concentration of OD_600_ = 0.0025 in a 96-well microtitre plate with a transparent, flat bottom (Greiner Bio One). Finally, the plate was incubated in a plate reader at 28 °C while shaking for 10 h (Robot Tecan Infinite M200, Tecan Life Sciences). OD_600_ was measured in 1 h intervals.

### Synthetic community infections and plant sampling

Barcoded isolates were grown overnight in LB and 30 mg ml^−1^ kanamycin, diluted 1:10 the following morning, grown for an additional 3 h until they entered log phase, pelleted at 3,500 g, resuspended in 10 mM MgSO_4_, pelleted again at 3,500 g and resuspended in 10 mM MgSO_4_ to a concentration of OD_600_ = 0.2. Barcoded isolates were mixed at a concentration of OD_600_ = 0.0143 per isolate. Thus, the total concentration per synthetic community was OD_600_ = (0.0143 × number of isolates), the seven-member PathoCom and CommenCom had total concentrations of OD_600_ = ~0.1 and the 14-member MixedCom a total concentration of OD_600_ = ~0.2. Per plant, 2.5 ml of inoculum was prepared. Control treatment was sterile 10 mM MgSO_4_ solution. Heat-killed PathoCom was prepared by incubating the PathoCom at 100 °C for 2 h. All solutions with synthetic communities were stored at 4 °C overnight, and infections were conducted the following morning.

Leaves of soil-grown plants were spray infected 21 days after sowing with an airbrush (BADGER 250-1, Badger Air-Brush Co.), with each plant sprayed on both the abaxial and adaxial side for about 1.5 s each. Plants of the same treatment group were placed together in 60-pot trays (Herkuplast Kubern) in which compatible mesh-net pot baskets were pre-inserted to allow for subsequent relocation of the pots. After the treatment, the transportable pots were reshuffled in new 60-pots trays to form a full randomized block design, thus each tray contained plants from all treatments in equal amounts. The randomized trays were covered with a transparent lid to increase humidity (Bigger Greenhouse 60 × 40 cm, Growshop Greenbud). Four DPI, two built-in openings in the lids were opened to allow for better air flow and to limit humidity. Eight DPI, lids were removed. Twelve DPI, the rosettes of all treated plants were detached using sterilized scalpel and tweezers, weighed, washed from epiphytes (sterile distilled water, 70% EtOH with 0.1% Triton-X-100, and then again with sterile distilled water), dried using sterilized paper towels and sampled in 2 ml screw cap tubes prefilled with 1 mm Garnet sharp particles. Tubes with the sampled plants were flash frozen in liquid N_2_ and stored at −80 °C.

For infections on MS agar, the PathoCom was diluted 1:10 to OD_600_ = 0.01, and 12- to 14-day-old plants were infected by dripping 200 μl of the corresponding inoculum onto whole rosettes.

### DNA extraction, barcode PCR and qPCR

DNA was extracted from frozen samples as described^11^. Briefly, samples were subjected to bead beating in the presence of 1.5% sodium dodecyl sulfate and 1 mm Garnet sharp particles, followed by sodium dodecyl sulfate cleanup with 1/3 volume 5 M potassium acetate and then SPRI beads. The DNA extract was subject to a two-step PCR procedure. The first PCR step amplified the genome-integrated barcodes and added short overhangs, using the primer p3 and the primers p4–p9. The latter are different versions of one primer with frameshifting nucleotides (Supplementary Table 8), allowing for better clustering on the Illumina flow cells and thus sequencing quality as described^46^. Each primer frameshift version was used for a different PCR plate with 96 samples. The second PCR step primed the overhangs to Illumina adaptors for subsequent sequencing using standard Illumina TruSeq primer sequences. Unique tagging of PCR samples was accomplished by using 96 indexing primers combined with the six combinations of frameshift primers in the first PCR^46^, allowing multiplexing of up to 576 samples in one Illumina lane. The first PCR was done in 25 μl reactions containing 0.125 μl Taq I DNA polymerase (Thermo Fisher Scientific), 1× Taq1 10× reaction buffer, 0.08 μM each of forward and reverse primer, 225 μM dNTP and 1.5 μl of the template DNA. The first PCR was run for 94 °C for 5 min followed by 10 cycles of 94 °C for 30 s, 55 °C for 30 s, 72 °C for 1 min and a final 72 °C for 5 min. Five μl of the first PCR product was used in the second PCR with tagged primers including Illumina adaptors in 25 μl containing 0.25 μl Q5 high-fidelity DNA polymerase (New England Biolabs), 1× Q5 5× reaction buffer, 0.08 μM forward and 0.16 μM of reverse (tagging) primer and 200 μM dNTP. The final PCR products were cleaned twice using SPRI beads in a 1:1 bead-to-sample ratio and eluted in 15 μl. Samples were combined into one library in an equimolar fashion. Final libraries were cleaned twice using SPRI beads in a 0.6:1 bead-to-sample ratio to clean the primers from the product and were finally eluted in half of their original volume. Samples were sequenced by a MiSeq instrument (Illumina), using a 50 bp single-end kit.

To estimate the ratio of barcoded *Pseudomonas* to plant chromosomes, two qPCR reactions were conducted, one specific to the barcodes and the other targeting sequences from the *A. thaliana* single-copy gene *GIGANTEA*. For barcode-specific qPCR, primers p10 and p11 were used, and for plant-specific qPCR, primers p12 and p13 (Supplementary Table 8). qPCR reactions were done in 10 μl reactions containing ×1 Maxima SYBR green qPCR master mix ×2, 0.08 μM each of forward and reverse primer and 1 μl of template DNA. qPCR reactions were run on a BioRad CFX384 Real-Time PCR System instrument with the following protocol: 94 °C for 2 min, 94 °C for 15 s and 60 °C for 1 min. Reactions were done in triplicates.

### Host-associated microbial PCR library construction

We used a two-step amplification method, host-associated microbial PCR (hamPCR), to determine microbial composition relative to the plant^30^. Briefly, the host and microbe were differentially tagged by a two-cycle PCR (HM-tagging), followed by simultaneous amplification of the barcoded DNA molecules. For the *A. thaliana* hosts, we used primer pairs to target the single-copy *GIGANTEA* gene. For bacteria, we targeted the V4 region of 16S rDNA (515F–799R) while suppressing non-targeting amplification of mitochondrial and chloroplast DNA using peptide nucleic acid clamps^30^. Unique tagging of PCR samples was accomplished with 96 indexing primers^46^. The library of pooled samples was sequenced on an Illumina HiSeq3000 instrument.

### In vitro directional suppression assay

Pairwise in vitro interactions among the 14 barcoded isolates were tested as described^47^ with modifications for *Pseudomonas*. Briefly, isolates were grown overnight in LB with 30 mg ml^−1^ kanamycin, diluted 1:10 the following morning and regrown. One portion was taken from each isolate after 3 h when entering the log phase, diluted to a final concentration of OD_600_ = 0.001 in 15 ml warm LB with 1% agar and immediately poured into a square plate to form a uniform layer containing the test strain. Another portion was pelleted at 3,500 g, washed from residual LB in 10 mM MgSO_4_, pelleted again at 3,500 g and resuspended in half of the original volume of 10 mM MgSO_4_. Roughly 1 μl of each strain was printed onto the solidified agar layer containing the strain to be tested for sensitivity. Inhibition was estimated after 1–2 days incubation at 28 °C by measuring halo sizes^47^.

### RNA sequencing

Plants from the genotype Lu3-30 were infected with Control, PathoCom, CommenCom and MixedCom as described below. Sampling was conducted 3 DPI and 4 DPI, with two replicates per treatment and time point. Plants were sampled using sterilized scalpel and tweezers and were immediately placed in 2 ml screw cap tubes prefilled with 1 mm Garnet sharp particles, flash frozen in liquid N_2_ and stored at −80 °C. RNA was extracted from frozen samples as described^48^. Briefly, frozen samples were ground and guanidine hydrochloride buffer was added, followed by phase separation and sediment removal. After adding 96% EtOH, the solution was loaded onto a plasmid DNA extraction column (QIAprep Spin Miniprep Kit, Qiagen) and washed several times before elution of the RNA. mRNA enrichment and sequencing libraries were prepared as previously described^49^. Briefly, mRNA was enriched using NEBNext Poly(A) mRNA Magnetic Isolation Module (New England Biolabs) followed by heat fragmentation. Next, first-strand synthesis (SuperScript II reverse transcriptase, Thermo Fisher Scientific) and second-strand synthesis (DNA polymerase I, New England Biolabs) were carried out, followed by end repair (T4 DNA polymerase, Klenow DNA polymerase and T4 Polynucleotide Kinase; New England Biolabs) and A-tailing (Klenow Fragment, New England Biolabs). Nextera-compatible universal adaptors^50^ were ligated to the products (T4 DNA ligase, New England Biolabs), followed by i5 and i7 PCR (Q5 polymerase, New England Biolabs). Size selection and DNA purification were carried out with SPRI beads. Samples were sequenced on a HiSeq3000 instrument (Illumina) using a 150 bp paired-end kit.

### Map, phylogenetics and isolates abundance in the field

Locations of *A. thaliana* and *Pseudomonas* isolates (Supplementary Tables 1 and 7) were plotted using the ‘ggmap’ function of the ggmap R package^51^. Phylogenetic analysis of the 14 selected *Pseudomonas* isolates was done using core genomes^11^. Maximum-likelihood phylogenies were constructed with RAxML (v.0.6.0) using the GTR + Gamma model^52^, and visualization was done by iTOL^53^. The abundance in the field of the selected isolates was estimated by binning similar isolates using a threshold of nucleotide divergence <0.0001 in the core genome. The mean number of substitutions per site was taken from the estimated branch length for the core genome-based phylogeny calculated by RAxML. Lastly, the number of binned isolates was divided by the total number of isolates^11^.

### Growth analysis of parental and barcoded isolates

Growth of both parental and barcoded isolates was analysed using the function ‘SummarizeGrowthByPlate’ from the Growthcurver R package^54^. The change of barcoded isolates in comparison to their corresponding wild type (‘WT’) in growth rate, carrying capacity and area under the curve was calculated by the model: growth quantity ~ strain type (that is, parent/barcoded).

### Plant weight analysis

All rosette fresh weight analyses and visualizations were generated using the function ‘dabest’ of the dabestr R package^55,56^. In brief, the function presents (1) all raw data points as a swarm plot ordered to display the distribution and (2) the computed effect size after bootstrapping (in comparison to control plants), indicating the mean and 95% confidence interval. Thus, the computed confidence interval denotes the distribution of the effect size that is compatible with the data, reflecting the average change of weight (in mg) in respect to control plants after infection in a given treatment.

### Combining barcode PCR and qPCR to estimate isolate load

All reads from barcode-PCR sequencing were mapped against a custom barcode database (Supplementary Table 1) using the algorithm BWA-MEM^57^ (version 0.7.17-r1188), and a count matrix of all 14 isolates for every plant sample was created. Samples with less than a total of 200 hits were discarded or resequenced (mean = 15,710). Counts were transformed to proportions by dividing the counts of each isolate in the total hits per sample, resulting in a relative abundance matrix.

qPCR results were analysed using the software Bio-Rad CFX Manager (v3.1) with default parameters. Quantification cycle (Cq) values smaller than 32 were discarded, and barcoded bacterial load was determined by the equation ${\mathrm{bacterial}}\,{\mathrm{load}} = \frac{{2.057^{ - (\mathrm{barcode}\,\mathrm{Cq})}}}{{2.027^{ - (\mathrm{GIGANTEA}\,\mathrm{Cq})}}}$. The exponent bases (2.057 and 2.027) were adjusted according to primer efficiency—as determined by a calibration curve derived from a series of dilutions. The relative abundance matrix was factorized by bacterial load (relative abundance multiplied by bacterial load per isolate) to manifest the ratio of bacterial to plant chromosomes per barcoded isolate.

### Variance partitioning of microbial community composition

NMDS analyses used the function ‘metaMDS’ in the R package vegan^58^, adjusting dissimilarity index to Bray–Curtis (method = bray), number of dimensions to 3 (*k* = 3) and maximal iterations to 200 (trymax = 200). PERMANOVA was conducted using the function ‘adonis’, and analysis of similarities was conducted using the function ‘anosim’, also in the R package vegan^58^. Both were adjusted to Bray–Curtis dissimilarity index (method = bray) and 2,000 permutations (permutations = 2,000). Multi-level pairwise comparison using ‘adonis’ was conducted using the function ‘pairwise.adonis2’ in the R package pairwiseAdonis^59^.

### Bacterial profiling with hamPCR

Bacterial profiling with hamPCR used a dedicated computational pipeline^30^. In short, all samples were filtered to remove mismatched sequences from the expected primers. Subsequently, all primer sequences were trimmed, followed by quality filtering and removal of chimeric sequences. Amplicon sequence variant counting and their respective taxonomic classification were done with a combination of VSEARCH^60^ and USEARCH10^61^. Amplicon sequence variant tables were generated and factorized by plant to quantify bacterial load for each sample.

### Regression analysis

In all figures except Fig. 6b and Supplementary Fig. 16, posterior distributions of focal factors were estimated using the function ‘stan_glm’ in the R package rstanarm^62^. In Fig. 6b and Supplementary Fig. 16, ‘lmBF’ followed by the function ‘posterior’ in the R package BayesFactor^63^ was used to assess posterior distributions without a comparison to a control baseline. In Bayesian analyses, default priors were used. For both ‘stan_glm’ and ‘lmBF’, default iteration number was used (four Markov chains of 2,000 iterations each in ‘stan_glm’, and 10,000 iterations in ‘lmBF’ and ‘posterior’). Markov chain Monte Carlo (‘MCMC’) convergence was assessed by the Rhat measure, resulting in values ranging from 0.99 to 1.01. In all figures, we present for each factor of interest the median estimates (that is, the median of the posterior distribution) and the 95% credible intervals, corresponding to the intervals from 2.5% to 97.5% of the posterior distributions. The overlap between a coefficient’s 95% credible interval with a control baseline (zero) or another coefficient’s 95% credible interval was used as an indication for hypothesis testing, that is, the lack of overlap implied a substantial effect. Importantly, we considered the lack of overlap not only to gauge differences between coefficients but also the magnitude of difference, respecting the uncertainty of the population mean and the measured values. The exact model for every analysis is presented in the figure legends and the selected references for comparison.

To compare the effect of individual predictors in a model, the full model was compared to a different model, lacking the predictor of interest (for example, genotype). The comparison was conducted by a leave-one-out cross validation, using the function ‘loo_compare’ in the R package Loo^64^. This Bayesian-based model comparison provides an assessment of the prediction accuracy of a model with and without a specific predictor. We used leave-one-out cross validation because it has been shown to improve model selection in comparison to the common Akaike information criterion and deviance information criterion^64^. An additional advantage of leave-one-out cross validation is the opportunity to obtain approximate standard errors for estimated predictive errors to compare predictive errors between two models. The reported output is the difference in expected log-scaled predictive density (‘Delta Elpd’), as indicated in the text.

### Isolate–isolate interaction network

All pairwise isolate–isolate Pearson correlations were calculated using the function ‘corr’ in the R package Hmisc^65^ and visualized with Cytoscape 3.7.0^66^.

### RNA-sequencing analysis

RNA-sequencing reads were mapped against the *A. thaliana* reference TAIR10 using STAR (v.2.6.0;^67^ with default parameters. A transcript count matrix was calculated using ‘featureCounts’^68^, while restricting counts to exons only. DEGs were identified with DESeq2 (v.1.22.2 (ref. ^69^)), using the model [gene_expression ~ treatment + time_point]. Genes with average counts of less than 5 were excluded from the analysis. Zero counts were converted to 1 to allow for the log conversion in unexpressed genes. Genes with log2FoldChange >|± 1| and FDR <0.05 (two-tailed Student’s *t*-test followed by Benjamini–Hochberg correction) were defined as DEGs. Euler diagrams were created using the function ‘euler’ in the R package eulerr^70^. Statistically overrepresented GO terms were identified using the BiNGO plugin (v3.0.3) for Cytoscape^71^. Summarization and the removal of redundant overrepresented GO terms was done with the web server REVIGO^72^ to extract main trends from the full output by BiNGO (Supplementary Table 6).

### Statistical analysis

Statistical analyses were performed using the R environment version 3.5.1 unless mentioned otherwise. Sample sizes were not predetermined using statistical methods.

### Reporting Summary

Further information on research design is available in the Nature Research Reporting Summary linked to this article.

## Supplementary information

Supplementary Information Supplementary Data 1 legend, Supplementary Tables 1–4 and 6–7, Supplementary Table 5 legend and Supplementary Figs. 1–9.

Reporting Summary.

Peer Review Information.

Supplementary Table Supplementary Table 5.

Supplementary Data Supplementary Data 1.

## Data Availability

RNA-sequencing data have been deposited with the European Nucleotide Archive (ENA) under study accession number PRJEB41069. Raw data of plant weights and the abundance of strains are deposited at https://github.com/orshalevsk/Pseudomonas_SynComs_Athaliana.

## Code availability

Code for statistical analyses and figure production is available at https://github.com/orshalevsk/Pseudomonas_SynComs_Athaliana.
