# Supplementary material for: Commensal Pseudomonas strains facilitate protective response against pathogens in the host plant
Source: Nat Ecol Evol. 2022 Feb 24;6(4):383–96. doi: 10.1038/s41559-022-01673-7 (PMC8986537; doi:10.1038/s41559-022-01673-7)
Supplement: Supplementary file 1 — Supplementary Data 1 legend, Supplementary Tables 1–4 and 6–7, Supplementary Table 5 legend and Supplementary Figs. 1–9. [file 41559_2022_1673_MOESM1_ESM.pdf]

nature portfolio

https://doi.org/10.1038/s41559-022-01673-7

Supplementary information

Commensal *Pseudomonas* strains facilitate protective response against pathogens in the host plant

In the format provided by the authors and unedited

Supplemental Material for

Shalev et al.: Commensal Pseudomonas strains facilitate protective response against pathogens in the host plant

Supplementary Data 1. RNA-seq expression measurements.

Supplementary Table 1. Metadata and barcode sequences for the 14 isolates.

Isolate	Name in this study	ATUE[†]	Type	Location	Barcode	Similar isolates in Karasov et al. (2018) collection*
p4.C9	P1	ATUE5	Pathogen	Eyach	GTATCTGTAAGAAAAA<br>TCGATTTCCAGGC	23
p3.A5	P2	ATUE5	Pathogen	Eyach	GTATGCTGAAAACAAA<br>CTATTTGCTTGGC	115
p3.A9	P3	ATUE5	Pathogen	Eyach	GTATAGTAAAGCACAA<br>GAAATTGTCGGGC	24
p3.B12	P4	ATUE5	Pathogen	Eyach	GTATGCCAAAGATGAA<br>CTACTTGTCTGGC	156
p4.A6	P5	ATUE5	Pathogen	Eyach	GTATGAAGAAGATGAA<br>TCATTTACCTGGC	236
p4.E1	P6	ATUE5	Pathogen	Eyach	GTATCCGCAAACGTAA<br>GCACTTAGACGGC	196
p4.G8	P7	ATUE5	Pathogen	Eyach	GTATAGGTAATAAGAA<br>AGCCTTATACGGC	168
p5.H10	C1	ATUE-<br>NEW	Commensal	Eyach	GTATCGCCAACACGAA<br>CCCGTTAAATGGC	5
p3.G10	C2	ATUE-<br>NEW	Commensal	Eyach	GTATACCCAACACCAA<br>GCCCTTATCGGGC	5
p4.C4	C3	ATUE4	Commensal	Eyach	GTATCTTGAATTCTAA<br>CCGTTTACCCGGC	17
p4.H11	C4	ATUE2	Commensal	Det-2	GTATAGAAAATGAAAA<br>GATGTTCTCTGGC	23
p4.H10	C5	ATUE2	Commensal	Det-2	GTATCCAGAACTGCAA<br>CAGCTTTACAGGC	12
p3.G11	C6	ATUE2	Commensal	Eyach	GTATCACTAACAGGAA<br>CTCTTTCCCTGGC	17
p5.F2	C7	ATUE2	Commensal	Eyach	GTATCACTAAGGATAA<br>TCCGTTTTATGGC	6

[†]OTU classification from Karasov et al. (2018)
*nucleotide sequence divergence < 0.0001 in core genome

Supplementary Table 2. Analysis of batch effects and synthetic community composition. A. Analysis of similarities (ANOSIM) based on Bray-Curtis distances for composition of the 14 barcoded bacteria in treated hosts. The analysis was constrained by the host genotype in each experimental batch (exp) to estimate its effect on the explained variance. **B.** Multilevel pairwise comparison of synthetic community composition for the different *A. thaliana* genotypes, using adonis based on Bray-Curtis distances. Data derived from one representative experiment (October 2018). Statistically significant differences ($P < 0.05$) in bold.

A. ANOSIM

Treatment	R^2	Pr(>f)	Experiment
PathoCom	0.0630	**0.0175**	August 2018
	0.1792	**0.0005**	October 2018
CommenCom	0.0622	0.0615	August 2018
	0.1761	**0.0005**	October 2018
MixedCom	0.0538	**0.0265**	August 2018
	0.0951	**0.0005**	October 2018

B. Pairwise comparison of synthetic community composition

Treatment	R^2	$P_{r(>f)}$	Genotype1	Genotype2
PathoCom	0.1491	0.005	Ey15-2	HE-1
	0.2561	**0.001**	Ey15-2	Lu3-30
	0.1236	**0.01**	Ey15-2	Schl-7
	0.0804	**0.044**	HE-1	Kus3-1
	0.1678	**0.001**	HE-1	Lu3-30
	0.1313	**0.001**	Kus3-1	Lu3-30
	0.0757	**0.049**	Kus3-1	Schl-7
	0.0865	**0.002**	Lu3-30	Schl-7
	0.1986	**0.001**	Lu3-30	Tue-Wal-2
CommenCom	0.0813	**0.035**	Ey15-2	Lu3-30
	0.1285	**0.028**	Ey15-2	Tue-Wal-2
	0.1330	**0.004**	HE-1	Lu3-30
	0.2450	**0.001**	HE-1	Tue-Wal-2
	0.2197	**0.001**	Kus3-1	Lu3-30
	0.1305	**0.005**	Kus3-1	Schl-7
	0.1709	**0.007**	Kus3-1	Tue-Wal-2
	0.4401	**0.001**	Lu3-30	Tue-Wal-2
	0.3788	**0.001**	Schl-7	Tue-Wal-2
MixedCom	0.0825	**0.012**	Ey15-2	Lu3-30
	0.0818	**0.01**	Ey15-2	Tue-Wal-2
	0.0673	**0.027**	HE-1	Tue-Wal-2
	0.1660	**0.001**	Kus3-1	Lu3-30

	0.2283	**0.001**	Lu3-30	Tue-Wal-2

Supplementary Table 3. Model tests. A. Importance of plant genotype, treatment and their interaction (genotype*treatment), estimated by model comparison using leave-one-out cross-validation. Preferred model in bold. B. Two-way ANOVA test for the model [weight ~ genotype * treatment + genotype + treatment + experiment]. Statistically significant relationships ($P < 0.05$) in bold.

A. Model comparisons using leave-one-out cross-validation

	**tested factor: genotype**	
	**model 1**<br>**weight ~ genotype + treatment + experiment**	model 2<br>weight ~ treatment + experiment
expected log predictive density	-335	
standard error	21.3	
	**tested factor: treatment**	
	**model 1**<br>**weight ~ genotype + treatment + experiment**	model 2<br>weight ~ genotype + experiment
expected log predictive density	-104.3	
standard error	13	
	**tested factor: genotype*treatment**	
	**model 1**<br>**weight ~ genotype * treatment + genotype + treatment + experiment**	model 2<br>weight ~ genotype + treatment + experiment
expected log predictive density	-23.2	
standard error	7.9	

B. Two-way ANOVA test

	Df	Sum Sq	Mean Sq	F value	Pr(>F)
**genotype**	5	2.28093	0.45619	198.417	**< 2.20E-16**
**treatment**	3	0.56645	0.18882	82.1251	**< 2.20E-16**
**experiment**	1	0.06414	0.06414	27.8961	**1.57E-07**
**genotype:treatment**	15	0.12689	0.00846	3.6795	**2.55E-06**

Supplementary Table 4. Estimation of *A. thaliana* host genotype importance in abundance changes in MixedCom. Two models were compared: [log10(cumulative isolate load) ~ treatment + genotype + experiment + error] and [log10(cumulative isolate load) ~ treatment * genotype + treatment + genotype + experiment + error]; thus the genotype * treatment coefficients were estimated for each barcoded isolate separately. Numbers are (expected log predictive density , standard error). Preferred model in bold.

Isolate	treatment * genotype	treatment + genotype
P1	(-4.59 , 1.18)	**(0 , 0)**
P2	(-1.71 , 2.51)	**(0 , 0)**
P3	(-4.47 , 1.03)	**(0 , 0)**
P4	(-2.89 , 2.15)	**(0 , 0)**
P5	(-4.25 , 1.01)	**(0 , 0)**
P6	(-2.96 , 1.9)	**(0 , 0)**
P7	(-3.29 , 1.86)	**(0 , 0)**
C1	(-3.3 , 1.58)	**(0 , 0)**
C2	(-3.5 , 1.66)	**(0 , 0)**
C3	(-0.68 , 2.56)	**(0 , 0)**
C4	(-1.64 , 2.36)	**(0 , 0)**
C5	(-0.78 , 2.59)	**(0 , 0)**
C6	(-1.78 , 2.37)	**(0 , 0)**
C7	(-1.97 , 2.17)	**(0 , 0)**

Supplementary Table 5. List of Differentially expressed genes (DEGs). Related to Figure 5A. Separate .xlsx file.

Supplementary Table 6. Overrepresented GO categories. Related to Figure 5B. Separate .xlsx file.

Supplementary Table 7. *Arabidopsis thaliana* accessions used.

1001 Genomes ID	Name	Latitude	Longitude	k-group
9769	HE-1	48.55	8.99	2
9782	Lu3-30	48.53	9.09	2
9802	Kus3-1	48.51	9.11	2
9807	Schl-7	48.6	9.22	2
9994	Ey15-2	48.43	8.77	2
10002	Tue-Wal2	48.53	9.04	2

Supplementary Table 8. Oligonucleotides used.

Name	Sequence	Purpose	Remarks
Bar1	GAATTCCTCGAGGTATCGCCTCCCTCGCGCCATCAGCCNNNNAANNNNTTNNNNTTNNNNATACATGACTGCTGTCGGCACAAGGGC	Barcode preparation	
Bar2	GGTACCGAGCTCCTATGCGCCTTGCCAGCCCGCTCAGGCCCTTGTGCCGACAGCAGTCATGT	Barcode preparation	
p1	GACATTCATCCGGGGTCAGC	Barcode validation	
p2	GTACCGGGCCCAAGCTTCTC	Barcode validation	
p3	GTGACTGGAGTTCAGACGTGTGCTCTTCCGATCTTGCAGGGCTTCCCAACCTTA	Barcode-PCR first step	
p4	ACACTCTTTCCCTACACGACGCTCTTCCGATCTCCTCCCTCGCGCCATCAG	Barcode-PCR first step	No frameshift
p5	ACACTCTTTCCCTACACGACGCTCTTCCGATCTACCTCCCTCGCGCCATCAG	Barcode-PCR first step	1 bp frameshift
p6	ACACTCTTTCCCTACACGACGCTCTTCCGATCTGACCTCCCTCGCGCCATCAG	Barcode-PCR first step	2 bp frameshift
p7	ACACTCTTTCCCTACACGACGCTCTTCCGATCTCGACCTCCCTCGCGCCATCAG	Barcode-PCR first step	3 bp frameshift
p8	ACACTCTTTCCCTACACGACGCTCTTCCGATCTTCGACCTCCCTCGCGCCATCAG	Barcode-PCR first step	4 bp frameshift
p9	ACACTCTTTCCCTACACGACGCTCTTCCGATCTTCGAGCCTCCCTCGCGCCATCAG	Barcode-PCR first step	5 bp frameshift
p10	AAGGGAATCAGGGGATCTTG	Barcode-qPCR	
p11	CCTCCCTCGCGCCATCAG	Barcode-qPCR	
p12	ACATGCTTTGATACAGCGGTGA	Plant-qPCR	*GIGANTEA*
p13	TGGATTCATTTCAGTCCTTGAGG	Plant-qPCR	*GIGANTEA*
515F-hamPCR	TCCCTACACGACGCTCTTCCGATCTCTGAGTGYCAGCMGCCGCGGTAA	hamPCR	V4, 16S rDNA
799R-hamPCR	GGAGTTCAGACGTGTGCTCTTCCGATCTTGCMGGGTATCTAATCCKGTT	hamPCR	V4, 16S rDNA
At.GI_F-hamPCR	TCCCTACACGACGCTCTTCCGATCTGTAAAGATAAATGGGTCATCTAA	hamPCR	*GIGANTEA*
At.GI_R-hamPCR	GGAGTTCAGACGTGTGCTCTTCCGATCTTCCTTCTGAACCGGTGTATTC	hamPCR	*GIGANTEA*

Supplementary Figures

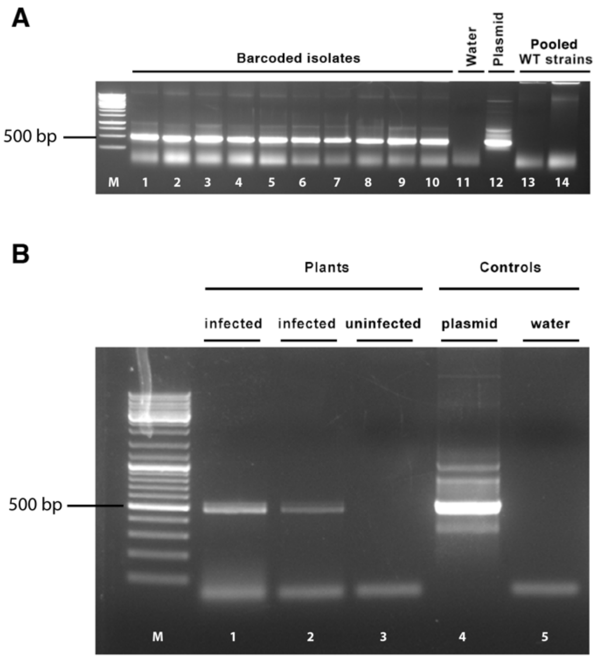

Supplementary Figure 1. Validation of barcode integration and barcode-PCR specificity by agarose gel electrophoresis of PCR amplified products. A. Validation of barcode integration to chosen isolates. Lanes 1–10 used DNA from examined barcoded isolates, lane 11 is water (negative control), lane 12 is the pUC18R6KT-mini-Tn7T plasmid into which a barcode was cloned (positive control), and lanes 13-14 are replicates of the 14 pooled parental (wild-type, WT) isolates. **B.** Validation of barcode-PCR specificity. Lanes 1-2 used DNA from plants infected with the 14 barcoded bacteria, lane 3 from an uninfected plant, lane 4 pUC18R6KT-mini-Tn7T plasmid (positive control), and lane 5 is water (negative control). Both infected and uninfected plants were grown in non-sterile conditions; barcode-specific primers yielded expected products of 522 bp. Lane M, DNA size marker. 500 bp marker indicated. For the unprocessed images see **Supplementary Figure10.**

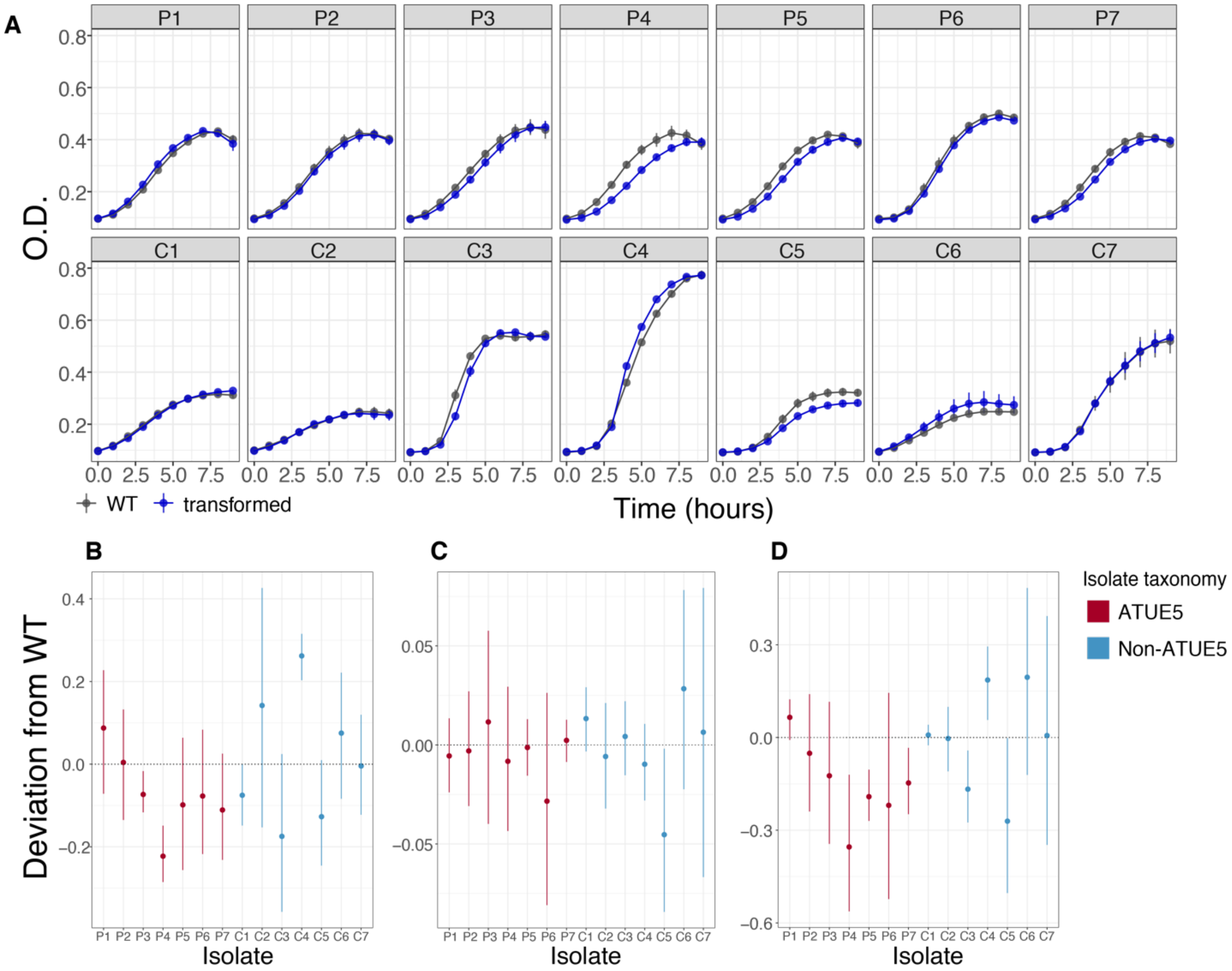

Supplementary Figure 2. Comparison of growth characteristics between non-barcoded parental isolates and their barcoded derivatives. A. Growth curves of the 14 parental isolates (WT) and their barcoded derivatives in Lysogeny broth (LB) over 10 hours, with OD_{600} recorded hourly. Mean ± SD, n=3. Change of barcoded isolates in comparison to their corresponding parents in **B.** growth rate, **C.** carrying capacity, and **D.** area under the curve. All three growth parameters were derived from the original growth curves. Dotted line indicates the n parental baseline for a given quantity. Median estimates ± 95% Bayesian credible intervals, n=3.

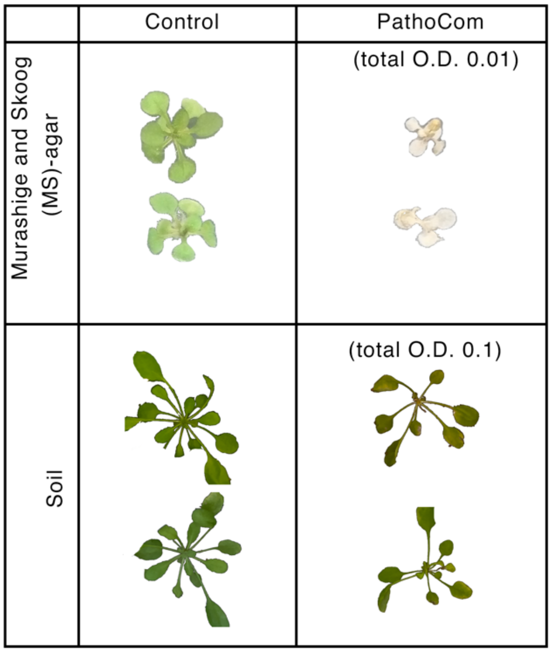

Supplementary Figure 3. Illustrative photos of control- and PathoCom-treated plants, grown in either MS-agar (sterile) or soil (unsterile). In both systems, the genotype Ey15-2 was used. For the MS-agar system, photos were taken 3-dpi, for the soil system 14-dpi. Sizes of plants are comparable within each system, but not between. Because images in the soil system were taken and parsed by pot automatically by a high-throughput imaging pipeline, some plant images were cropped.

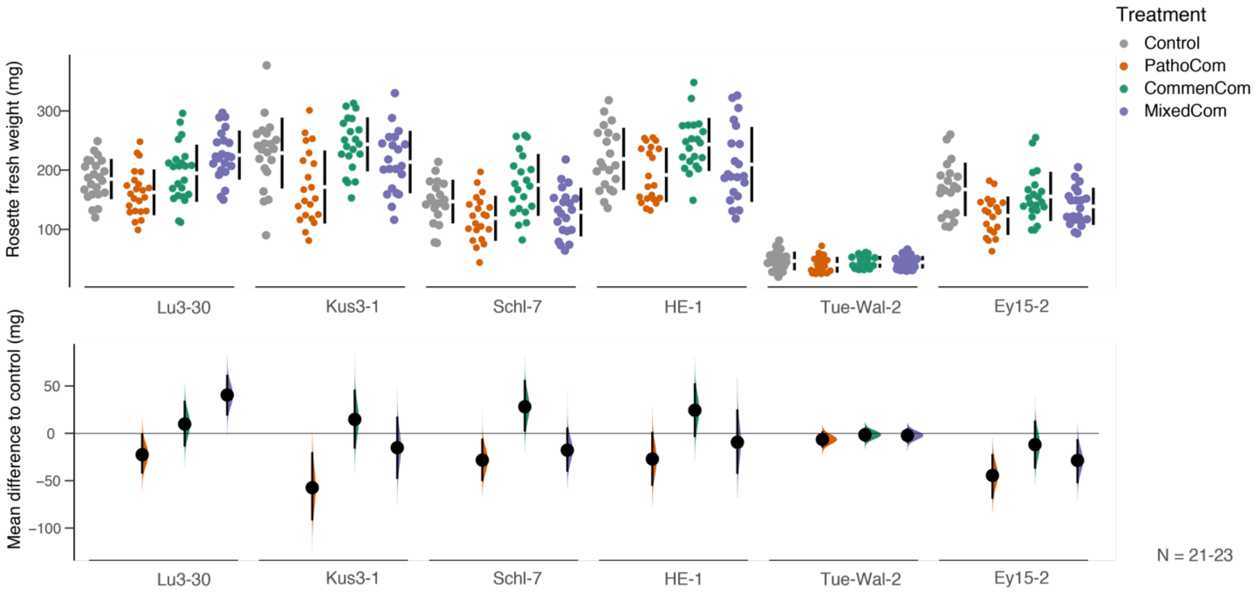

Supplementary Figure 4. Protection by commensal *Pseudomonas* strains is host dependent. Replicate experiment, related to Fig. 2 in main text. Each of the six *A. thaliana* genotypes used in this study was treated with Control, PathoCom, CommenCom and MixedCom. Fresh rosette weight was measured 12 dpi. The top panel presents the raw data, the breaks in the black vertical lines denote the mean value of each group, and the vertical lines themselves indicate standard deviation. The lower panel presents the mean difference to control, inferred from bootstrap sampling [32,33], indicating the distribution of effect sizes that are compatible with the data. 95% confidence intervals are indicated by the black vertical bars. n=21-23.

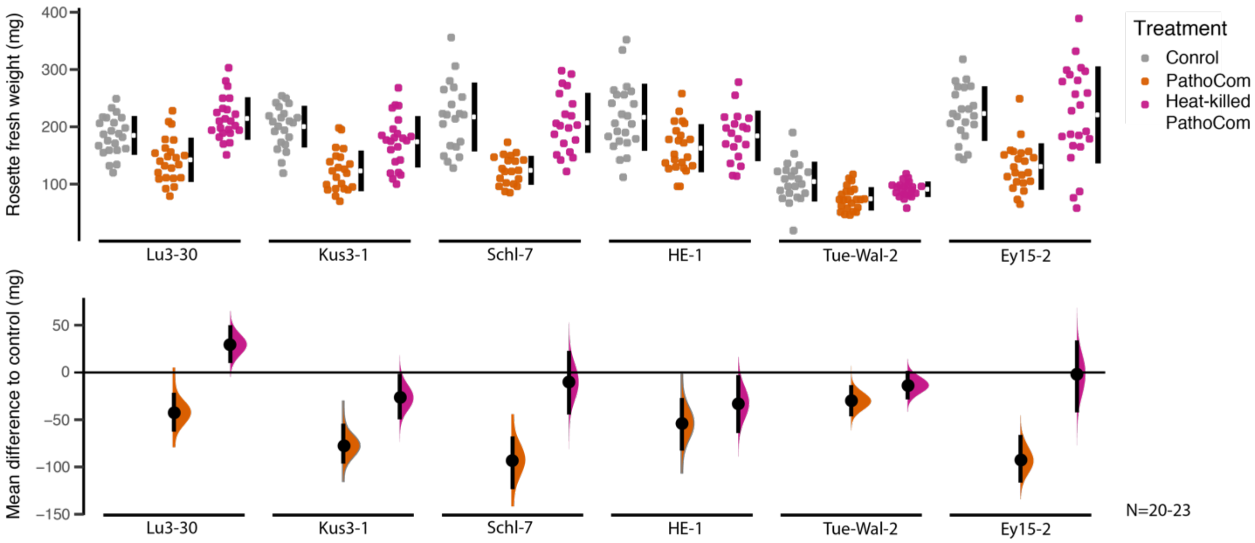

Supplementary Figure 5. Fresh rosette weight of plants treated with Control, PathoCom or heat-killed PathoCom. Each of the six *A. thaliana* genotypes used in this study was treated with control, PathoCom and heat-killed PathoCom inoculum, and fresh rosette weight was measured 12 dpi. The top panel presents the raw data, the breaks in the vertical black lines denote the mean value of each group, and the vertical lines themselves indicate standard deviation. The lower panel presents the mean differences to control, plotted as bootstrap sampling [32,33], indicating the distribution of effect sizes that are compatible with the data. 95% confidence intervals are indicated by the black vertical bars.

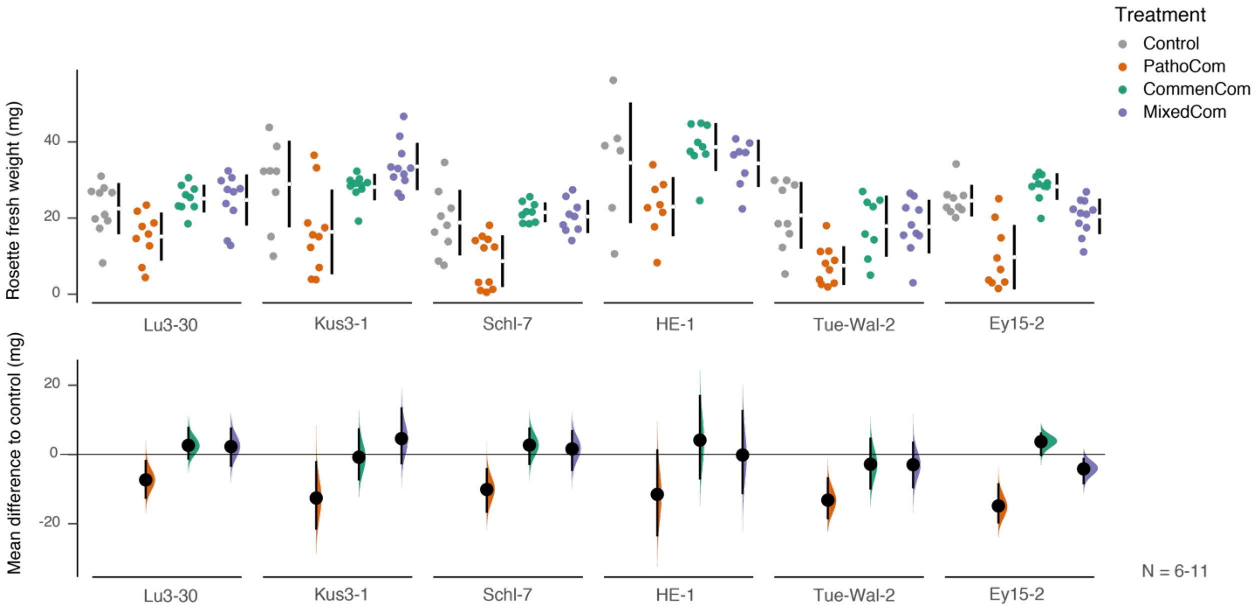

Supplementary Figure 6. Protection by commensal *Pseudomonas* strains is host dependent in an axenic environment. Each of the six *A. thaliana* genotypes used in this study was treated with Control, PathoCom, CommenCom and MixedCom. Fresh rosette weight was measured 7 dpi. The top panel presents the raw data, the breaks in the black vertical lines denote the mean value of each group, and the vertical lines themselves indicate standard deviation. The lower panel presents the mean difference to control, inferred from bootstrap sampling [32,33], indicating the distribution of effect sizes that are compatible with the data. 95% confidence intervals are indicated by the black vertical bars. Shown here are the results of one experiment. This experiment was performed in an axenic MS-agar system, while the other experiments in this study were done with non-sterile soil-grown plants. n=6-11.

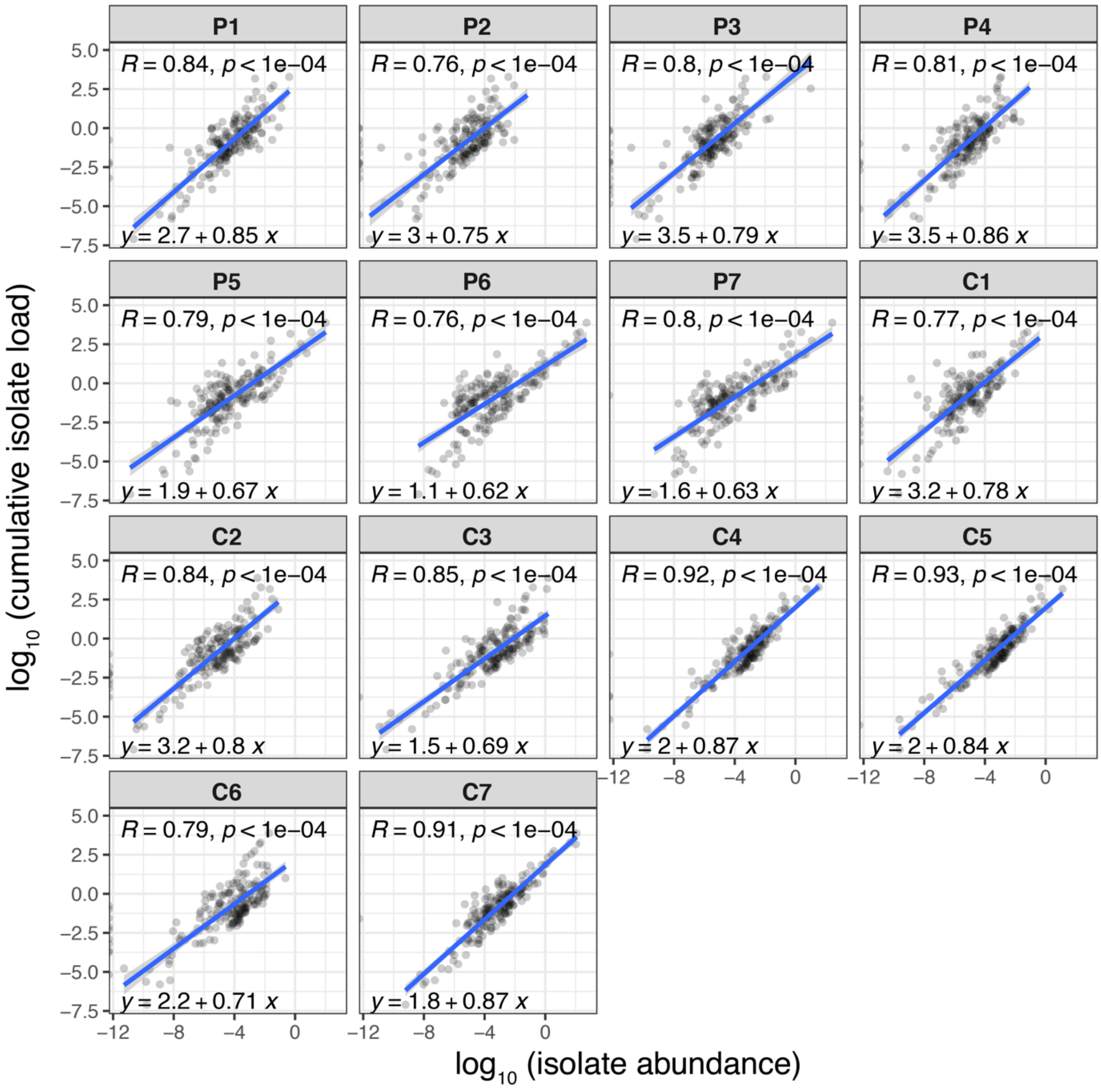

Supplementary Figure 7. Correlations between the absolute abundance of each isolate and the cumulative bacterial abundance in MixedCom. Each panel represents an individual isolate. Pearson correlation (R) and p-value (p) are given on top, and the matching linear equation at the bottom of each panel. Shaded areas indicate 95% confidence intervals of the correlation curve.

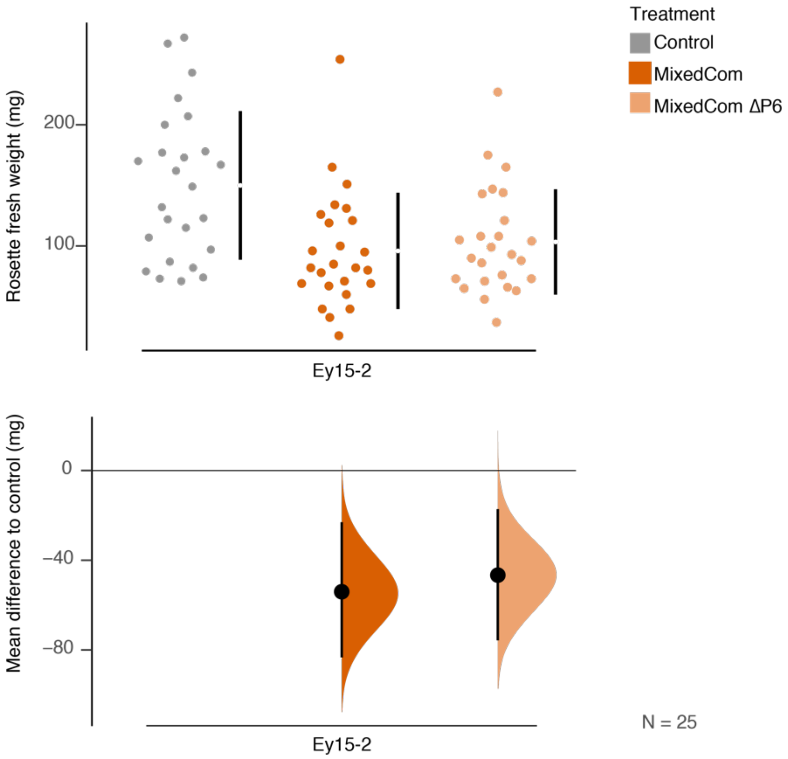

Supplementary Figure 8. Fresh rosette weight of Ey15-2 plants treated with Control, PathoCom or PathoCom without P6 (PathoCom ΔP6). Fresh rosette weight was measured 12 dpi. The top panel presents the raw data, the breaks in the vertical black lines denote the mean value of each group, and the vertical lines themselves indicate standard deviation. The lower panel presents the mean differences to control, plotted as bootstrap sampling [32,33], indicating the distribution of effect sizes that are compatible with the data. 95% confidence intervals are indicated by the black vertical bars. n=25.

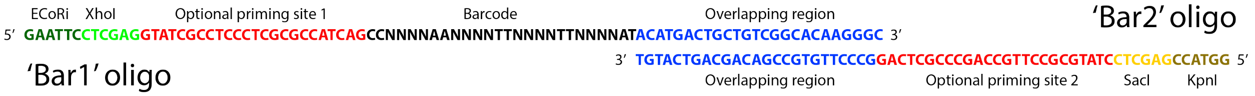

Supplementary Figure 9. Illustration of barcode design. Two single-stranded oligos were synthesized: ‘Bar1’ and ‘Bar2’. N indicates random nucleotides.

A B

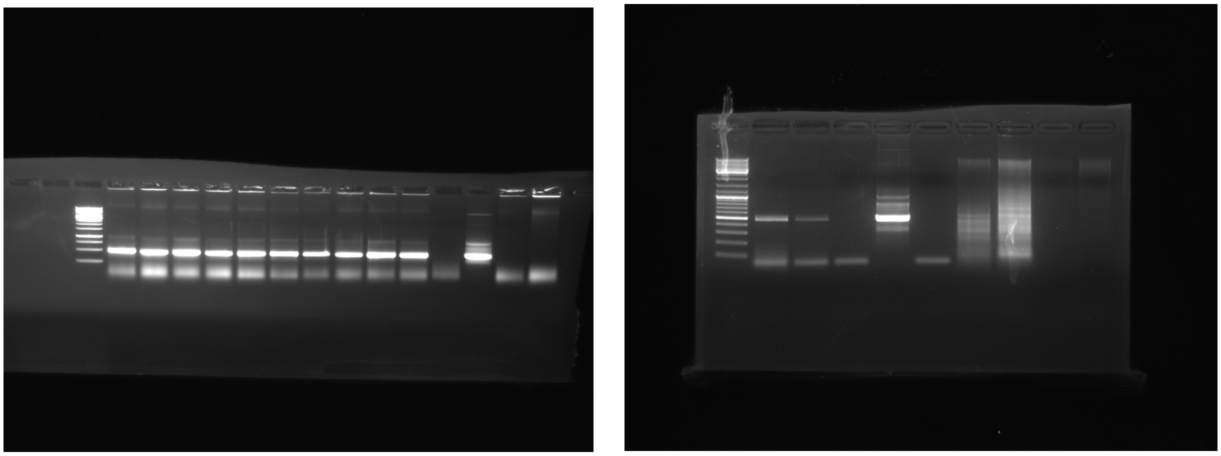

Supplementary Figure 10. Unprocessed images of the gels used in Supplementary Figure 1. Panels A and B corresponds to the same panels in **Supplementary Figure 1**.
