# Supplementary material for: Commensal Pseudomonas strains facilitate protective response against pathogens in the host plant
Source: Nat Ecol Evol. 2022 Feb 24;6(4):383–96. doi: 10.1038/s41559-022-01673-7 (PMC8986537; doi:10.1038/s41559-022-01673-7)
Supplement: Supplementary file 2 — Reporting Summary. [file 41559_2022_1673_MOESM2_ESM.pdf]

nature portfolio

Corresponding author(s): Detlef Weigel

Last updated by author(s): Nov 30, 2021

Reporting Summary

Nature Portfolio wishes to improve the reproducibility of the work that we publish. This form provides structure for consistency and transparency in reporting. For further information on Nature Portfolio policies, see our Editorial Policies and the Editorial Policy Checklist.

Statistics

For all statistical analyses, confirm that the following items are present in the figure legend, table legend, main text, or Methods section.

n/a	Confirmed	
☐	☒	The exact sample size (*n*) for each experimental group/condition, given as a discrete number and unit of measurement
☐	☒	A statement on whether measurements were taken from distinct samples or whether the same sample was measured repeatedly
☐	☒	The statistical test(s) used AND whether they are one- or two-sided<br>*Only common tests should be described solely by name; describe more complex techniques in the Methods section.*
☐	☒	A description of all covariates tested
☐	☒	A description of any assumptions or corrections, such as tests of normality and adjustment for multiple comparisons
☐	☒	A full description of the statistical parameters including central tendency (e.g. means) or other basic estimates (e.g. regression coefficient) AND variation (e.g. standard deviation) or associated estimates of uncertainty (e.g. confidence intervals)
☐	☒	For null hypothesis testing, the test statistic (e.g. *F*, *t*, *r*) with confidence intervals, effect sizes, degrees of freedom and *P* value noted<br>*Give P values as exact values whenever suitable.*
☐	☒	For Bayesian analysis, information on the choice of priors and Markov chain Monte Carlo settings
☐	☒	For hierarchical and complex designs, identification of the appropriate level for tests and full reporting of outcomes
☐	☒	Estimates of effect sizes (e.g. Cohen's *d*, Pearson's *r*), indicating how they were calculated

Our web collection on statistics for biologists contains articles on many of the points above.

Software and code

Policy information about availability of computer code

Data collection	Illumina HiSeq 3000
Data analysis	RAxML (v.0.6.0), iTOL (online tool), BWA-MEM (v 0.7.17-r1188), Cytoscape (v3.7.0) and the plugin BiNGO (v3.0.3), REVIGO (online tool) , Bio-Rad CFX Manager (v3.1), STAR (v.2.6.0), R environment version 3.5.1 and the R packages: "Growthcurver", "dabestr", "rstanarm", "BayesFactor", "Loo", "vegan", "pairwiseAdonis", "Hmisc", "DESeq2 (v.1.22.2)".

For manuscripts utilizing custom algorithms or software that are central to the research but not yet described in published literature, software must be made available to editors and reviewers. We strongly encourage code deposition in a community repository (e.g. GitHub). See the Nature Portfolio guidelines for submitting code & software for further information.

Data

Policy information about availability of data

All manuscripts must include a data availability statement. This statement should provide the following information, where applicable:

- Accession codes, unique identifiers, or web links for publicly available datasets
- A description of any restrictions on data availability
- For clinical datasets or third party data, please ensure that the statement adheres to our policy

RNA sequencing data have been deposited with the European Nucleotide Archive (ENA) under study accession number PRJEB41069. Normalized expression values can be found in Supplementary data1. All bacterial accession codes (from Karasov et al.) 2018 are provided in Table S1. All plant accession code are provided in Table S5. The genome-barcoded Pseudomonas isolates used in this study can be obtained upon request.

Field-specific reporting

Please select the one below that is the best fit for your research. If you are not sure, read the appropriate sections before making your selection.

☒ Life sciences ☐ Behavioural & social sciences ☐ Ecological, evolutionary & environmental sciences

For a reference copy of the document with all sections, see nature.com/documents/nr-reporting-summary-flat.pdf

Life sciences study design

All studies must disclose on these points even when the disclosure is negative.

Sample size	In a trial experiment we observed that the variation in weight of bacterial-infected plants was higher than control. This was in agreement with a high variability of the bacterial load within treatments (among different samples of the same treatment). This high availability was probably due to the infection system we used (spraying bacteria). In this trial experiment we used 10 replicates, and observed that in some cases the variation was too high to decide whether there is a trend or a signal. To account for this high variation, we decided to double the number of plants per condition (treatment * host genotype), reaching what we realized as a borderline for the practicality of such a big-scale experiment. We then tested 20 replicates and realized that it provides enough power to differentiate main trends from noise.
Data exclusions	- For bacterial abundance estimation - samples with less than a total of 200 hits were discarded or resequenced (mean=15709.8). We used a maximum of 14 isolates (in the MixedCom). The rationale was to include samples with high enough coverage for this low complexity (minimum of 14.2 reads per isolate in MixedCom, and 24.4 reads for PathoCom and CommenCom).<br>- For RNA-seq analysis: genes with average counts of less than five were excluded from the analysis, following the practice of Nobori et al. (Nature plants, 2020).
Replication	Main experiments were replicated at least twice (as detailed in the figure legend). We tested the batch (i.e. experiment) effect using a linear model and by independently analyze different experiments with the same pipeline. In all cases we found that major trends which are reported were reproducible.
Randomization	In each experiments, the tray location of the plant genotypes and bacterial treatments were random. Trays were also randomly placed in the growth chamber and replaced every 2-3 days.
Blinding	Samples collection was done blindly, as the plants were marked by an index rather than by plant genotype or treatment.

Reporting for specific materials, systems and methods

We require information from authors about some types of materials, experimental systems and methods used in many studies. Here, indicate whether each material, system or method listed is relevant to your study. If you are not sure if a list item applies to your research, read the appropriate section before selecting a response.

Materials & experimental systems

n/a	Involved in the study	
☒	☐	Antibodies
☒	☐	Eukaryotic cell lines
☒	☐	Palaeontology and archaeology
☒	☐	Animals and other organisms
☒	☐	Human research participants
☒	☐	Clinical data
☒	☐	Dual use research of concern

Methods

n/a	Involved in the study	
☒	☐	ChIP-seq
☒	☐	Flow cytometry
☒	☐	MRI-based neuroimaging
