# Supplementary material for: Commensal Pseudomonas strains facilitate protective response against pathogens in the host plant
Source: Nat Ecol Evol. 2022 Feb 24;6(4):383–96. doi: 10.1038/s41559-022-01673-7 (PMC8986537; doi:10.1038/s41559-022-01673-7)
Supplement: Supplementary file 3 — Peer Review Information. [file 41559_2022_1673_MOESM3_ESM.pdf]

naturereseach

Peer Review Information

Journal: Nature Ecology & Evolution
Manuscript Title: Protective host-dependent antagonism among *Pseudomonas* in the *Arabidopsis* phyllosphere
Corresponding author name(s): Detlef Weigel

Editorial Notes:

Reviewer Comments & Decisions:

Decision Letter, initial version:

18th May 2021

*Please ensure you delete the link to your author homepage in this e-mail if you wish to forward it to your co-authors.

Dear Professor Weigel,

Your manuscript entitled "Protective host-dependent antagonism among <i>Pseudomonas</i> in the <i>Arabidopsis</i> phyllosphere" has now been seen by three reviewers, whose comments are attached. The reviewers have raised a number of concerns which will need to be addressed before we can offer publication in Nature Ecology & Evolution. We will therefore need to see your responses to the criticisms raised and to some editorial concerns, along with a revised manuscript, before we can reach a final decision regarding publication.

Although we are willing to overrule Reviewer 1 on the concerns about novelty, we expect the revision to clarify the advance over related papers in the field. Moreover, Reviewers 1 and 3 both raises concerns about inadequate control conditions (i.e. use of sterile soils in some conditions) and we hope this can be addressed. Finally, the claims should be supported / quantified with statistical evidence as suggested by Reviewer 2.

We therefore invite you to revise your manuscript taking into account all reviewer and editor comments. Please highlight all changes in the manuscript text file.

We are committed to providing a fair and constructive peer-review process. Do not hesitate to contact

us if there are specific requests from the reviewers that you believe are technically impossible or unlikely to yield a meaningful outcome.

When revising your manuscript:

* Include a “Response to reviewers” document detailing, point-by-point, how you addressed each reviewer comment. If no action was taken to address a point, you must provide a compelling argument. This response will be sent back to the reviewers along with the revised manuscript.

* If you have not done so already please begin to revise your manuscript so that it conforms to our Article format instructions at http://www.nature.com/natecolevol/info/final-submission. Refer also to any guidelines provided in this letter.

* Include a revised version of any required reporting checklist. It will be available to referees (and, potentially, statisticians) to aid in their evaluation if the manuscript goes back for peer review. A revised checklist is essential for re-review of the paper.

Please use the link below to submit your revised manuscript and related files:

[REDACTED]

Note: This URL links to your confidential home page and associated information about manuscripts you may have submitted, or that you are reviewing for us. If you wish to forward this email to co-authors, please delete the link to your homepage.

We hope to receive your revised manuscript within four to eight weeks. If you cannot send it within this time, please let us know. We will be happy to consider your revision so long as nothing similar has been accepted for publication at Nature Ecology & Evolution or published elsewhere.

Nature Ecology & Evolution is committed to improving transparency in authorship. As part of our efforts in this direction, we are now requesting that all authors identified as ‘corresponding author’ on published papers create and link their Open Researcher and Contributor Identifier (ORCID) with their account on the Manuscript Tracking System (MTS), prior to acceptance. ORCID helps the scientific community achieve unambiguous attribution of all scholarly contributions. You can create and link your ORCID from the home page of the MTS by clicking on ‘Modify my Springer Nature account’. For more information please visit please visit www.springernature.com/orcid.

Please do not hesitate to contact me if you have any questions or would like to discuss these revisions further.

We look forward to seeing the revised manuscript and thank you for the opportunity to review your work.

[REDACTED]

Reviewer expertise:

Reviewer #1:

Reviewer #2:

Reviewer #3:

Reviewers' comments:

Reviewer #1 (Remarks to the Author):

Shalev et. al provide a holistic examination of how intra-genus microbe-microbe interactions modulate specific host transcriptional and physiological responses. Specifically, the authors examine how Pseudomonas isolates with contrasting plant-elicited phenotypes (pathogenicity and commensalism) coexist in natural microbial communities inhabiting the phyllosphere of plants. To explore this, the authors employed a combination of re-constitution experiments utilizing synthetic communities across a panel of diverse A. thaliana accessions coupled with host phenotyping and community profiling.

1.Novelty: Despite the experiments being clearly executed and interpreted, this study lacks novelty in its main biological findings and serves more as a synthesis of established phenomenon occurring within plant-microbiomes.
Studies employing distinct levels of inoculum complexity (mono-association, duo-association, complex SynComs) have shown the relevance of microbe-microbe interactions (Garrido-Oter et. al. 2018 and Finkel et. al 2020) to modulate specific host morphometric and transcriptional responses. Furthermore, transcriptional profiling in the context of assembled SynComs with members exhibiting contrasting mono-association host-elicited responses (pathogenicity, commensalism) have reported (Finkel et. al 2020, Salas-Gonzalez et. al 2021 and Teixeira et. al 2021) the activation of immune-elicited responses by the host. Finally, Hu et. al 2016 (Probiotic Diversity Enhances Rhizosphere Microbiome Function and Plant Disease Suppression) have explored the effect of the intra-genus diversity within Pseudomonas isolates to modulate host responses to biotic stresses. In my opinion this study does not fulfill the novelty merits to be published in a top-tier journal such as Nature Ecology and Evolution.

2. Biological significance:

2a. The authors selected 14 strains, for their SynCom experiments, based on the number of similar isolates in their Pseudomonas collection. The authors should mention what is the total abundance of these isolates from Karasov. et. al. amplicon surveys. I do not see the ecological significance of the isolates chosen with the exception of those that come from OTU5.

2b. I appreciate the methodological effort to genome-barcode the 14 isolates, nevertheless I think if the authors had picked other representative isolates in their collection, this would have not been necessary. The authors should state clearly the ecological relevance of their 14 isolates to better justify the employing of the genome-barcoding.

2c. The authors decided to perform their SynCom experiments in an open system setup. This decision hinders the interpretability of the intra-Pseudomonas competition by confounding the effect that these isolates exert over the host by the effect that other microbiota members exert over the host. There is

always the possibility that the interaction between SynCom:OtherMicrobiota is eliciting the specific host phenotypes the authors measured either with their morphometric or transcriptomic phenotyping. The authors should perform an experiment in gnotobiotic conditions showing that the contribution of the Other Microbiota members is negligible thus further supporting their decision to use an open system setup.

2d. In addition to point 2c, the authors should show the abundance of the other microbiota members in their system using an amplicon-based survey. This point is particularly relevant for the case of other natural inhabiting Pseudomonas isolates. Again, the results observed in Table 1 could be confounded by the biological interaction between SynCom:OtherMicrobiota.

2e. In Figure 2 the authors show the effect of the different SynCom treatments across the A.thaliana genotypes. It is clear that the different genotypes exhibit different magnitude of response to the distinct SynComs but all of them exhibit the same shape in the trend. To confirm this, the authors should add an interaction model and discuss this result further. Without the existence of a significant interaction in the model, these results look more like pseudo replication of the phenomenon and therefore requires toning down on the "host-dependent" claim.

2f. For the RNA-Seq experiment, the authors sampled treated plants at three and four day after infection, nevertheless there is not a clear justification for these overlapping timepoints. Have the microbial communities reached stability at this point?

Reviewer #2 (Remarks to the Author):

Summary:
How plants select commensals over pathogens is poorly understood. The manuscript by Shalev et al. shows that commensal bacterial communities and the plant host interact to inhibit bacterial pathogens in the phyllosphere. This work also shows that plants are able to tolerate higher loads of commensals compared to pathogens. This study describes how either specific strains of commensals or commensal communities are able to inhibit pathogen abundance and other commensal strains either by direct bacterial competition or in combination with the host.

Shalev et al. provide clear hypotheses and experiments addressing the interaction between bacterial community members and the plant host inhibiting pathogen growth in the phyllosphere. While the experimentation is clear, I am concerned with the statistical significance of the data, or more specifically, a lack of statistical analysis to support several of the major conclusions. Some of the data presented are from single experimental replicates or show little to no statistical analysis to determine whether the differences between treatments are significant. It would be helpful for the manuscript to show that the conclusions are drawn from meaningful differences between the tested conditions and to highlight replicability. Once addressed, this work provides a clear and exciting look into the complex interactions that occur between host and microbes in establishing a healthy plant microbiome.

Major concerns:
Figure 2, only shows data from one experimental replicate. Results/conclusions are based off of two

replicates and the second one isn’t shown. No stats are presented to test whether the host-dependent commensal protection is significant. Please add statistical analysis and show both replicates (the second can be shown in the supplement).

Figure 3B, there is a large amount of variance in the regression slopes for each treatment. It would be useful to know the R^2 for each to know how the variance in rosette fresh weight is related to bacterial load. Additionally, the authors should test if the differences in slope are significantly more negative to support the claim that the slope is more negative.

Figure 4B, no stats are available to show which bacteria isolates have loads that are significantly different when compared to the Mixed Community. Similar critique for Figure 4E.

Figure 6C, statistical analysis would be useful for determining whether the bacterial isolate P6 has a significant effect on Ey15-2 rosette weight and for judging the changes in abundance in Figure 6B.

Please describe how abundance of mean difference was calculated in the methods. A reference is included, but this metric is used frequently enough that it would be useful to have a brief description within the methods. Additionally, please define the y-axis in the legends in a bit more detail so the reader has a sense of what the scale means.

Minor concerns:
Line 99, population should be plural

Line 170, change to “explained 4-26% of compositional variation” to mirror a similar statement seen in line 167.

Line 138: I don’t think “equimolar” is appropriate for pooling bacteria. Do you mean equivalent estimated CFUs/OD600 readings?

Line 139: I think you mean twice the number of cells per unit volume and not just the twice the total number of cells?

In some cases more method details are needed within the results to follow how the experiments were done. For instance, lines 235-7 bacterial load was quantified based on barcoded read data, but this isn’t specified.

Line 206 “on” should be “of”

Line 307 “in at least in two” should be “in at least two”

Line 328 “in plana” should be “in planta”

Reviewer #3 (Remarks to the Author):

Dear Editor,
Thank you for inviting me to review the manuscript. The authors Shalev et al., presented a very

interesting story of 6 Arabidopsis genotypes interact with different groups of commensal, pathogenic and mixed Pseudomonas synComs on their leaves, and discovered microbe-microbe driven plant phenotypes, and the potential plant recognition mechanism behind. Particularly, the authors presented a nice genome-barcoding method to each Pseudomonas strain, which could facilitate both tracking and quantification of each Pseudomonas isolate. However, some important aspects should be clarified and improved before the paper can be accepted for publication. Therefore, my recommendation for the manuscript would be a moderate revision. Detailed major and minor questions and comments are listed as following:

Major comments:
1. What was the rationale of choosing only Pseudomonas lineages for the CommenCom, but not a more diverse group of different bacteria to mimic more the natural conditions?

2. For the three synthetic communities, PathoCom, CommenCom and MixedCom, did the authors test if each strain can grow well within each community? In another word, for instance, within the PathoCom or the CommenCom, did the 7 strains grow equally well when mixing them together, but not certain strain(s) dominate and take over the communities? This is to exclude the possibility of the plant phenotype observed later come from specific strain(s), but not from the communities together (or interactions). How did the authors test this? Please specify and provide the supporting info

3. Line 148, why in Figure S4, Ey15-2 was represented? Do the other cultivars show the similar phenotype as Ey15-2? Line 152, Why choose to inoculate synthetic communities after 21 days of sowing?

4. Line 193-203, How to rule out the plant phenotype (weight loss) is not due to root microbiome effect (as well) since the authors used non-sterile soil? A suggestion is to add sterile soil treatment which would help to answer this for Figure 2.

5. Line 253-255, figure 3B. Is there a significant difference of the regression slopes of PathoCom and CommenCom? It looks like there is overlap which indicates no significant difference. Please clarify the statistical analysis.

6. Figure 4B. Line 280-282. I didn't understand this statement. Since in Line 278-280, it stated that "pathogenic isolates were compared between PathoCom and MixedCom". Why in Line 281, the statement is the abundance of pathogens was significantly lower in MixedCom? How exactly to interpret figure 4B?

7. Line 327-328, it stated that "no commensal isolate was inhibited in planta, among communities". How about C6? what does the negative value indicate in the figure 4B?

8. Line 350, Though pathogen inhibition seemed to be independent of the host genotype, what was the rationale of choosing HE-1 here? Why not choose the most resistant (Lu3-30, TueWal-2) and most susceptible (Ey15-2) plant genotype or Lu3-30 the authors chose in next experiment?

9. Line 362, what was the rationale of choosing the genotype Lu3-30 for the RNA-seq experiment? And what was the rationale of sampling on 3 and 4-dpi, but not 12-dpi, which is consistent with the spray assay for Figure 2?

10. Line 417-419, the marker genes (PR1 and PR5) expression in MixedCom- and CommenCom-infected hosts are very similar, but CommenCom-infected hosts have the most promoted effect on the weight than MixedCom- infected hosts. What could be the reason for this? Does it mean that SAR is not the main or only explanation?

Minor comments:
1. Line 37, "and are dictated by the balance of inhibition and facilitation of growth". It is not clear whether it is for plant or microbial growth or both.

2. Line 52-53, There is no logical relationship between "other environmental factors" and "including other resident microbes". Please rephrase.

3. Line 210, the effect of PathoCom on the genotype Ey15-2 weight should be emphasized in Line 195-198.

4. Line 228, the second experiment results should be shown in supplementary info.

5. Line 342, What do "higher-order interactions" refer here? Please specify.

********************END********************

Author Rebuttal to Initial comments

NATECOLEVOL-210413396

Please note that only major changes in the manuscript were highlighted in yellow.

Reviewer #1 (Remarks to the Author):

Shalev et. al provide a holistic examination of how intra-genus microbe-microbe interactions modulate specific host transcriptional and physiological responses. Specifically, the authors examine how Pseudomonas isolates with contrasting plant-elicited phenotypes (pathogenicity and commensalism) coexist in natural microbial communities inhabiting the phyllosphere of plants. To explore this, the authors employed a combination of re-constitution experiments utilizing synthetic communities across a panel of diverse A. thaliana accessions coupled with host phenotyping and community profiling.
1.Novelty: Despite the experiments being clearly executed and interpreted, this study lacks novelty in its main biological findings and serves more as a synthesis of established phenomenon occurring within plant-microbiomes.
Studies employing distinct levels of inoculum complexity (mono-association, duo-association, complex SynComs) have shown the relevance of microbe-microbe interactions (Garrido-Oter et. al. 2018 and Finkel et. al 2020) to modulate specific host morphometric and transcriptional responses. Furthermore, transcriptional profiling in the context of assembled SynComs with members exhibiting contrasting mono-association host-elicited responses (pathogenicity, commensalism) have reported (Finkel et. al 2020, Salas-Gonzalez et. al 2021 and Teixeira et. al 2021) the activation of immune-elicited responses by the host. Finally, Hu et. al 2016 (Probiotic Diversity Enhances Rhizosphere Microbiome Function and Plant Disease Suppression) have explored the effect of the intra-genus diversity within Pseudomonas isolates to modulate host responses to biotic stresses. In my opinion this study does not fulfill the novelty merits to be published in a top-tier journal such as Nature Ecology and
Evolution.

Thank you for acknowledging the clarity of experiments and their interpretation. We apologize that we perhaps did not perfectly frame our study to explain how it is set apart from the cited studies, which have of course advanced our understanding of how bacterial communities interact with the host plant.

Garrido-Oter et al. (2018) presented the result of a single experiment that examined the effects of all pairwise combinations of four rather different bacterial species on root growth promotion. The conclusion was that phenotypes were additive. Finkel et al. (2020) investigated a complex SynCom of 185 diverse bacteria, and from these identified a group of closely related, functionally interchangeable strains that suppressed root growth inhibition by the other members of the SynCom. Teixera et al. (2021) similarly describe that several members in a complex SynCom can all suppress immune responses. Salas-Gonzalez et al. (2021) focused on how bacteria modify root diffusion barriers, which in turn affect microbiota composition, using knockout mutants in a single genetic background infected with a single, taxonomically diverse 41-member SynCom. Finally, Hu et al. (2016) associated diversity and niche breadth of *Pseudomonas* communities with protection against the pathogen *Ralstonia solanacearum*. Similarly to our work, these studies made use of SynComs to address different aspects of the plant-microbiota relationships.

Our work is distinguished from the prior work in three major ways:

1. The previous studies emphasized the interchangeability of even sometimes distantly related strains. In contrast, we show that different *Pseudomonas* members of either a pathogenic or commensal community are interchangeable only to a limited extent. In particular, we demonstrate that highly similar *Pseudomonas* strains (members of the same OTU) have differential competitive ability within and among commensal and pathogenic communities. The conclusions follow both from *in vitro* pairwise interactions, and *in vivo* interactions within a moderately complex SynCom. Finally, going further than the previous studies, we demonstrate that the effects of the microbes on plant health depend on the host genotype, which we deduce from examining community shifts of the three SynComs in six plant genotypes, and linking these shifts to plant health.
2. Several of the previous studies examined shifts in *relative* abundance among SynCom members. In contrast, we also measured *absolute* abundance. The drawbacks of the compositional nature of conventional 16S rDNA type of measurements are well appreciated (Gloor et al. 2017). Several

mathematical tools have developed to reduce false inferences, but certainty can only come from measuring absolute abundance per isolate. Such data allowed us to (i) reveal taxon-specific inhibition, (ii) associate the load per isolate with plant weight, revealing that plant health cannot be simply predicted by total *Pseudomonas* load, and (iii) study how different plant genotypes differentially affect isolate load.

3. The MixedCom result could be interpreted purely in a diversity framework, similarly to the work of Hu et al. (2016),, since the MixedCom is more diverse than the PathoCom. However, the CommenCom and PathoCom both include 7 members, and therefore have equal strain diversity. Moreover, we show that adding a single commensal to the 7-member PathoCom replicates many properties of the 14-member MixedCom. Lastly, Hu et al. (2016) found that *in vitro* activity of commensal *Pseudomonas* is highly predictable for their suppressive ability *in planta*, suggesting that host-microbe interactions play only a minor role. In contrast, we find substantial differences between *in vitro* and *in vivo* results. Specific commensals differ in their ability to suppress specific pathogens *in vitro* and *in planta*, with one pathogen being inhibited only *in planta*, regardless of the tested commensal. This implies that host-mediated interactions (microbe-host-microbe) are an important facet of pathogen suppression.

To better frame our work, we have added some of the above to the introduction of our manuscript. To conclude, what differentiates our work from the previous studies is the use of different commensal, pathogen and host genotypes that all co-exist in nature, making our work ecologically particularly relevant. Similarly, there is so far little work on differences in function between closely related microbes in a SynCom context.

2. Biological significance:
2a. The authors selected 14 strains, for their SynCom experiments, based on the number of similar isolates in their Pseudomonas collection. The authors should mention what is the total abundance of these isolates from Karasov. et. al. amplicon surveys. I do not see the ecological significance of the isolates chosen with the exception of those that come from OTU5.

Thank you for this suggestion. We updated Table S1 to provide information on the abundance of the different lineages in the Karasov et al. (2018) collection, in which about 15% of *Pseudomonas* isolates were not OTU5, with OTU5 abundance in the field fluctuating and depending on the season. Importantly, while OTU5 bloomed in many *A. thaliana* plants and was the dominant taxon, this was not true for all plants, and there were definitely plants with similar OTU5 and non-OTU5 loads. Thus, we maintain that our work is ecologically realistic. We selected the most abundant strains from the Karasov et al. (2018) collection, based on core-genome similarity, a much finer resolution than the 16S rDNA/OTU classification. Note that Karasov et al. (2018) had already documented major differences in pathogenicity among OTU5 isolates. In agreement, we find differences in field abundance between OTU5 strains, exemplified by P1 and P3, with population abundances comparable to those of non-OTU5 strains (Figure 1B).

2b. I appreciate the methodological effort to genome-barcode the 14 isolates, nevertheless I think if the authors had picked other representative isolates in their collection, this would have not been necessary. The authors should state clearly the ecological relevance of their 14 isolates to better justify the employing of the genome-barcoding.

We chose our 14 strains based on the fact that they were among the most common strains, which we believe makes the study more ecologically relevant than if we had chosen strains based on an arbitrary marker gene that allows amplicon-based differentiation. Note that it is almost certainly not trivial to find such a marker gene. As importantly, because most experiments were carried out in non-sterile conditions, these amplicons would have picked up members of the background microbiota. We also note that when we started the project, there was no other high-throughput method for measuring absolute bacterial load. (It is only recently that we have introduced a method that does not require the extra qPCR step anymore and that now relies only on amplicon sequencing (Lundberg et al. 2020)). Thus, we were able to obtain precise estimates of absolute abundance per individual isolates (isolate/plant chromosomes), which is still the exception.

2c. The authors decided to perform their SynCom experiments in an open system setup. This decision hinders the interpretability of the intra-Pseudomonas competition by confounding the effect that these isolates exert over the host by

the effect that other microbiota members exert over the host. There is always the possibility that the interaction between SynCom:OtherMicrobiota is eliciting the specific host phenotypes the authors measured either with their morphometric or transcriptomic phenotyping. The authors should perform an experiment in gnotobiotic conditions showing that the contribution of the Other Microbiota members is negligible thus further supporting their decision to use an open system setup.

This is a very good point, although one could argue that the open system is more ecologically realistic. Nevertheless, we agree that as presented, the host effects could also be mediated by the host modulating the background microbiota and the background microbiota in turn affecting our *Pseudomonas* strains. We have therefore added the results from an axenic experiment (Fig S9), which broadly confirms our main conclusions: (i) protection by commensals, and (ii) lack of full protection in Ey15-2. This does not exclude that environmental microbiota enhances or dampens some of the observed effects, but if they do, they do so in a general manner, and they are not essential for the observed effects; thank you for suggesting this important addition!

2d. In addition to point 2c, the authors should show the abundance of the other microbiota members in their system using an amplicon-based survey. This point is particularly relevant for the case of other natural inhabiting Pseudomonas isolates. Again, the results observed in Table 1 could be confounded by the biological interaction between SynCom:OtherMicrobiota.

That is an important issue. Indeed, since we are working with locally common *Pseudomonas* strains, one concern might be that our infections are swamped by similar strains picked up by the plants from the environment. We therefore quantitatively profiled total bacterial load in infected plants using host-associated microbe PCR (hamPCR), allowing us to compare the amount of *Pseudomonas* versus non-*Pseudomonas* in SynCom-infected plants and control-infected plants. We added the results of this analysis in Fig S5, and refer to it in the main text (lines 160-173). Environmental *Pseudomonas* load was negligible (inferred from comparing *Pseudomonas* in infected and control plants), and the load of *Pseudomonas* in infected plants was higher than the cumulative load of all other non-*Pseudomonas* bacteria. Thus, we are confident that background microbiota does not greatly distort our inferences.

2e. In Figure 2 the authors show the effect of the different SynCom treatments across the A.thaliana genotypes. It is clear that the different genotypes exhibit different magnitude of response to the distinct SynComs but all of them exhibit the same shape in the trend. To confirm this, the authors should add an interaction model and discuss this result further. Without the existence of a significant interaction in the model, these results look more like pseudo replication of the phenomenon and therefore requires toning down on the "host-dependent" claim.

Thank you for this suggestion. We have now analyzed the model [weight ~ genotype * treatment + experiment] (i.e., among the two experiments we made), finding a significant importance of the [genotype*treatment] interaction using both (i) leave-one-out, Bayesian-based model comparison and (ii) typical ANOVA test. We added these results in Table S3 (lines 201-204 of the main text). These results validate the general 'host-dependent' claim.

Host-dependency is also apparent from Fig S7B, presenting differences among genotypes after infection with PathoCom and MixedCom, using the model [weight ~ genotype * treatment + experiment], thus considering the two independent experiments. For statistical inference, 95% credible intervals (CIs) are reported.

Lastly, we provide a separate statistical analysis for each of the two experiments (in the revised version: Fig 2 and Fig S8, bottom panels, presenting 95% confidence intervals, as inferred from bootstrapping). In both statistical analyses, Ey15-2 and Kus3-1 were more susceptible to PathoCom, and only Ey15-2 was susceptible to MixedCom. We report such differences also in the text to make it easier for the reader (lines 207-210; lines 221-224).

Collectively, the various statistical analyses are in agreement about how plant genotype affects the susceptibility or resistance/tolerance to PathoCom and MixedCom.

2f. For the RNA-Seq experiment, the authors sampled treated plants at three and four day after infection, nevertheless there is not a clear justification for these overlapping timepoints. Have the microbial communities reached stability at this point?

We wanted to sample early time points in which the infecting bacteria had reached sufficient titers to elicit a plant response, but before the community profile was affected by the host response. In other words, we believe that an early response is a better predictor for subsequent microbial changes, as stated by Nobori et al. (2018): "Expression patterns of immune-responsive bacterial genes at the early time point were tightly linked to later bacterial growth levels in different host genotypes."

We chose two early time points - 3 and 4 days post infection - to make sure that the results were not idiosyncratic, capturing the effects only at an arbitrary time point. That the two time points turned out to be similar supports our design. We do admit, however, that the specific selection of 3/4 days after infection, rather than 2/3 or 4/5 days was arbitrary. Given that we used a system that is not common for plant-microbe RNA-seq studies (non-sterile conditions, soil-grown plants, spray infections), we could not draw on previous literature.

Reviewer #2 (Remarks to the Author):

Summary:
How plants select commensals over pathogens is poorly understood. The manuscript by Shalev et al. shows that commensal bacterial communities and the plant host interact to inhibit bacterial pathogens in the phyllosphere. This work also shows that plants are able to tolerate higher loads of commensals compared to pathogens. This study describes how either specific strains of commensals or commensal communities are able to inhibit pathogen abundance and other commensal strains either by direct bacterial competition or in combination with the host.
Shalev et al. provide clear hypotheses and experiments addressing the interaction between bacterial community members and the plant host inhibiting pathogen growth in the phyllosphere. While the experimentation is clear, I am concerned with the statistical significance of the data, or more specifically, a lack of statistical analysis to support several of the major conclusions. Some of the data presented are from single experimental replicates or show little to no statistical analysis to determine whether the differences between treatments are significant. It would be helpful for the manuscript to show that the conclusions are drawn from meaningful differences between the tested conditions and to highlight replicability. Once addressed, this work provides a clear and exciting look into the complex interactions that occur between host and microbes in establishing a healthy plant microbiome.
Major concerns:

Because Reviewer #2 is generally concerned about the lack of statistics, we start with a high-level introduction of our approach: We primarily employed Bayesian rather than frequentist statistics. This approach is motivated by a current trend of moving beyond the dichotomous p-value, a frequentist parameter which is often misinterpreted and oversimplified by many readers (Wasserstein, Schirm, and Lazar 2019; Amrhein, Greenland, and McShane 2019; Halsey 2019; Ho et al. 2019; Buchinsky and Chadha 2017). Bayesian statistics is a suitable alternative, focusing on the uncertainty of an event. The output of a Bayesian model is the 95% credible intervals (CI) of the mean - a more easily understandable result than a p-value that assumes a specific distribution (Hespanhol et al. 2019): there is a 95% chance that the real average is within the credible intervals. This can be done thanks to computational power, as Bayesian models require extensive simulations.

It has become quite common to visualize the 95% CIs, comparing them to a baseline (e.g., control), as these are the main properties of Bayesian linear models. See, for example, Fig 1 in (Halsey 2019), Fig 2 in (Hespanhol et al. 2019), Fig 5 in (Resano-Mayor et al. 2019), Fig 3 in (Strinella et al. 2020), Fig 2 and 3 in (Gorman et al. 2020), or Fig 1 and 2 in (von Felten et al. 2020). The lack of overlap between the CI and the baseline (in our manuscript marked as a dashed line) is equivalent to $p<0.05$, and considered as a substantial effect. We note that similar visual representations are used in frequentist linear-models, instead of a significance notation '*', to avoid simplification of conclusions (Examples in (Halsey 2019), Fig 2 and 3 in (Barry et al. 2020), and Fig 3 and 4 in (Kormann et al. 2015)).

In the lack of meaningful prior information on distributions (which was the case in our study), Bayesian and frequentist statistics should be in agreement, and it is thus a matter of preference. To demonstrate this, we provide here an alternative frequentist analysis for Fig S7A. We conducted ANOVA and post-hoc Tukey-Kramer HSD using the same data and model [weight ~ treatment * genotype + experiment], appreciating 'treatment' coefficients. Similar conclusions can be drawn (output of Tukey-Kramer HSD for the data of Fig S7A; significant differences in bold):

	**weight change (mg)**	**lower bound (mg)**	**upper bound (mg)**	p_{adj}
**PathoCom-Control**	-48.6	-59.5	-37.7	**2.12E-13 *** **
**CommenCom-Control**	14.5	3.6	25.4	**0.0033 ** **
**MixedCom-Control**	-8.5	-19.4	2.3	0.18
**CommenCom-PathoCom**	63.1	52.3	74	**2.08E-13 *** **
**MixedCom-PathoCom**	40	29.2	50	**2.46E-13 *** **

**MixedCom-CommenCom**	-23	-33.9	-12	**3.19E-07 *****

More similarities can be found in revised Table S3, in which we analyzed different components of the model [weight ~ treatment * genotype + experiment] using both Bayesian and frequentist approaches. Again, very similar conclusions can be drawn.

Although Bayesian tools are becoming more and more common, following improvements in computational power, the reviewer's comments were very appropriate and made us re-examine our statistical approach. This included a consultation with Prof. Fränzi Korner-Nievergelt, head of the Ecological Statistics department in the Swiss Ornithological Institute. It was good that we could convince ourselves -- and hopefully also the reviewer -- that our results withstand proper scrutiny.

Below we provide answers to the specific comments. We hope this is clear now, but if the reviewer feels additional information is required, we would be more than happy to add it.

Figure 2, only shows data from one experimental replicate. Results/conclusions are based off of two replicates and the second one isn't shown. No stats are presented to test whether the host-dependent commensal protection is significant. Please add statistical analysis and show both replicates (the second can be shown in the supplement).

We note that most statistical analyses had already been done on both experiments, using linear models (e.g., Fig S7). We have added visualizations of the results from the second experiment as Fig S8, with references in the main text and legend of Fig 2. We apologize that we did not originally do this. We also added results of an axenic experiment as control (Fig S9), recapitulating the major trends of soil-grown plants (statistics in bottom panels). In particular, we confirmed the specific lack of protection in Ey15-2 (i.e., protection is host-dependent) in Fig S10.

Regarding the statistics of host-dependent commensal protection:

1. We added a general analysis of the host-dependent treatment effect [genotype*treatment], using the model [weight ~ genotype * treatment + experiment] (among all treatments, and the two experiments). We found that the genotype*treatment effect was substantial using both a Bayesian leave-one-out cross-validation, and the frequentist two-way ANOVA (Table S3). Although this is not specific for the MixedCom, it is further evidence for host-dependent weight effects.
2. In both individual experiments (Fig 2 and Fig S8), the bottom panel represents a statistical analysis, comparing the effect of every treatment per genotype (in respect to control treatment). In both experiments, only Ey15-2 presents a substantial decrease in weight after infections with MixedCom (no overlap between 95% confidence intervals with the control baseline, which is identical to the statement $p < 0.05$). Thus in the two analyses, the same conclusion is drawn - only Ey15-2 was not protected by commensals. We also reported the statistical analysis of one experiment in the text (lines 221-224). The interpretation of these two statistical analyses is that commensal protection is host genotype-dependent.
3. In Fig S7 we incorporate the two experiments using the model [weight ~ treatment * genotype + experiment] (using a Bayesian linear model). In Fig S7A we provide a statistical analysis for the weight effect of the three SynComs among the two experiments, regardless of genotype (the 'treatment' coefficients). The overlap between the 95% credible intervals (CIs) of MixedCom with the control baseline denotes that MixedCom infections did not alter plant weight in general. In Fig S7B we constrained the treatment weight effects by genotypes (the 'treatment * genotype' coefficients). It can be concluded from the lack of overlap between the 95% credible intervals (CIs) of Ey15-2 and Lu3-30 with the control baseline (Kus3-1 was arbitrary chosen) that not all genotypes were similarly affected by the MixedCom. Therefore, the MixedCom differentially affects plant genotypes.

Figure 3B, there is a large amount of variance in the regression slopes for each treatment. It would be useful to know the R^2 for each to know how the variance in rosette fresh weight is related to bacterial load. Additionally, the authors should test if the differences in slope are significantly more negative to support the claim that the slope is more negative.

Thank you for the comment. We added the R^2, as well as p-value for each correction in Fig 3B, and mentioned it in the figure legend. Regarding the slope analysis – see the same general comment as above regarding Bayesian statistics. Please see also revised Fig S11A, in which the slope per treatment is statistically tested among the two experiments. We used the linear model model [weight ~ treatment * log10(cumulative isolate load) + genotype + experiment] to estimate the slope [treatment * log10(cumulative isolate load]. Baseline is PathoCom. This information is incorporated in the figure legend. The 95% CIs of CommenCom are not overlapping with PathoCom, noting that the true slopes are different (by more than 95% chance). To better convey the message, we reported the numbers in the text: "CommenCom mean effect difference to PathoCom: 12.0 mg [4.4,19.5], at 95% credible interval of the parameter log10(isolate load) * treatment)" (line 265-270 in the revised version). The fact that the reported numbers are positive for the 95% credible interval indicates that that the slope is more positive. The implication is that for each unit of [log10(isolate load)], the weight reduction in CommenCom is lower by 4.4-19.5 mg (median of 12 mg) in comparison to PathoCom. In other words, every unit of load reduces the weight to a lesser extent in CommenCom compared to PathoCom.

Figure 4B, no stats are available to show which bacteria isolates have loads that are significantly different when compared to the Mixed Community. Similar critique for Figure 4E.

Both Fig 4B and 4E are visualizations of the actual statistical analyses. Again, we examined relevant coefficients for the linear models detailed in the figure legend, and present the 95% CIs with a relevant baseline. Although we do not want to dichotomously say significant or not (as this requires an arbitrary cutoff), but rather use measures of uncertainty, these statistical analyses reflect a Bayesian approach. They do not present only an observed difference from control, but also among the isolates. For example, from Fig 4B it is apparent that P1 and P7 are inhibited in MixedCom, although P1 is inhibited to a greater extent.

Figure 6C, statistical analysis would be useful for determining whether the bacterial isolate P6 has a significant effect on Ey15-2 rosette weight and for judging the changes in abundance in Figure 6B.

For Fig 6C, we employed a package that combines raw data with a statistical analysis based on bootstrapping (Ho et al. 2019). This time it is a frequentist-based approach, showing 95% confidence intervals. These so-called 'Gardner–Altman' plots are rapidly becoming popular (481 citations for Ho et al. (Ho et al. 2019)), and are an up-to-date representation of the data and statistics, presenting a richer picture of variation, rather than reducing the statistical representation into a '*' notation. Again, substantial effects become easily interpretable as lack of overlap between the control baseline and 95% confidence intervals.

Fig 6B is visualization of the actual statistical analysis, similar to Fig 4B, E, Fig S7, etc. We employed the same Bayesian modelling as we did for most analyses in the paper, with a small twist. This time, the 95% credible intervals of P6 are marked by shaded area - thus lack of overlap for 95% CI of other strains with this area is indicative of major abundance differences (there is 95% chance that their real abundance average is within the corresponding CI). Thus, instead of presenting the 'effect size in comparison to control', we present the 95% CIs for each isolate. We find this representation better specifically for this analysis, as one can compare the absolute P6 loads both among the genotypes, and within a genotype, among the other isolates. Thus, with a single plot, one can appreciate how P6 load is not that much more abundant in Ey15-2 *per se*, but more abundant in comparison to the other isolates.

Please describe how abundance of mean difference was calculated in the methods. A reference is included, but this metric is used frequently enough that it would be useful to have a brief description within the methods. Additionally, please define the y-axis in the legends in a bit more detail so the reader has a sense of what the scale means.

Thanks for the suggestions. We added a description of the package dabestr, including more details about the statistics, to the Methods section. Moreover, we added the unit (mg) to the y-axis title. This was indeed missing. Now it is better conveyed that the bottom panel present the mean *weight* difference, in respect to control. Thus, the effect of each treatment*genotype is estimated at 95% confidence interval.

Minor concerns:
Line 99, population should be plural

Changed to 'populations'.

Line 170, change to "explained 4-26% of compositional variation" to mirror a similar statement seen in line 167.

Changed.

Line 138: I don't think "equimolar" is appropriate for pooling bacteria. Do you mean equivalent estimated CFUs/OD600 readings?

Indeed. Thank you for the correction. Changed to: "Isolates were mixed in equal proportions (based on OD600 readout), and their absolute starting concentration was identical in each synthetic community."

Line 139: I think you mean twice the number of cells per unit volume and not just the twice the total number of cells?

Exactly. Thanks. Changed to "bacterial cells per volume".

In some cases more method details are needed within the results to follow how the experiments were done. For instance, lines 235-7 bacterial load was quantified based on barcoded read data, but this isn't specified.

Thank you for this suggestion. We added "as deduced from quantification of barcodes", as suggested. In general, the bacterial load measurements throughout the study rely on our barcoding method. This and other experimental details are specified in the introductory results part "Genome barcoding of *Pseudomonas* isolates and experimental design".

Our concern in specifying such details in too many different places throughout the study is that it would make the flow of the narrative awkward (with many redundancies) and perhaps even mislead the reader to conclude that there is an exception wherever the method is specified (following the principal "unless otherwise mentioned", and in relation to the introductory part). If there are any other places that seem to lack proper references to the methods, please let us know.

Line 206 "on" should be "of"

Changed.

Line 307 "in at least in two" should be "in at least two"

Changed.

Line 328 "in plana" should be "in planta"

Changed.

Many thanks for paying attention to all of these fine details, which greatly improved the text and presentation.

Reviewer #3 (Remarks to the Author):

Dear Editor,
Thank you for inviting me to review the manuscript. The authors Shalev et al., presented a very interesting story of 6 Arabidopsis genotypes interact with different groups of commensal, pathogenic and mixed Pseudomonas synComs on their leaves, and discovered microbe-microbe driven plant phenotypes, and the potential plant recognition mechanism behind. Particularly, the authors presented a nice genome-barcoding method to each Pseudomonas strain, which could facilitate both tracking and quantification of each Pseudomonas isolate. However, some important aspects should be clarified and improved before the paper can be accepted for publication. Therefore, my recommendation for the manuscript would be a moderate revision. Detailed major and minor questions and comments are listed as following:
Major comments:
1. What was the rationale of choosing only Pseudomonas lineages for the CommenCom, but not a more diverse group of different bacteria to mimic more the natural conditions?

One of our main goals was to study interactions at the microbe and host strain-level. Such fine-grained taxonomic resolution makes our study distinct from many other studies. The Karasov et al. (2018) collection reported 1,524 isolates from the same genus, *Pseudomonas*, that co-exist on the same host, *Arabidopsis thaliana*, in a small geographic area. Nonetheless, these isolates have surprisingly high diversity, even among groups with similar 16S rDNA sequences. These genomic differences were reflected by functional differences, as tested in mono-infections (Karasov et al., 2018).

In the current study we focused on this collection of strains, to gain insight into how the diversity of the local *Pseudomonas* population is maintained. Our starting hypothesis was that functional differences in interaction with other *Pseudomonas* strains and with different host genotypes are at least part of the answer. We studied the function of related strains in consortia, competing them *in planta* in the framework of multiple communities. Our results strongly imply different functions among these closely related strains.(e.g., differential competitive qualities of strains among the three synthetic communities, effect on specific plant genotypes as in MixedCom:Ey15-2, etc). Thus, our study provides further evidence that clustering by marker-gene similarity (e.g., 16S rDNA) is insufficient to fully understand microbial functions in wild plant populations.

2. For the three synthetic communities, PathoCom, CommenCom and MixedCom, did the authors test if each strain can grow well within each community? In another word, for instance, within the PathoCom or the CommenCom, did the 7 strains grow equally well when mixing them together, but not certain strain(s) dominate and take over the communities? This is to exclude the possibility of the plant phenotype observed later come from specific strain(s), but not from the communities together (or interactions). How did the authors test this? Please specify and provide the supporting info

Each strain was grown separately. Strains were subsequently diluted and mixed in similar concentrations to construct the three SynComs. It is not likely that bacteria grew after mixing as they were washed and resuspended in 10 mM $MgSO_4$. In other words, we infected plants with communities made of isolates in similar concentrations. This straightforward approach is typical for constructing SynComs (for example, (Carlström et al. 2019)).

3. Line 148, why in Figure S4, Ey15-2 was represented? Do the other cultivars show the similar phenotype as Ey15-2? Line 152, Why choose to inoculate synthetic communities after 21 days of sowing?

Starting with Fig S4: Although Ey15-2 was represented, other plant genotypes showed phenotypes that were broadly similar. In the MS-agar system, but not on soil, infections with the PathoCom completely killed most individual plants (with more resistant genotypes suffering less lethality). On soil, plant death was not observed for any plant genotype. Since the weight correlates well with the phenotype, one can compare the weight of individual plants after PathoCom infection among the plant genotypes (Fig S9 for axenic conditions and Fig 2 for soil-grown plants). Plants with a nearly 0 g weight in axenic conditions indicate dead plants. (None among soil-grown plants.)

We chose to infect soil-grown plants 21 days after sowing for two reasons:

- We used non-sterile conditions because we already knew that the pathogenic *Pseudomonas* strains were not lethal in natural conditions (Karasov et al. 2018), which we sought to mimic here.

- Plants with ~6 leaves had been used for the initial characterization of the *Pseudomonas* collection (Karasov et al. 2018). The 21-day time point was also chosen, because we wanted to sample plants during the growth phase, so we could easily measure reduction in biomass, with plants having a size that made measurement reasonably easy, and finally before plants flowered.

4. Line 193-203, How to rule out the plant phenotype (weight loss) is not due to root microbiome effect (as well) since the authors used non-sterile soil? A suggestion is to add sterile soil treatment which would help to answer this for Figure 2.

Thank you for this suggestion. Indeed, in our original design, we could not exclude that the host effects were indirect and mediated by background microbiota, either in the root or leaf compartment. We there added results from an axenic experiment (MS-agar system) (Fig S9 in the revision). We recapitulated the main effects observed in soil-grown plants, namely, (i) protection by commensals from pathogens in MixedCom-infected plants, and (ii) lack of full protection from pathogens in Ey15-2, indicating that they are independent of background microbiota, the main focus of this study. This does not exclude that environmental microbiota enhances or dampens some of the observed effects, but if they do, they do so in a general manner, and they are not essential for the observed effects.

5. Line 253-255, figure 3B. Is there a significant difference of the regression slopes of PathoCom and CommenCom? It looks like there is overlap which indicates no significant difference. Please clarify the statistical analysis.

The same question was raised by reviewer #2, to which we answered the following:

*"Specifically, please refer to revised Fig S11A, in which the slope per treatment is statistically tested among the two experiments. We used the linear model model [weight ~ treatment * log10(cumulative isolate load) + genotype + experiment] to estimate the slope [treatment * log10(cumulative isolate load]. Baseline is PathoCom. This information is incorporated in the figure legend. The 95% CIs of CommenCom are not overlapping with PathoCom, noting that the true slopes are different (by more than 95% chance). To better convey the message, we reported the numbers in the text: "CommenCom mean effect difference to PathoCom: 12.0 mg [4.4,19.5], at 95% credible interval of the parameter log10(isolate load) * treatment)" (line 265-270 in the revised version). The fact that the reported numbers are positive for the 95% credible interval indicates that that the slope is more positive. The implication is that for each unit of [log10(isolate load)], the weight reduction in CommenCom is lower by 4.4-19.5 mg (median of 12 mg) in comparison to PathoCom. In other words, every unit of load reduces the weight to a lesser extent in CommenCom compared to PathoCom."*

We note that we implemented Bayesian statistics throughout most of this study. It is an alternative to the frequentist approach that normally relies on the simplistic p-value. Since it is computationally heavy, it has rarely been used in the past, but it is becoming more and more common and recommended, especially for studies in ecology and other areas of biology (Wasserstein, Schirm, and Lazar 2019; Amrhein, Greenland, and McShane 2019; Halsey 2019; Ho et al. 2019). For similar analysis and visualizations, see, for example, Fig 1 in (Halsey 2019), Fig 2 in (Hespanhol et al. 2019), Fig 2 in (Resano-Mayor et al. 2019), Fig 3 in (Strinella et al. 2020).

6. Figure 4B. Line 280-282. I didn't understand this statement. Since in Line 278-280, it stated that "pathogenic isolates were compared between PathoCom and MixedCom". Why in Line 281, the statement is the abundance of pathogens was significantly lower in MixedCom? How exactly to interpret figure 4B?

This concern refers to the following complete statement: *"We compared the absolute abundance of each isolate among the treatments: Pathogenic isolates were compared between PathoCom and MixedCom, and commensals between CommenCom and MixedCom. In general, the abundance of pathogens was significantly lower in MixedCom, while the abundance of commensals was either similar or slightly higher in MixedCom."*

The abundance of each of the isolates was compared between the exclusive SyCom (i.e., PathoCom or CommenCom) and the mixed community (i.e., MixedCom). In other words, we wanted to understand how the abundance of each isolate changed when it was mixed with isolates of the other class. For example, all seven pathogens had higher loads in the PathoCom than in MixedCom. Thus, mixing with CommenCom members led to inhibition of PathoCom members. On the other hand, CommenCom members were not suppressed when mixed with PathoCom members, and their abundance has not greatly changed (Fig 4B).

In Fig 4B, the baseline is the level of a given isolate in the exclusive SynCom, and the vertical bars denote the 95% credible intervals of abundance change in the MixedCom. As noted in the figure legend: "Abundance mean difference was estimated with the model log10(isolate load) ~ treatment * experiment + error, for each individual strain. Thus, the treatment coefficient was estimated per isolate". Therefore, we ran a linear model for each strain, predicting how its abundance was affected by the 'treatment' (PathoCom/CommenCom vs. MixedCom). This was done across the two experiments.

7. Line 327-328, it stated that "no commensal isolate was inhibited in planta, among communities". How about C6? what does the negative value indicate in the figure 4B?

Thank you for the question. The interpretation is based on the median of the credible interval (the dot). A substantial change in Bayesian statistics is commonly conveyed by a lack of overlap between the 95% credible interval with the baseline (replacing the p-value <0.05 criterion). We refer to the literature we cited in point #5.

In the case of P6, the baseline (dashed line) denotes the abundance level in CommenCom. Although the median of the 95% credible interval is just below the baseline, as stated by the reviewer, the vertical bars (95% credible intervals) are clearly overlapping the baseline. The interpretation is that there is less than a 95% chance that the real abundance of P6 in MixedCom differs from CommenCom. We conclude that the observed small differences are within the margin of experimental error, as the abundance of P6 is very similar among CommenCom and MixedCom. Such a minor difference would be equivalent to a large p-value in frequentist tests.

8. Line 350, Though pathogen inhibition seemed to be independent of the host genotype, what was the rationale of choosing HE-1 here? Why not choose the most resistant (Lu3-30, TueWal-2) and most susceptible (Ey15-2) plant genotype or Lu3-30 the authors chose in next experiment?

We note that the definition of 'resistant genotypes' is in the context of PathoCom alone. After MixedCom infections, all genotypes were affected similarly (i.e., all were resistant to MixedCom), except for Ey15-2. Thus, to investigate how plant protection occurs in the context of microbe-microbe interactions – in this case studying how individual CommenCom members affect PathoCom – Ey15-2 is not an ideal choice, as it is exceptional due to its lack of protection. To study how protection works, we needed a protectable plant genotype.

The choice among the protectable genotypes (choosing HE-1) was somewhat arbitrary. In the next experiment we used another arbitrarily chosen resistant genotype, Lu3-30, but in a different context – we infected plants with all SynComs, comparing their effects on the plant.

9. Line 362, what was the rationale of choosing the genotype Lu3-30 for the RNA-seq experiment? And what was the rationale of sampling on 3 and 4-dpi, but not 12-dpi, which is consistent with the spray assay for Figure 2?

We start by answering the choice of the genotype Lu3-30: We wanted to pinpoint putative expression dynamics that are related to protection by CommenCom members. Among the questions we aimed to answer were which CommCom-induced genes differ from the PathoCom-induced ones, and are the differences qualitative or quantitative? Lu3-30 was less susceptible to PathoCom (just as Tu-Wal-2). Hence, weight loss was less severe after infection with PathoCom, and we hypothesized that this phenomenon is due to responsiveness to the PathoCom members. Although this turned out as false, the motivation was based on some evidence.

Reviewer #1 was also wondering about the days of sampling. We answered as follows:

"We wanted to sample early time points in which the infecting bacteria had reached sufficient titers to elicit a plant response, but before the community profile was affected by the host response. In other words, we believe that an early response is a better predictor for subsequent microbial changes, as stated by Nobori et al. (2018): 'Expression patterns of immune-responsive bacterial genes at the early time point were tightly linked to later bacterial growth levels in different host genotypes.'

We chose two early time points - 3 and 4 days post infection - to make sure that the results were not idiosyncratic, capturing the effects only at an arbitrary time point. That the two time points turned out to be similar supports our design. We do admit, however, that the specific selection of 3/4 days after infection, rather than 2/3 or 4/5 days was arbitrary. Given that we used a system that is not common for plant-microbe RNA-seq studies (non-sterile conditions, soil-grown plants, spray infections), we could not draw on previous literature."

10. Line 417-419, the marker genes (PR1 and PR5) expression in MixedCom- and CommenCom-infected hosts are very similar, but CommenCom-infected hosts have the most promoted effect on the weight than MixedCom- infected hosts. What could be the reason for this? Does it mean that SAR is not the main or only explanation?

Yes, the CommenCom members were mildly growth promoting. Nonetheless, the PathoCom members produced a much greater effect, about four times as strong, in the opposite direction (Fig S7A). Hence, it is unlikely that growth promotion *per se* led to protection. This is especially true when considering Ey15-2, in which there was no protection (although the CommenCom effect was similar among the plant genotypes). Also, the PathoCom members were highly inhibited in the MixedCom, and this provides a more plausible explanation for the protection effect, as plants were less tolerant to PathoCom members (Fig 3B; revised Fig S11A). Therefore, we associate the suppression of the harmful PathoCom members with the plant immune response. *PR1* and *PR5* are indeed marker genes for SAR, and were induced by exposure to CommenCom members. These results are in agreement with previous studies (e.g., (De Vleesschauwer et al. 2008)). Lastly, the fact that *PR1* and *PR5* expression was similar among MixedCom and CommenCom-infected plants, and higher than in PathoCom-challenged plants, implies that PathoCom members did partially evade detection by the host.

Minor comments:

1. Line 37, "and are dictated by the balance of inhibition and facilitation of growth". It is not clear whether it is for plant or microbial growth or both.

Thanks, indeed unclear. Added "by both the host and other microbes".

2. Line 52-53, There is no logical relationship between "other environmental factors" and "including other resident microbes". Please rephrase.

Changed to "In contrast, abiotic factors [5,6] as well as local reservoirs of microbes have a large influence on the composition of microbial communities [3,14,15]."

3. Line 210, the effect of PathoCom on the genotype Ey15-2 weight should be emphasized in Line 195-198.

In original line 210, infections with MixedCom were discussed. The effect of MixedCom on Ey15-2 was unique – no full protection, unlike in all other plant genotypes. In original lines 195-198, we focused on PathoCom, which did not affect the host genotype Ey15-2 in a unique way. Specifically, weight reduction in Ey15-2 was slightly greater than in Lu3-30 and Tu-Wal-2, but similar to Schl-7 and Kus3-1 (Fig 2; Fig S7; Fig S8).

4. Line 228, the second experiment results should be shown in supplementary info.

Agreed, this was indeed an omission. Visualization of the results from the second experiment has been added as Fig S8. We note that most statistical analyses had already been done on both experiments, using linear models (e.g., Fig S7).

5. Line 342, What do "higher-order interactions" refer here? Please specify.

We added an explanatory sentence to make it clearer: "thus, interactions among pathogens were constrained by the presence of commensals and vice versa" (lines 368-369).

References cited

Amrhein, Valentin, Sander Greenland, and Blake McShane. 2019. "Scientists Rise up against Statistical Significance." *Nature* 567 (7748): 305–7.

Barry, Kathryn E., Jasper van Ruijven, Liesje Mommer, Yongfei Bai, Carl Beierkuhnlein, Nina Buchmann, Hans de Kroon, et al. 2020. "Limited Evidence for Spatial Resource Partitioning across Temperate Grassland Biodiversity Experiments." *Ecology* 101 (1): e02905.

Buchinsky, Farrel J., and Neil K. Chadha. 2017. "To P or Not to P: Backing Bayesian Statistics." *Otolaryngology--Head and Neck Surgery: Official Journal of American Academy of Otolaryngology-Head and Neck Surgery* 157 (6): 915–18.

Carlström, Charlotte I., Christopher M. Field, Miriam Bortfeld-Miller, Barbara Müller, Shinichi Sunagawa, and Julia A. Vorholt. 2019. "Synthetic Microbiota Reveal Priority Effects and Keystone Strains in the Arabidopsis Phyllosphere." *Nature Ecology & Evolution* 3 (10): 1445–54.

De Vleesschauwer, David, Mohammad Djavaheri, Peter A. H. M. Bakker, and Monica Höfte. 2008. "Pseudomonas Fluorescens WCS374r-Induced Systemic Resistance in Rice against Magnaporthe Oryzae Is Based on Pseudobactin-Mediated Priming for a Salicylic Acid-Repressible Multifaceted Defense Response." *Plant Physiology* 148 (4): 1996–2012.

Felten, Stefanie von, Christophe Berney, Bruno Erb, Peter Baumann, Fränzi Korner-Nievergelt, and Beatrice Senn-Irlet. 2020. "Habitat Enhancements for Reptiles in a Beech Forest May Increase Fungal Species Richness." *Biodiversity and Conservation* 29 (6): 1805–19.

Gloor, Gregory B., Jean M. Macklaim, Vera Pawlowsky-Glahn, and Juan J. Egozcue. 2017. "Microbiome Datasets Are Compositional: And This Is Not Optional." *Frontiers in Microbiology* 8 (November): 2224.

Gorman, Courtney E., Lindsay Bond, Mark van Kleunen, Marcel E. Dorken, and Marc Stift. 2020. "Limited Phenological and Pollinator-Mediated Isolation among Selfing and Outcrossing Arabidopsis Lyrata Populations." *Proceedings. Biological Sciences / The Royal Society* 287 (1939): 20202323.

Halsey, Lewis G. 2019. "The Reign of the P-Value Is over: What Alternative Analyses Could We Employ to Fill the Power Vacuum?" *Biology Letters* 15 (5): 20190174.

Hespanhol, Luiz, Caio Sain Vallio, Lucíola Menezes Costa, and Bruno T. Saragiotto. 2019. "Understanding and Interpreting Confidence and Credible Intervals around Effect Estimates." *Brazilian Journal of Physical Therapy* 23 (4): 290–301.

Ho, Joses, Tayfun Tumkaya, Sameer Aryal, Hyungwon Choi, and Adam Claridge-Chang. 2019. "Moving beyond P Values: Data Analysis with Estimation Graphics." *Nature Methods* 16 (7): 565–66.

Karasov, Talia L., Juliana Almario, Claudia Friedemann, Wei Ding, Michael Giolai, Darren Heavens, Sonja Kersten, et al. 2018. "Arabidopsis Thaliana and Pseudomonas Pathogens Exhibit Stable Associations over Evolutionary Timescales." *Cell Host & Microbe* 24 (1): 168–79.e4.

Kormann, Urs, Verena Rösch, Péter Batáry, Teja Tscharntke, Kirill Márk Orci, Ferenc Samu, and Christoph Scherber. 2015. "Local and Landscape Management Drive Trait-Mediated Biodiversity of Nine Taxa on Small Grassland Fragments." *Diversity & Distributions* 21 (10): 1204–17.

Lundberg, Derek S., Pratchaya Pramoj Na Ayutthaya, Annett Strauß, Gautam Shirsekar, Wen-Sui Lo, Thomas Lahaye, and Detlef Weigel. 2020. "Host-Associated Microbe PCR (hamPCR): Accessing New Biology through Convenient Measurement of Both Microbial Load and Community Composition." *Cold Spring Harbor Laboratory*. https://doi.org/10.1101/2020.05.19.103937.

Resano-Mayor, Jaime, Fränzi Korner-Nievergelt, Sergio Vignali, Nathan Horrenberger, and Raphaël Arlettaz. 2019. "Snow Cover Phenology Is the Main Driver of Foraging Habitat Selection for a High-Alpine Passerine during Breeding: Implications for Species Persistence in the Face of Climate Change." *Biodiversity and Conservation* 28 (10). https://doi.org/10.1007/s10531-019-01786-9.

Strinella, Eliseo, Davide Scridel, Mattia Brambilla, Christian Schano, and Fränzi Korner-Nievergelt. 2020. "Potential Sex-Dependent Effects of Weather on Apparent Survival of a High-Elevation Specialist." *Scientific Reports* 10 (1): 8386.

Wasserstein, Ronald L., Allen L. Schirm, and Nicole A. Lazar. 2019. "Moving to a World Beyond 'p < 0.05.'" *The American Statistician* 73 (sup1): 1–19.

natureresearch

Decision Letter, first revision:

11th August 2021

*Please ensure you delete the link to your author homepage in this e-mail if you wish to forward it to your co-authors.

Dear Dr Weigel,

Your manuscript entitled "Protective host-dependent antagonism among <i>Pseudomonas</i> in the <i>Arabidopsis</i> phyllosphere" has now been seen again by the three original reviewers, as well as by the new Reviewer 4 who looked at the Bayesian analyses. Their whose comments are copied below. While Reviewers 1 and 2 now endorse publication, Reviewer 3 has a comment that may still need to be addressed with a supplementary analysis, and Reviewer 4 has some relatively minor suggestions for the statistical reporting.
Therefore we would like to see your responses to the criticisms raised, along with a revised manuscript, before we can reach a final decision regarding publication. Although we do not anticipate that we will need to approach the reviewers again, we may do so depending on the nature of the response.

We therefore invite you to revise your manuscript taking into account all reviewer comments. Please highlight all changes in the manuscript text file [OPTIONAL: in Microsoft Word format].

We are committed to providing a fair and constructive peer-review process. Do not hesitate to contact us if there are specific requests from the reviewers that you believe are technically impossible or unlikely to yield a meaningful outcome.

When revising your manuscript:

* Include a “Response to reviewers” document detailing, point-by-point, how you addressed each reviewer comment. If no action was taken to address a point, you must provide a compelling argument. This response will be sent back to the reviewers along with the revised manuscript.

* If you have not done so already please begin to revise your manuscript so that it conforms to our Article format instructions at http://www.nature.com/natecolevol/info/final-submission. Refer also to any guidelines provided in this letter.

* Include a revised version of any required reporting checklist. It will be available to referees (and, potentially, statisticians) to aid in their evaluation if the manuscript goes back for peer review. A revised checklist is essential for re-review of the paper.

Please use the link below to submit your revised manuscript and related files:

[REDACTED]

Note: This URL links to your confidential home page and associated information

about manuscripts you may have submitted, or that you are reviewing for us. If you wish to forward this email to co-authors, please delete the link to your homepage.

We hope to receive your revised manuscript within four to eight weeks. If you cannot send it within this time, please let us know. We will be happy to consider your revision so long as nothing similar has been accepted for publication at Nature Ecology & Evolution or published elsewhere.

Nature Ecology & Evolution is committed to improving transparency in authorship. As part of our efforts in this direction, we are now requesting that all authors identified as 'corresponding author' on published papers create and link their Open Researcher and Contributor Identifier (ORCID) with their account on the Manuscript Tracking System (MTS), prior to acceptance. ORCID helps the scientific community achieve unambiguous attribution of all scholarly contributions. You can create and link your ORCID from the home page of the MTS by clicking on 'Modify my Springer Nature account'. For more information please visit please visit www.springernature.com/orcid.

Please do not hesitate to contact me if you have any questions or would like to discuss these revisions further.

We look forward to seeing the revised manuscript and thank you for the opportunity to review your work.

[REDACTED]

Reviewers' comments:

Reviewer #1 (Remarks to the Author):

The authors did a very good job at going through all my points raised and clarifying them or providing further evidence to support their claims. I particularly appreciate the gnotobiotic experiment performed and the incorporation of the Other microbiota model.

Reviewer #2 (Remarks to the Author):

I thank the authors for the thoughtful and thorough primer on Bayesian statistics and clarifications to the questions and concerns I raised. I appreciate the authors care in their response, from which I learned a great deal. I believe the authors have fully addressed my concerns about the statistics, as well as the minor concerns I raised previously.

I have also read the responses to other reviewers and believe the authors addressed their comments and concerns as well.

Reviewer #3 (Remarks to the Author):

The authors have addressed most of the important aspects with impressive efforts.
There is only one question regarding my previous point 2, the mix of three synthetic communities. The authors explained very clearly that they washed, diluted, resuspended each strain in 10mM $MgSO_4$ and mixed similar concentrations of each strain to construct the three SynComs, and provided a nice reference as well. Still, my question concerns not about if the strains were growing together beforehand, but more when mixing them and sprayed on Arabidopsis, within each SynCom, are the colonization ability/survival rates of each strain similar? Since the authors barcode all the strains, it should be easy to quantify the relative and also absolute abundance of each strain within each SynCom at specific dpi (such as at 12dpi when the rosette fresh weight was measured), to rule out the plant phenotype observed is from specific dominant colonizing strain(s). This is a general question for all the SynCom studies in the field, especially with a very large number of SynCom above hundreds. Ruling out the phenotype observed is from certain dominating strains due to internal competition/antagonism.

Reviewer #4 (Remarks to the Author):

This report is focused on the statistical analyses, their description, and presentation, including the use of Bayesian methods for the regression models. The manuscript employs Bayesian model fitting using the R package "Stan," which implements Hamiltonian Monte Carlo for posterior sampling. Model comparison is performed using a Leave-one-out cross-validation (LOO) approach that evaluates the difference of a measure of predictive accuracy between two competing models.

Overall, the use of Bayesian techniques and the description of the results stemming from the application of Bayesian methods appear technically adequate. However, there are a few minor instances where some more care - especially in wording - is needed.
Bayesian methods seem to be motivated by the willingness to avoid the use of statistical significance thresholds (p-values) more than by a practical necessity, e.g., to incorporate prior information. Due to the relatively large sample sizes (n=170; for PathoCom; n=151 for CommenCom; n=182 for MixedCom; n=77-94 for the six A. thaliana genotypes) and due to the use of default priors, inference based on a frequentist or a Bayesian approach should lead to similar results. The authors also discussed this point in their response to the previous round of referee reports. Bayesian methods may also better describe the uncertainty of the decisions.

A few major and minor comments are reported below:

1) Not all analyses have been conducted using a Bayesian approach. This may possibly lead to some confusion for the readers. While I understand that some frequentist analyses are characteristic of the literature in statistical ecology (e.g., Table 1), other analyses could have been performed using a Bayesian approach. See, for example, the plant weight analysis in Figures 2 and S8-S10. The need to take into account non-gaussianity may still lead to a preference for the non-parametric bootstrap-based analysis. However, at the very least and for clarity, I suggest the authors add a qualifier wherever a Bayesian analysis is performed. For example, "Dots indicate the posterior medians and vertical lines the 95% credible intervals of the fitted parameter using a Bayesian approach". Or, equivalently, "Dots indicate the posterior medians and vertical lines the 95% Bayesian credible intervals of the fitted parameter".

2) every time a regression formula is used, the authors appear to follow the R "formula" convention. For example, according to this convention, in the caption of Figure 4,

\log _{10} (isolate load) ~ treatment * experiment+ error

should indicate the regression model with both main effects and interactions between treatment and experiment, i.e.

\log _{10} (isolate load) =\beta_0 + \beta_1 treatment+ \beta_2 experiment + \beta_3 treatment * experiment+ error

I am wondering if the R convention will be clear to the readers of the journal. In essence, many of the model comparisons compare models differing by the presence of a single predictor. I would suggest not to follow the R convention, and simply write the equation out. For the example above:

\log _{10} (isolate load) ~ treatment + experiment + treatment * experiment+ error

As a side note, if the authors didn't follow the R convention and the models are as stated, the authors need to justify the lack of the main effect when an effect modification term is added to the model.

3) Figure 4B and 4E (and Figures S3, S5, S6, S11, and S12); Figure 6 and Figure S16:

Comparisons are conducted between the regression coefficients in independent regressions of the isolates vs several types of predictors. The hypothesis testing is conducted by assessing the overlap of each coefficient's 95% credible interval with zero or another coefficient's 95% credible interval (depending on the context). That is a convenient shortcut to conduct hypothesis testing in a Bayesian setup, and indeed it is mainly OK with unimodal posterior densities. More in general, hypothesis testing in a Bayesian setup is conducted by assessing the posterior probability of the null hypothesis. For example, in Figure 4B the posterior probability that the coefficient for the regression of the isolates C7 is different than zero is likely quite high and close to 0.95. Similarly, for C5. The appropriate threshold of the posterior probability can be chosen by controlling a Bayesian FDR threshold to address the multi-comparison problem. In this context, the overlap criterion is probably acceptable. I also recognize that it is a criterion often used in practice. I did not see obvious cases where the results would have been substantially different from those stated (except perhaps for the two cases mentioned above in Figure 4B). However, the authors may want to check and clarify this point in the Methods section.

Minor Comments:

a) As noted above, the word "medians" should be more precisely substituted by "posterior medians". I understand the word "posterior median" may be confusing to a reader. An alternative may be to indicate "median estimates" in the text and then clarify in the Methods section that median estimates are indeed posterior median in a Bayesian setting. A related point is the following: why do the authors use the posterior median instead of the posterior mean? For the type of posterior distributions they consider, it should not make a big difference. However, posterior means are typically more common. Posterior means are the optimal estimators under a quadratic loss function, whereas posterior medians are the optimal estimators under a L_1 (absolute value) loss.

b) l. 185 "For comparison, the batch effect (between the different experiments)"

Typically, in genomics, the batch effect does not refer to comparisons between experiments, but to technical sources of variation between samples even within a single type of experiment. Please, clarify

c) l. 260-263 "indicating that weight can be better predicted by load within a treatment than by load among treatments (difference in expected log scaled predictive density = -52.9...see Methods)".

I have a few problems with this paragraph. First, as stated, the model

weight ~ treatment * log10(isolate load) + genotype + experiment + error

includes an effect modification without the main effect (see also point 2 above). The model appears to be compared with the model:

weight ~ log10(isolate load) + genotype + experiment + error,

which does not include a main effect of treatment.

Hence, the explanation on lines 259-260 that "weight can be better predicted by load within a treatment than by load among treatments" appears difficult to justify in this context. The interpretation of the interaction terms always needs to be reported carefully. The lack of an interaction term means that there is no modification of the main effect due to the isolate load by experiment type, or viceversa there is no modification of the main effect of the experiment due to the isolate load. Comparing the two models as stated does not appear adequate.

Finally, the comparison is made through the LOO criterion.

However, I believe that explicitly reporting the "difference in expected log scaled predictive density" could be confusing to a reader. I would suggest reporting simply "Delta Elpd=-52.9, standard error =9.4) and then define Delta Elpd in the Methods section.

d) l. 289 (Figure 3B) "Correlation of log_10 (bacterial load)"; l. 1123: "Correlation of log10(cumulative isolate load) (Figure S11B)"

Could "association" be a better word? More specifically, it is unclear how these pictures show correlations, as they look like regression lines. Please, clarify.

It is also not clear if a frequentist or Bayesian analysis has been conducted. The text (lines 267 and 273) suggests a Bayesian analysis throughout, whereas the caption discusses a frequentist analysis. If the latter is true, it is not clear why the authors did not conduct a Bayesian analysis in this case.

e) l. 305 "significantly lower"; l. 377: "significantly suppressed", l. 464: "was not significant":

I would avoid the use of the term "significant" for results stemming from a Bayesian analysis since the

term “significant” is strictly related to the “significance level” (p-value) framework. Typical substitutions used in the Bayesian literature include “relevant” or “with high probability”.

f) l. 774: By default, all rstanarm modeling functions - including stan_glm - will run four randomly initialized Markov chains, each for 2,000 iterations (including a warmup period of 1,000 iterations that is discarded). On the contrary, lmBF has a default of 10,000 iterations. The authors should note this point in the Methods section. It would actually be preferable if all the models were run using the same functon(stan_glm) and with the same number of iterations. Also, how was the convergence of the MCMC assessed?

g) l. 774: related to the point above, the authors mention the use of the lmBF package. This is a package for the computation of Bayes factors for specific linear models against the intercept-only null. I did not see the use of Bayes Factors in the main text. Hence, I wonder why the authors require lmBF and how the results have been reported when using that package.

h) l. 781: “This Bayesian-based model comparison provides an estimate for the importance of a predictor in explaining the data” That is not exactly correct. This Bayesian-based model comparison provides an assessment of the prediction accuracy of a model versus a model where a specific predictor has not been included

i) l. 782-783: “Leave-one-out cross-validation improves the estimate in comparison to the common Akaike Information Criterion (AIC) and Deviance Information Criterion (DIC)”. This wording is imprecise. Leave-one-out cross-validation has been shown to improve model selection in comparison to the common Akaike Information Criterion (AIC) and Deviance Information Criterion (DIC). An additional advantage of LOO methods is that it is possible to obtain approximate standard errors for estimated predictive errors and for comparing predictive errors between two models.

l) Figure S3: are these posterior means (as reported) or posterior medians? See also point (a).

*******************END********************

Author Rebuttal, first revision:

NATECOLEVOL-210413396
Second revision

Reviewer #3 (Remarks to the Author):

The authors have addressed most of the important aspects with impressive efforts.
There is only one question regarding my previous point 2, the mix of three synthetic communities. The authors explained very clearly that they washed, diluted, resuspended each strain in 10mM MgSO4 and mixed similar concentrations of each strain to construct the three SynComs, and provided a nice reference as well. Still, my question concerns not about if the strains were growing together beforehand, but more when mixing them and sprayed on Arabidopsis, within each SynCom, are the colonization ability/survival rates of each strain similar? Since the authors barcode all the strains, it should be easy to quantify the relative and also absolute abundance of each strain within each SynCom at specific dpi (such as at 12dpi when the rosette fresh weight was measured), to rule out the plant phenotype observed is from specific dominant colonizing strain(s). This is a general question for all the SynCom studies in the field, especially with a very large number of SynCom above hundreds.
Ruling out the phenotype observed is from certain dominating strains due to internal competition/antagonism.

We are glad that we could address the majority of the concerns! Thank you also for clearing up our misunderstanding of your point 2. We agree that it must be considered that potentially some strains did not manage to establish in the plant due to early competition. We therefore analyzed the data as you suggested, and present the results in new Figure S12. We found that all 14 strains had robustly colonized the infected plants after 12 days, albeit of course to different extents (Figure S12A). Thus, it does not look like a few dominant strains sweeping the rest is a major explanation for our results (Figure S12B).

Reviewer #4 (Remarks to the Author):

This report is focused on the statistical analyses, their description, and presentation, including the use of Bayesian methods for the regression models. The manuscript employs Bayesian model fitting using the R package "Stan," which implements Hamiltonian Monte Carlo for posterior sampling. Model comparison is performed using a Leave-one-out cross-validation (LOO) approach that evaluates the difference of a measure of predictive accuracy between two competing models.

Overall, the use of Bayesian techniques and the description of the results stemming from the application of Bayesian methods appear technically adequate. However, there are a few minor instances where some more care - especially in wording - is needed.
Bayesian methods seem to be motivated by the willingness to avoid the use of statistical significance thresholds (p-values) more than by a practical necessity, e.g., to incorporate prior information. Due to the relatively large sample sizes (n=170; for PathoCom; n=151 for CommenCom; n=182 for MixedCom; n=77-94 for the six A. thaliana genotypes) and due to the use of default priors, inference based on a frequentist or a Bayesian approach should lead to similar results. The authors also discussed this point in their response to the previous round of referee reports. Bayesian methods may also better describe the uncertainty of the decisions.

A few major and minor comments are reported below:

1) Not all analyses have been conducted using a Bayesian approach. This may possibly lead to some confusion for the readers. While I understand that some frequentist analyses are characteristic of the literature in statistical ecology (e.g., Table 1), other analyses could have been performed using a Bayesian approach. See, for example, the plant weight analysis in Figures 2 and S8-S10. The need to take into account non-gaussianity may still lead to a preference for the non-parametric bootstrap-based analysis. However, at the very least and for clarity, I suggest the authors add a qualifier wherever a Bayesian analysis is performed. For example, "Dots indicate the posterior medians and vertical lines the 95% credible intervals of the fitted parameter using a Bayesian approach". Or, equivalently, "Dots indicate the posterior medians and vertical lines the 95% Bayesian credible intervals of the fitted parameter".

Thank you for the important comment. We changed the text exactly as suggested whenever a Bayesian analysis was conducted. To be on the safe side and to avoid any confusion whatsoever, we used the term 'Bayesian credible intervals' instead of 'credible intervals' throughout in all legends.

2) every time a regression formula is used, the authors appear to follow the R "formula" convention. For example, according to this convention, in the caption of Figure 4,

\log _{10} (isolate load) ~ treatment * experiment+ error

should indicate the regression model with both main effects and interactions between treatment and experiment, i.e.

\log _{10} (isolate load) =\beta_0 + \beta_1 treatment+ \beta_2 experiment + \beta_3 treatment * experiment+ error

I am wondering if the R convention will be clear to the readers of the journal. In essence, many of the model comparisons compare models differing by the presence of a single predictor. I would suggest not to follow the R convention, and simply write the equation out. For the example above:

\log _{10} (isolate load) ~ treatment + experiment + treatment * experiment+ error

As a side note, if the authors didn't follow the R convention and the models are as stated, the authors need to justify the lack of the main effect when an effect modification term is added to the model.

As you suspected, we used the R 'formula' convention, which, while compact, is indeed less clear. We changed all equations to the full and clearer form, i.e., wherever the model included interactions we added the notation of each component separately, as suggested.

3) Figure 4B and 4E (and Figures S3, S5, S6, S11, and S12); Figure 6 and Figure S16:

Comparisons are conducted between the regression coefficients in independent regressions of the isolates vs several types of predictors. The hypothesis testing is conducted by assessing the overlap of each coefficient's 95% credible interval with zero or another coefficient's 95% credible interval (depending on the context). That is a convenient shortcut to conduct hypothesis testing in a Bayesian setup, and indeed it is mainly OK with unimodal posterior densities. More in general, hypothesis testing in a Bayesian setup is conducted by assessing the posterior probability of the null hypothesis. For

example, in Figure 4B the posterior probability that the coefficient for the regression of the isolates C7 is different than zero is likely quite high and close to 0.95. Similarly, for C5. The appropriate threshold of the posterior probability can be chosen by controlling a Bayesian FDR threshold to address the multi-comparison problem. In this context, the overlap criterion is
probably acceptable. I also recognize that it is a criterion often used in practice. I did not see obvious cases where the results would have been substantially different from those stated (except perhaps for the two cases mentioned above in Figure 4B). However, the authors may want to check and clarify this point in the Methods section.

We very much appreciate that you approve of the general approach. This particular comment is indeed important. As suggested, we explicitly added in Methods that the overlap between a coefficient's 95% credible interval with a control baseline (zero) or with another coefficient's 95% credible interval was used as an indication for hypothesis testing. We also state explicitly now that we considered not only the binary overlap/nonoverlap status which was used, but also the magnitude of difference, respecting the uncertainty of the real population average, and the measured values.

Regarding the comment about isolates C7 and C5 in Figure 4B - we had also noted that these commensal strains were probably enriched in the MixedCom, since their 95% credible intervals are likely different from zero (higher, in this case). Nonetheless, since this is a borderline case, and these trends are far from our main thrust, we did not highlight them in the text. For the sake of readability, and to be as conservative as possible, we mentioned only strong and relevant trends in the text. Nevertheless, an actual enrichment of C7 and C5 in the MixedCom would only further support our claim that commensal strains dominated the MixedCom. Note that we already provide general evidence that commensals dominated MixedCom-infected plants (Figure 4A). The main focus of Figure 4B is pathogen suppression, a phenomenon which is hopefully very clear.

Minor Comments:

a) As noted above, the word "medians" should be more precisely substituted by "posterior medians". I understand the word "posterior median" may be confusing to a reader. An alternative may be to indicate "median estimates" in the text and then clarify in the Methods section that median estimates are indeed posterior median in a Bayesian setting. A related point is the following: why do the authors use the posterior median instead of the posterior mean? For the type of posterior distributions they consider, it should not make a big difference. However, posterior means are typically more common. Posterior means are the optimal estimators under a quadratic loss function, whereas posterior medians are the optimal estimators under a L_1 (absolute value) loss.

We followed your suggestion and used the term 'median estimates' instead of 'medians'. We also added complementary text in the Methods section to explain that this simplified term refers to the posterior distribution.

Regarding your question - we used the median rather than the mean of the posterior distribution so we will not have to assume a normal distribution. Note that use of the median is the default in the *stan_glm* function we used (R package 'rstanarm'). As you mentioned, the median and mean should be quite similar considering a non-skewed distribution, resulting from the default priors we used. To be on the safe side, we re-examined the median vs. mean, and found a very marginal difference between the two to the point where we can safely say that differences are negligible, in the 4th to 6th decimal in a 2-3 digit value. Of higher importance is that this is only a representation of centrality, and not used for

hypothesis testing and inference. As you noted above, we mainly focused on the overlap or non-overlap between 95 CIs rather than the median value itself.

b) l. 185 "For comparison, the batch effect (between the different experiments)"

Typically, in genomics, the batch effect does not refer to comparisons between experiments, but to technical sources of variation between samples even within a single type of experiment. Please, clarify

Noted and corrected. Thanks.

c) l. 260-263 "indicating that weight can be better predicted by load within a treatment than by load among treatments (difference in expected log scaled predictive density = -52.9...see Methods)".

I have a few problems with this paragraph. First, as stated, the model

weight ~ treatment * log10(isolate load) + genotype + experiment + error

includes an effect modification without the main effect (see also point 2 above). The model appears to be compared with the model:

weight ~ log10(isolate load) + genotype + experiment + error,

which does not include a main effect of treatment.

Hence, the explanation on lines 259-260 that "weight can be better predicted by load within a treatment than by load among treatments" appears difficult to justify in this context. The interpretation of the interaction terms always needs to be reported carefully. The lack of an interaction term means that there is no modification of the main effect due to the isolate load by experiment type, or viceversa there is no modification of the main effect of the experiment due to the isolate load. Comparing the two models as stated does not appear adequate.

Finally, the comparison is made through the LOO criterion.

However, I believe that explicitly reporting the "difference in expected log scaled predictive density" could be confusing to a reader. I would suggest reporting simply "Delta Elpd=-52.9, standard error =9.4) and then define Delta Elpd in the Methods section.

First, we corrected the notation of all models as you suggested in point 2 (adding the main effects). Many thanks also for pointing out the poor phrasing that seemed to imply that we compared other models than what we did. We wrote that we compared the first model to the second "without the treatment factor", when it was actually without the interaction factor [treatment * log10(isolate load)]. We corrected the text accordingly. Thus we compared:

[weight ~ treatment * log10(isolate load) + treatment + log10(isolate load) + genotype + experiment + error]

with

[weight ~ treatment + log10(isolate load) + genotype + experiment + error]

, leaving only the [treatment * log10(isolate load)] factor out.

Lasty, we took your advice and changed the text and Methods accordingly to make the text more readable.

d) l. 289 (Figure 3B) "Correlation of log_10 (bacterial load)"; l. 1123: "Correlation of log10(cumulative isolate load) (Figure S11B)"

Could "association" be a better word? More specifically, it is unclear how these pictures show correlations, as they look like regression lines. Please, clarify.

It is also not clear if a frequentist or Bayesian analysis has been conducted. The text (lines 267 and 273) suggests a Bayesian analysis throughout, whereas the caption discusses a frequentist analysis. If the latter is true, it is not clear why the authors did not conduct a Bayesian analysis in this case.

We changed 'correlation' to 'association' as suggested.

We did both analyses - Bayesian and frequentist. The Bayesian analysis was used for hypothesis testing and inference, while the frequentist was used for initial representation (i.e., to visualize the regression confidence interval in shades and to present a simple R^2 value, as requested by reviewer #2). The more extensive Bayesian analysis for Figure 3B can be found in a separate supplementary - Figure S11. We added references to Figure S11A in lines 267 and 273, to avoid confusion. The data analysis should thus be covered from multiple angles.

e) l. 305 "significantly lower"; l. 377: "significantly suppressed", l. 464: "was not significant":

I would avoid the use of the term "significant" for results stemming from a Bayesian analysis since the term "significant" is strictly related to the "significance level" (p-value) framework. Typical substitutions used in the Bayesian literature include "relevant" or "with high probability".

This is an important commen; thank you. We corrected the text as suggested.

f) l. 774: By default, all rstanarm modeling functions - including stan_glm - will run four randomly initialized Markov chains, each for 2,000 iterations (including a warmup period of 1,000 iterations that is discarded). On the contrary, lmBF has a default of 10,000 iterations. The authors should note this point in the Methods section. It would actually be preferable if all the models were run using the same functon(stan_glm) and with the same number of iterations. Also, how was the convergence of the MCMC assessed?

We changed the Methods section by reporting that we used the default amount of iterations in both functions. We also reported the default values.

We used *lmBF* followed by *posterior* (both are from the same package - 'BayesFactor') in only two analyses: Figure 6B and the related Figure S16. The function *posterior* sample from the posterior distribution of a specified model, thus in principle should give similar results as *stan_glm*. We used 10,000 iterations for the *posterior* function as suggested by a tutorial for the package. We now explicitly mention these details both in the Method section and the relevant figures. We used these functions in Figure 6B and Figure S16 because we did not want to 'lose' a level that will be used as a baseline (meaning, an arbitrary strain in which its bacterial load will be used as the intercept). Specifically, we found it important in these two plots to present the load of all strains, and at the same time be able to compare them. Our approach of *lmBF* followed by *posterior* is designated exactly for this purpose. It provides the 95% CIs of the posterior distributions for all strains, without comparing them to an arbitrary control. We are aware that it should be possible to recover the *stan_glm* output to fit a similar output, but we did not find it as a trivial task. It is important to mention that we also ran *stan_glm* in these two analyses and compared the results as a sanity check. We even tried using different strains as the baseline in *stan_glm*, including the focal strain P6 (although it should not change the results, it may be sometimes confusing using one strain as a baseline rather than another). Notably, we found no differences between the results of *stan_glm* and *lmBF+posterior*, besides the obvious visual differences after losing one level of a factor.

Lastly, we used the Rhat to assess MCMC convergence. With no exception, we reached values between the range 0.99 and 1.01 (mostly much closer to 1), thus in the accepted range.

g) l. 774: related to the point above, the authors mention the use of the lmBF package. This is a package for the computation of Bayes factors for specific linear models against the intercept-only null. I did not see the use of Bayes Factors in the main text. Hence, I wonder why the authors require lmBF and how the results have been reported when using that package.

Please see our answer to point (f).

In short, the *lmBF* function was used to model the data, and the *posterior* function (also from the BayesFactor package) was used to sample from the posterior distribution of that model. Notably, the Bayes factors for the linear model against the intercept-only null, as calculated by the *lmBF* function, were not used in our analyses.

h) l. 781: "This Bayesian-based model comparison provides an estimate for the importance of a predictor in explaining the data" That is not exactly correct. This Bayesian-based model comparison provides an assessment of the prediction accuracy of a model versus a model where a specific predictor has not been included

Thanks for this correction. We corrected the text accordingly.

i) l. 782-783: "Leave-one-out cross-validation improves the estimate in comparison to the common Akaike Information Criterion (AIC) and Deviance Information Criterion (DIC)". This wording is imprecise. Leave-one-out cross-validation has been shown to improve model selection in comparison to the common Akaike Information Criterion (AIC) and Deviance Information Criterion (DIC). An additional advantage of LOO methods is that it is possible to obtain approximate standard errors for estimated predictive errors and for comparing predictive errors between two models.

We followed this comment as well, and corrected the text.

l) Figure S3: are these posterior means (as reported) or posterior medians? See also point (a).

This is the median. We wrote mean by mistake. Thanks for noting all these fine details!

natureresearch

Decision Letter, second revision:

5th November 2021

Dear Dr. Weigel,

I am writting in the temporary absence of my colleague, Dr. Alexa McKay.

Thank you for submitting your revised manuscript "Protective host-dependent antagonism among <i>Pseudomonas</i> in the <i>Arabidopsis</i> phyllosphere" (NATECOLEVOL-210413396B). We have checked the revised manuscript and your responses carefully and we'll be happy in principle to publish it in Nature Ecology & Evolution, pending minor revisions to comply with our editorial and formatting guidelines.

We are now performing detailed checks on your paper and will send you a checklist detailing our editorial and formatting requirements. Alexa is away until the 15th of November, so it will be a few days before you hear from us again. Meanwhile, please email us a copy of the file in an editable format (Microsoft Word or LaTex)-- we can not proceed with PDFs at this stage.

Please do not upload the final materials and make any revisions until you receive this additional information from us.

Thank you again for your interest in Nature Ecology & Evolution. Please do not hesitate to contact me if you have any questions.

[REDACTED]

Our ref: NATECOLEVOL-210413396B

17th November 2021

Dear Dr. Weigel,

Thank you for your patience as we've prepared the guidelines for final submission of your Nature Ecology & Evolution manuscript, "Protective host-dependent antagonism among <i>Pseudomonas</i>

in the <i>Arabidopsis</i> phyllosphere" (NATECOLEVOL-210413396B). Please carefully follow the step-by-step instructions provided in the attached file, and add a response in each row of the table to indicate the changes that you have made. Please also check and comment on any additional marked-up edits we have proposed within the text. Ensuring that each point is addressed will help to ensure that your revised manuscript can be swiftly handed over to our production team.

We would like to start working on your revised paper, with all of the requested files and forms, as soon as possible (preferably within two weeks). Please get in contact with us immediately if you anticipate it taking more than two weeks to submit these revised files.

When you upload your final materials, please include a point-by-point response to any remaining reviewer comments.

If you have not done so already, please alert us to any related manuscripts from your group that are under consideration or in press at other journals, or are being written up for submission to other journals (see: https://www.nature.com/nature-research/editorial-policies/plagiarism#policy-on-duplicate-publication for details).

In recognition of the time and expertise our reviewers provide to Nature Ecology & Evolution's editorial process, we would like to formally acknowledge their contribution to the external peer review of your manuscript entitled "Protective host-dependent antagonism among <i>Pseudomonas</i> in the <i>Arabidopsis</i> phyllosphere". For those reviewers who give their assent, we will be publishing their names alongside the published article.

Nature Ecology & Evolution offers a Transparent Peer Review option for new original research manuscripts submitted after December 1st, 2019. As part of this initiative, we encourage our authors to support increased transparency into the peer review process by agreeing to have the reviewer comments, author rebuttal letters, and editorial decision letters published as a Supplementary item. When you submit your final files please clearly state in your cover letter whether or not you would like to participate in this initiative. Please note that failure to state your preference will result in delays in accepting your manuscript for publication.

Cover suggestions

As you prepare your final files we encourage you to consider whether you have any images or illustrations that may be appropriate for use on the cover of Nature Ecology & Evolution.

Covers should be both aesthetically appealing and scientifically relevant, and should be supplied at the best quality available. Due to the prominence of these images, we do not generally select images featuring faces, children, text, graphs, schematic drawings, or collages on our covers.

We accept TIFF, JPEG, PNG or PSD file formats (a layered PSD file would be ideal), and the image should be at least 300ppi resolution (preferably 600-1200 ppi), in CMYK colour mode.

If your image is selected, we may also use it on the journal website as a banner image, and may need to make artistic alterations to fit our journal style.

Please submit your suggestions, clearly labeled, along with your final files. We'll be in touch if more

information is needed.

Nature Ecology & Evolution has now transitioned to a unified Rights Collection system which will allow our Author Services team to quickly and easily collect the rights and permissions required to publish your work. Approximately 10 days after your paper is formally accepted, you will receive an email in providing you with a link to complete the grant of rights. If your paper is eligible for Open Access, our Author Services team will also be in touch regarding any additional information that may be required to arrange payment for your article.

Please note that <i>Nature Ecology & Evolution</i> is a Transformative Journal (TJ). Authors may publish their research with us through the traditional subscription access route or make their paper immediately open access through payment of an article-processing charge (APC). Authors will not be required to make a final decision about access to their article until it has been accepted. Find out more about Transformative Journals

<B>Authors may need to take specific actions to achieve compliance with funder and institutional open access mandates. For submissions from January 2021, if your research is supported by a funder that requires immediate open access (e.g. according to Plan S principles) then you should select the gold OA route, and we will direct you to the compliant route where possible. For authors selecting the subscription publication route our standard licensing terms will need to be accepted, including our self-archiving policies. Those standard licensing terms will supersede any other terms that the author or any third party may assert apply to any version of the manuscript.

Please note that you will not receive your proofs until the publishing agreement has been received through our system.

For information regarding our different publishing models please see our Transformative Journals page. If you have any questions about costs, Open Access requirements, or our legal forms, please contact ASJournals@springernature.com.

Please use the following link for uploading these materials:
[REDACTED]

If you have any further questions, please feel free to contact me.

[REDACTED]

natureresearch

Final Decision Letter:

7th December 2021

Dear Dr Weigel,

We are pleased to inform you that your Article entitled "Commensal <i>Pseudomonas</i> strains facilitate protective response against pathogens in the host plant", has now been accepted for publication in Nature Ecology & Evolution.

Over the next few weeks, your paper will be copyedited to ensure that it conforms to Nature Ecology and Evolution style. Once your paper is typeset, you will receive an email with a link to choose the appropriate publishing options for your paper and our Author Services team will be in touch regarding any additional information that may be required

After the grant of rights is completed, you will receive a link to your electronic proof via email with a request to make any corrections within 48 hours. If, when you receive your proof, you cannot meet this deadline, please inform us at rjsproduction@springernature.com immediately.

You will not receive your proofs until the publishing agreement has been received through our system

Due to the importance of these deadlines, we ask you please us know now whether you will be difficult to contact over the next month. If this is the case, we ask you provide us with the contact information (email, phone and fax) of someone who will be able to check the proofs on your behalf, and who will be available to address any last-minute problems . Once your paper has been scheduled for online publication, the Nature press office will be in touch to confirm the details.

Acceptance of your manuscript is conditional on all authors' agreement with our publication policies (see www.nature.com/authors/policies/index.html). In particular your manuscript must not be published elsewhere and there must be no announcement of the work to any media outlet until the publication date (the day on which it is uploaded onto our web site).

Please note that <i>Nature Ecology & Evolution</i> is a Transformative Journal (TJ). Authors may publish their research with us through the traditional subscription access route or make their paper immediately open access through payment of an article-processing charge (APC). Authors will not be required to make a final decision about access to their article until it has been accepted. Find out more about Transformative Journals

<B>Authors may need to take specific actions to achieve compliance with funder and institutional open access mandates. For submissions from January 2021, if your research is supported by a funder that requires immediate open access (e.g. according to Plan S principles) then you should select the gold OA route, and we will direct you to the compliant route where possible. For authors selecting the subscription publication route our standard licensing terms will need to be accepted, including our <a href="https://www.springernature.com/gp/open-

research/policies/journal-policies">self-archiving policies. Those standard licensing terms will supersede any other terms that the author or any third party may assert apply to any version of the manuscript.

In approximately 10 business days you will receive an email with a link to choose the appropriate publishing options for your paper and our Author Services team will be in touch regarding any additional information that may be required.

You will not receive your proofs until the publishing agreement has been received through our system.

If you have any questions about our publishing options, costs, Open Access requirements, or our legal forms, please contact ASJournals@springernature.com

An online order form for reprints of your paper is available at https://www.nature.com/reprints/author-reprints.html. All co-authors, authors' institutions and authors' funding agencies can order reprints using the form appropriate to their geographical region.

We welcome the submission of potential cover material (including a short caption of around 40 words) related to your manuscript; suggestions should be sent to Nature Ecology & Evolution as electronic files (the image should be 300 dpi at 210 x 297 mm in either TIFF or JPEG format). Please note that such pictures should be selected more for their aesthetic appeal than for their scientific content, and that colour images work better than black and white or grayscale images. Please do not try to design a cover with the Nature Ecology & Evolution logo etc., and please do not submit composites of images related to your work. I am sure you will understand that we cannot make any promise as to whether any of your suggestions might be selected for the cover of the journal.

You can now use a single sign-on for all your accounts, view the status of all your manuscript submissions and reviews, access usage statistics for your published articles and download a record of your refereeing activity for the Nature journals.

To assist our authors in disseminating their research to the broader community, our SharedIt initiative provides you with a unique shareable link that will allow anyone (with or without a subscription) to read the published article. Recipients of the link with a subscription will also be able to download and print the PDF.

You can generate the link yourself when you receive your article DOI by entering it here: http://authors.springernature.com/share<a>.

[REDACTED]

P.S. Click on the following link if you would like to recommend Nature Ecology & Evolution to your librarian http://www.nature.com/subscriptions/recommend.html#forms

** Visit the Springer Nature Editorial and Publishing website at <a href="http://editorial-

jobs.springernature.com?utm_source=ejP_NEcoE_email&utm_medium=ejP_NEcoE_email&utm_campaign=ejp_NEcoE">www.springernature.com/editorial-and-publishing-jobs for more information about our career opportunities. If you have any questions please click here.**
